# Supplementary material for: Nonconsensus flanking sequence of hundreds of base pairs around in vivo binding sites: statistical beacons for transcription factor scanning
Source: Nucleic Acids Res. 2026 Jul 21;54(14):gkag514. doi: 10.1093/nar/gkag514 (PMC13386003; doi:10.1093/nar/gkag514)
Supplement: gkag514_Supplemental_File [file gkag514_supplemental_file.pdf]

Non-consensus flanking sequence of hundreds of base pairs around in vivo binding sites: statistical beacons for transcription factor scanning

Kateřina Faltejsková[1,2,✉], **Josef Šulc**[1,3], **and Jiří Vondrášek**[1,3,✉]

[1]Institute of Organic Chemistry and Biochemistry of the Czech Academy of Sciences, Prague 6 166 10, Czech Republic
[2]Computer Science Institute of Charles University, Faculty of Mathematics and Physics, Charles University, Malostranské náměstí 25, Prague 118 00, Czech Republic
[3]Faculty of Science, Charles University, Albertov 2038, Prague 128 00, Czech Republic

Supplementary materials

Supplementary Note S1: Materials and methods

Table S1. List of ChIP-seq experiments used, together with the target transcription factor, biosample identification and the corresponding number of peaks. If more peaks was available, the chosen 10 000 peaks are sampled randomly.

ENCODE file ID	ENCODE experiment ID	Target TF	Biosample name	Number of peaks
ENCFF966KGI	ENCSR000BHV	CTCF	A549	10000
ENCFF536RGD	ENCSR000AKB	CTCF	GM12878	10000
ENCFF017XLW	ENCSR000AKB	CTCF	GM12878	10000
ENCFF692RPA	ENCSR000AMF	CTCF	H1	10000
ENCFF252PLM	ENCSR000BIE	CTCF	HepG2	10000
ENCFF123DAC	ENCSR000BIE	CTCF	HepG2	10000
ENCFF769AUF	ENCSR000AKO	CTCF	K562	10000
ENCFF962IZR	ENCSR000DMO	CTCF	MCF-7	10000
ENCFF820BVW	ENCSR000DMO	CTCF	MCF-7	10000
ENCFF503DSN	ENCSR992XTY	CTCF	WTC11	10000
ENCFF855EOH	ENCSR861JUQ	FOXK2	GM12878	4761
ENCFF578XIP	ENCSR861JUQ	FOXK2	GM12878	4949
ENCFF008HBF	ENCSR171FUX	FOXK2	HepG2	8460
ENCFF997KQI	ENCSR171FUX	FOXK2	HepG2	7652
ENCFF022HGL	ENCSR302AWT	FOXK2	K562	10000
ENCFF154SRU	ENCSR302AWT	FOXK2	K562	10000
ENCFF583QAD	ENCSR465VLK	FOXK2	MCF-7	937
ENCFF356XXS	ENCSR465VLK	FOXK2	MCF-7	1028
ENCFF174QAR	ENCSR890DSP	IRF1	HepG2	2802
ENCFF019LOH	ENCSR854MCV	IRF1	K562	10000
ENCFF251ZSJ	ENCSR854MCV	IRF1	K562	10000
ENCFF910ZUK	ENCSR381IES	IRF1	WTC11	4632
ENCFF131JIX	ENCSR000BKB	MEF2A	GM12878	10000
ENCFF775DWU	ENCSR000BKB	MEF2A	GM12878	10000
ENCFF987BRO	ENCSR291MJH	MEF2A	HepG2	6412
ENCFF288TSN	ENCSR000BNV	MEF2A	K562	5016
ENCFF190IHG	ENCSR000BNV	MEF2A	K562	996
ENCFF674RQX	ENCSR000DYC	MYC	A549	9031
ENCFF356PWE	ENCSR000DYC	MYC	A549	8813
ENCFF765CKK	ENCSR000DKU	MYC	GM12878	2239
ENCFF372RQZ	ENCSR000EBY	MYC	H1	5205
ENCFF700CXD	ENCSR000EBY	MYC	H1	4987
ENCFF800JFG	ENCSR000DLR	MYC	HepG2	1616
ENCFF043WTJ	ENCSR744JJU	MYC	K562	7275
ENCFF149BIS	ENCSR000DMP	MYC	MCF-7	8306
ENCFF897LBK	ENCSR061DGF	NANOG	GM23338	8269
ENCFF463AFM	ENCSR061DGF	NANOG	GM23338	9475
ENCFF655XZF	ENCSR000BMT	NANOG	H1	5713
ENCFF722JFZ	ENCSR000BMT	NANOG	H1	5560
ENCFF252JYE	ENCSR145BHD	NFRKB	HepG2	1590
ENCFF748JYG	ENCSR145BHD	NFRKB	HepG2	1814
ENCFF594KPG	ENCSR657EOF	NFRKB	K562	10000
ENCFF048HYG	ENCSR657EOF	NFRKB	K562	10000
ENCFF360OXK	ENCSR248IMH	NFRKB	MCF-7	757
ENCFF768VQG	ENCSR248IMH	NFRKB	MCF-7	840
ENCFF264QLP	ENCSR414TYY	RUNX1	K562	3182
ENCFF015ADG	ENCSR414TYY	RUNX1	K562	3593

ENCFF881ARS	ENCSR000BGQ	SPI1	GM12878	10000
ENCFF198SPL	ENCSR000BGQ	SPI1	GM12878	10000
ENCFF185DGL	ENCSR000BGW	SPI1	K562	10000
ENCFF456BUE	ENCSR000BGW	SPI1	K562	10000
ENCFF488WQN	ENCSR112XUO	TP53	A549	5702
ENCFF701DTE	ENCSR980EGJ	TP53	HepG2	7662
ENCFF280RDQ	ENCSR760GZL	TP53	WTC11	7866

Table S2. List of ATAC-seq experiments used, together with the target biosample identification and the corresponding number of peaks.

ENCODE file ID	ENCODE experiment ID	Biosample name	Number of peaks
ENCFF653FJA	ENCSR139OYS	A549	211686
ENCFF899OMR	ENCSR032RGS	A549	278564
ENCFF665IBA	ENCSR074AHH	A549	206786
ENCFF139XJH	ENCSR265ZXX	A549	186872
ENCFF735UWS	ENCSR220ASC	A549	204227
ENCFF232BHQ	ENCSR288YMH	A549	210062
ENCFF748UZH	ENCSR637XSC	GM12878	277999
ENCFF470YYO	ENCSR095QNB	GM12878	223873
ENCFF567ZCX	ENCSR485TLP	GM23338	283236
ENCFF439EIO	ENCSR291GJU	HepG2	279739
ENCFF913MQB	ENCSR042AWH	HepG2	217283
ENCFF282BIU	ENCSR962TDN	K562	95499
ENCFF344PIT	ENCSR685YMG	K562	88200
ENCFF988RER	ENCSR343TQX	K562	122812
ENCFF243TXP	ENCSR644KPP	K562	115312
ENCFF839NAB	ENCSR978HFU	K562	79800
ENCFF200XYP	ENCSR091YVK	K562	142022
ENCFF812SFF	ENCSR935JVI	K562	119745
ENCFF558BLC	ENCSR483RKN	K562	203874
ENCFF477IQJ	ENCSR966ZCL	K562	138982
ENCFF586IMX	ENCSR179IAC	K562	77483
ENCFF274UER	ENCSR934FTO	K562	108381
ENCFF563WNA	ENCSR800RAH	K562	169113
ENCFF084PBF	ENCSR509MYW	K562	119694
ENCFF680FIO	ENCSR793YFK	K562	124858
ENCFF703WCG	ENCSR165JXS	K562	150255
ENCFF145AEP	ENCSR761CBY	K562	110274
ENCFF158GOJ	ENCSR243BXK	K562	105641
ENCFF550TGR	ENCSR822IYG	K562	114219
ENCFF529ZME	ENCSR121GEL	K562	106922
ENCFF092PVG	ENCSR017LGQ	K562	137656
ENCFF153CSM	ENCSR503EZG	K562	57092
ENCFF990VZL	ENCSR803QIE	K562	101766
ENCFF614WKT	ENCSR442DWQ	K562	113322
ENCFF517QIJ	ENCSR342GFK	K562	109055
ENCFF919ZPT	ENCSR582NPJ	K562	142156
ENCFF651JKF	ENCSR021GTX	K562	68526
ENCFF359EAN	ENCSR524ZSN	K562	85001
ENCFF481SJK	ENCSR465IDZ	K562	106861
ENCFF087HGO	ENCSR446UJL	K562	101869
ENCFF226XER	ENCSR874GAJ	K562	78919
ENCFF824NVT	ENCSR390UVH	K562	71350
ENCFF637YWY	ENCSR199HSR	K562	110733
ENCFF333TAT	ENCSR868FGK	K562	269800
ENCFF842UZU	ENCSR956DNB	K562	123697
ENCFF356SBB	ENCSR068MIW	K562	124886
ENCFF601WRI	ENCSR259HTB	K562	105071
ENCFF050STM	ENCSR096HVF	K562	112469
ENCFF392DVU	ENCSR680XOP	K562	109171
ENCFF339UPL	ENCSR304UWR	K562	67688
ENCFF968ZUO	ENCSR632VDU	K562	81082
ENCFF412STN	ENCSR281LEY	K562	110802
ENCFF215XQJ	ENCSR180IJO	K562	56164

ENCFF246XXM	ENCSR974QYY	K562	96675
ENCFF797KDM	ENCSR214QZO	K562	115418
ENCFF816LDC	ENCSR217QAB	K562	71100
ENCFF527ASE	ENCSR214SFA	K562	102484
ENCFF110ZGQ	ENCSR888JRM	K562	101726
ENCFF763PRE	ENCSR197GOQ	K562	104552
ENCFF962YNW	ENCSR741QNS	K562	80702
ENCFF429LOY	ENCSR676UFY	K562	53737
ENCFF603APA	ENCSR920YDG	K562	110912
ENCFF100HTV	ENCSR420NIU	K562	88732
ENCFF113CYL	ENCSR303NUP	K562	85264
ENCFF141VWA	ENCSR737HKX	K562	114684
ENCFF741UWF	ENCSR163VQD	K562	85780
ENCFF055NNT	ENCSR859USB	K562	99862
ENCFF252OYM	ENCSR314EDO	K562	123284
ENCFF291IVT	ENCSR522ALT	K562	141367
ENCFF415FXA	ENCSR296YAS	K562	84048
ENCFF984BJB	ENCSR508LLL	K562	61571
ENCFF239GPE	ENCSR745KUZ	K562	120757
ENCFF520WSX	ENCSR005BVE	K562	125390
ENCFF449NMH	ENCSR366CEQ	K562	128960
ENCFF891XBE	ENCSR548GGF	K562	87188
ENCFF821OEF	ENCSR422SUG	MCF-7	248668
ENCFF524JAA	ENCSR089KIJ	WTC11	197596
ENCFF321VDH	ENCSR541KFY	WTC11	264234

Supplementary Note S2: Results

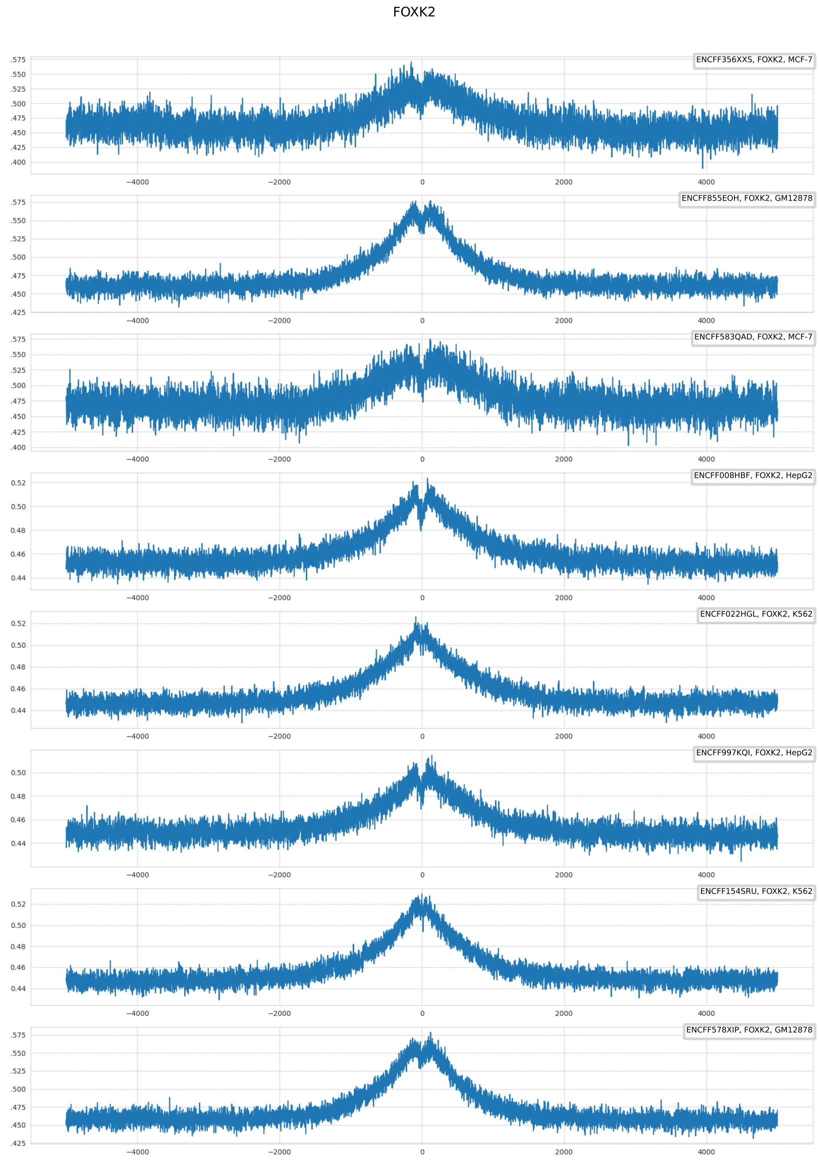

Fig. S1. Relation between GC content and distance from the putative FOXK2 binding site. On the y-axis, proportion of G or C base on given position can be found, while on the x-axis, number of base pairs from the putative binding site (ChIP-seq peak center).

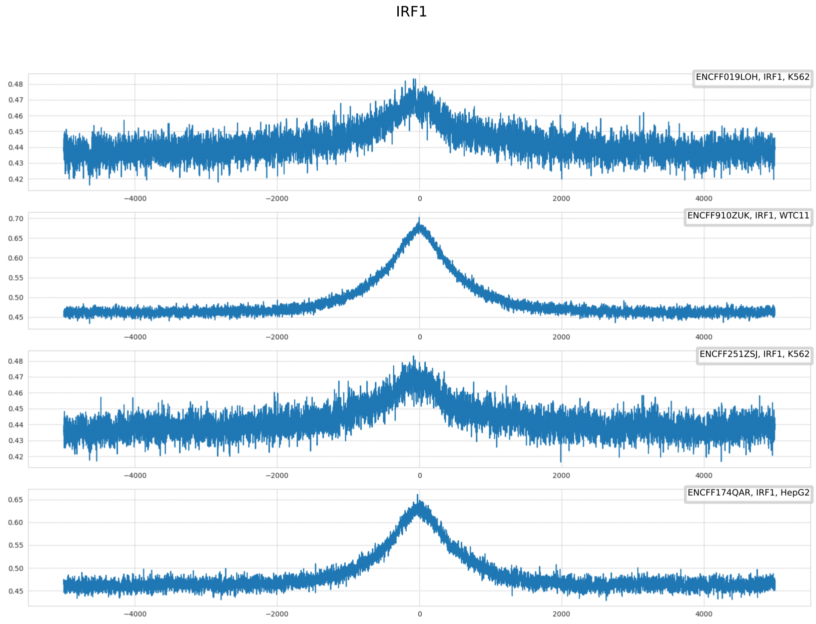

Fig. S2. Relation between GC content and distance from the putative IRF1 binding site. On the y-axis, proportion of G or C base on given position can be found, while on the x-axis, number of base pairs from the putative binding site (ChIP-seq peak center).

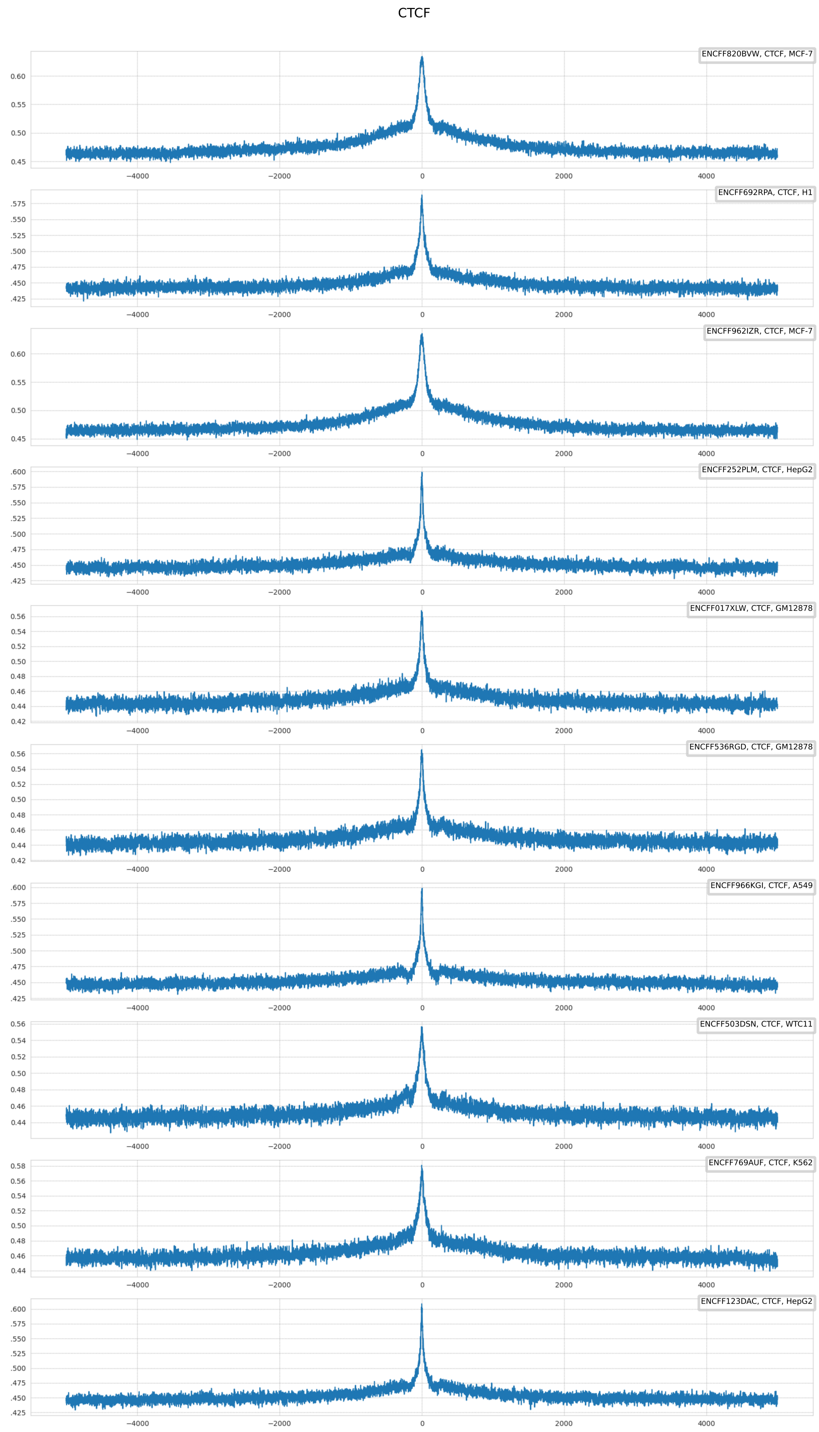

Fig. S3. Relation between GC content and distance from the putative CTCF binding site. On the y-axis, proportion of G or C base on given position can be found, while on the x-axis, number of base pairs from the putative binding site (ChIP-seq or Cut&Tag peak center).

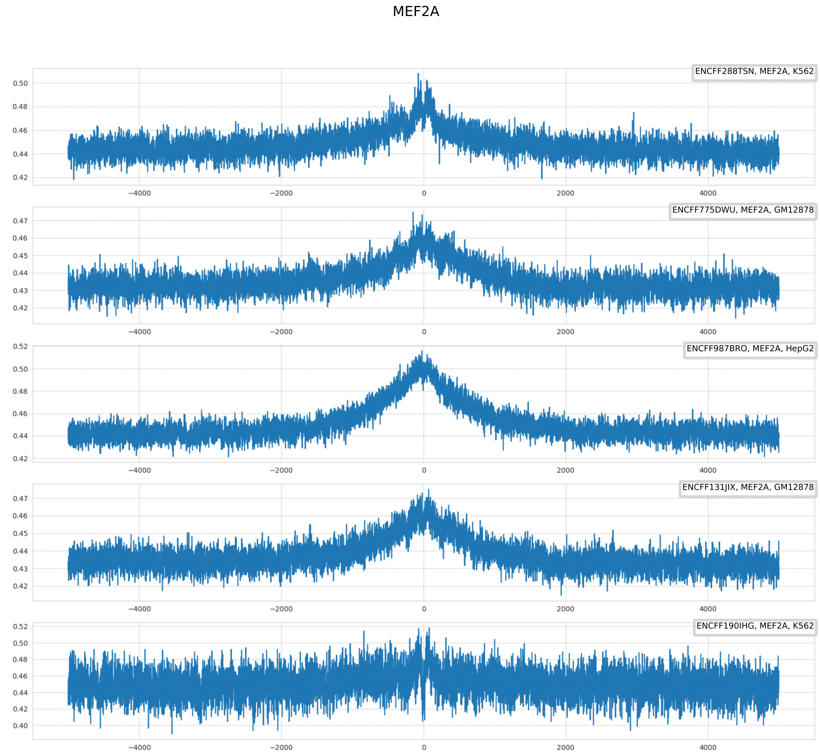

Fig. S4. Relation between GC content and distance from the putative MEF2A binding site. On the y-axis, proportion of G or C base on given position can be found, while on the x-axis, number of base pairs from the putative binding site (ChIP-seq peak center).

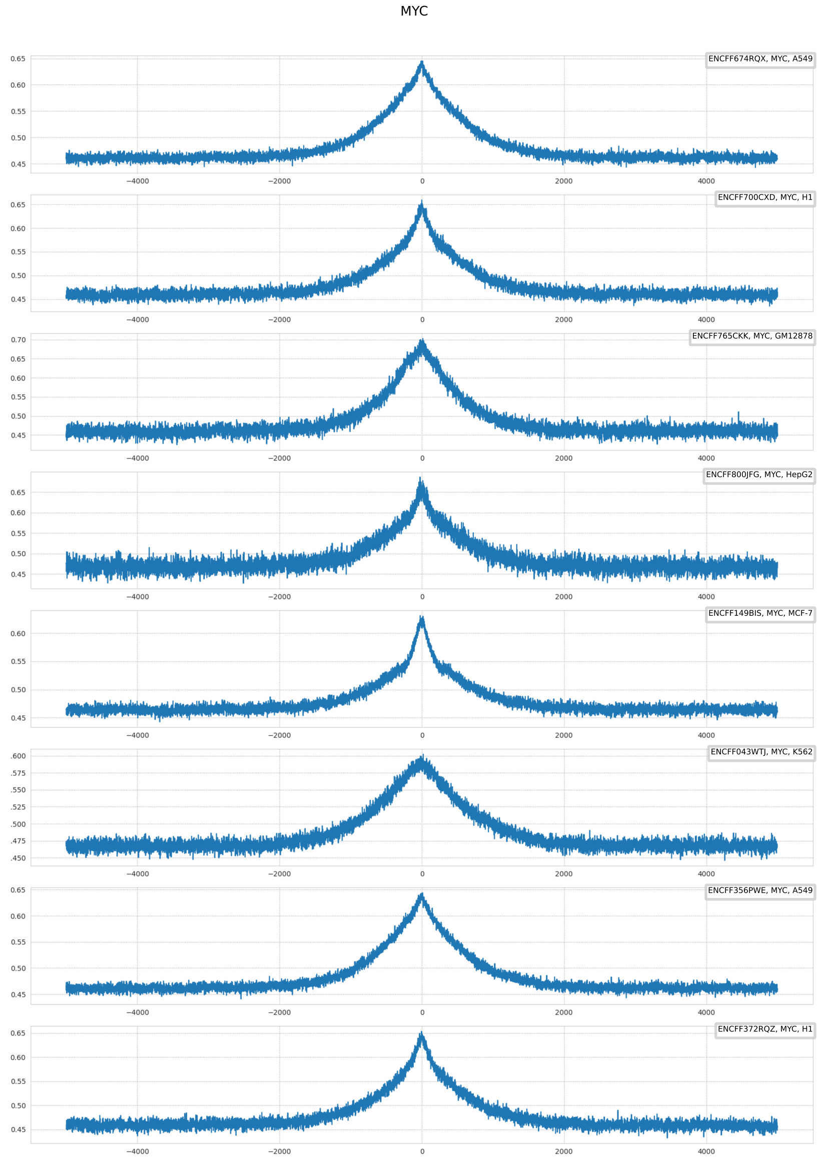

Fig. S5. Relation between GC content and distance from the putative MYC binding site. On the y-axis, proportion of G or C base on given position can be found, while on the x-axis, number of base pairs from the putative binding site (ChIP-seq peak center).

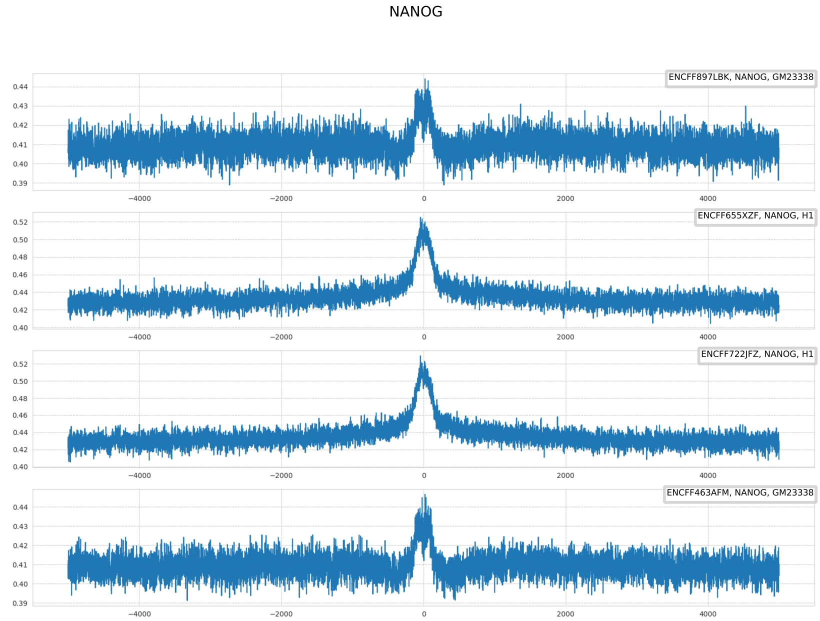

Fig. S6. Relation between GC content and distance from the putative NANOG binding site. On the y-axis, proportion of G or C base on given position can be found, while on the x-axis, number of base pairs from the putative binding site (ChIP-seq peak center).

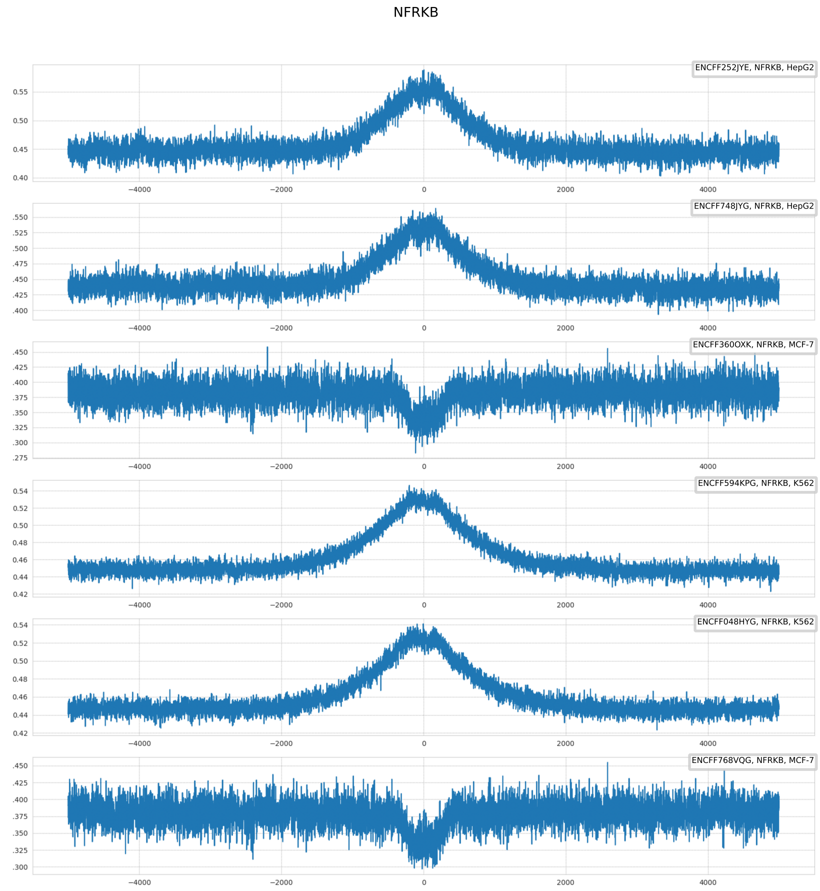

Fig. S7. Relation between GC content and distance from the putative NFRKB binding site. On the y-axis, proportion of G or C base on given position can be found, while on the x-axis, number of base pairs from the putative binding site (ChIP-seq peak center).

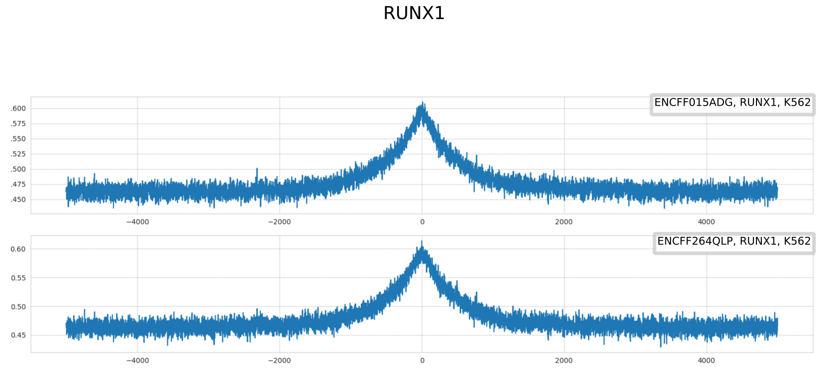

Fig. S8. Relation between GC content and distance from the putative RUNX1 binding site. On the y-axis, proportion of G or C base on given position can be found, while on the x-axis, number of base pairs from the putative binding site (ChIP-seq peak center).

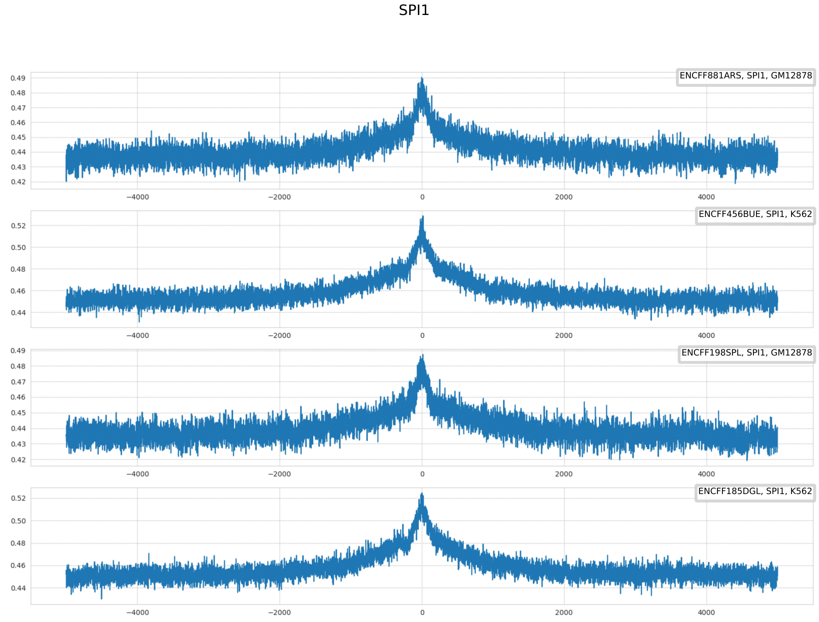

Fig. S9. Relation between GC content and distance from the putative SPI1 binding site. On the y-axis, proportion of G or C base on given position can be found, while on the x-axis, number of base pairs from the putative binding site (ChIP-seq peak center).

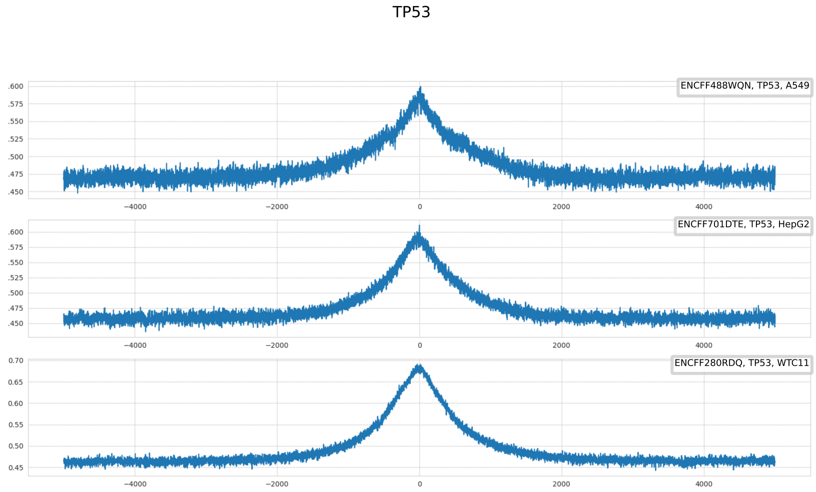

Fig. S10. Relation between GC content and distance from the putative TP53 binding site. On the y-axis, proportion of G or C base on given position can be found, while on the x-axis, number of base pairs from the putative binding site (ChIP-seq peak center).

Table S3. Coordinates (base pair offset w. r. to peak center) of the excessive patches in GC content identified by cluster analysis (see Materials and methods, Statistical analysis).

Excessive patch start	Excessive patch end	Cluster analysis p-value	Excess direction	Source	Target TF	Biosample name
2250	2400	$3.3 \cdot 10^{-2}$	increase	ENCFF966KGI	CTCF	A549
-1900	1800	$1.0 \cdot 10^{-3}$	increase	ENCFF966KGI	CTCF	A549
1875	2200	$2.0 \cdot 10^{-3}$	increase	ENCFF966KGI	CTCF	A549
-1775	1450	$1.0 \cdot 10^{-3}$	increase	ENCFF017XLW	CTCF	GM12878
1850	2050	$2.9 \cdot 10^{-2}$	increase	ENCFF017XLW	CTCF	GM12878
-3425	-3275	$4.9 \cdot 10^{-2}$	increase	ENCFF536RGD	CTCF	GM12878
-2725	-2450	$4.0 \cdot 10^{-3}$	increase	ENCFF536RGD	CTCF	GM12878
-2300	-1975	$1.0 \cdot 10^{-3}$	increase	ENCFF536RGD	CTCF	GM12878
-1900	1750	$1.0 \cdot 10^{-3}$	increase	ENCFF536RGD	CTCF	GM12878
2150	2550	$1.0 \cdot 10^{-3}$	increase	ENCFF536RGD	CTCF	GM12878
2650	2900	$3.0 \cdot 10^{-3}$	increase	ENCFF536RGD	CTCF	GM12878
-1775	2050	$1.0 \cdot 10^{-3}$	increase	ENCFF692RPA	CTCF	H1
-3975	-3775	$2.1 \cdot 10^{-2}$	increase	ENCFF692RPA	CTCF	H1
2075	2375	$7.0 \cdot 10^{-3}$	increase	ENCFF123DAC	CTCF	HepG2
-2400	-2150	$1.0 \cdot 10^{-2}$	increase	ENCFF123DAC	CTCF	HepG2
-1650	1800	$1.0 \cdot 10^{-3}$	increase	ENCFF123DAC	CTCF	HepG2
-2050	-1700	$2.0 \cdot 10^{-3}$	increase	ENCFF123DAC	CTCF	HepG2
3500	3700	$2.2 \cdot 10^{-2}$	increase	ENCFF252PLM	CTCF	HepG2
-1625	2400	$1.0 \cdot 10^{-3}$	increase	ENCFF252PLM	CTCF	HepG2
-2600	2800	$1.0 \cdot 10^{-3}$	increase	ENCFF769AUF	CTCF	K562
-2800	-2650	$5.0 \cdot 10^{-2}$	increase	ENCFF769AUF	CTCF	K562
-300	250	$1.0 \cdot 10^{-3}$	increase	GSM3560258	CTCF	K562
-2525	2325	$1.0 \cdot 10^{-3}$	increase	ENCFF820BVW	CTCF	MCF-7
-2850	2425	$1.0 \cdot 10^{-3}$	increase	ENCFF962IZR	CTCF	MCF-7
-2000	-1575	$1.0 \cdot 10^{-3}$	increase	ENCFF503DSN	CTCF	WTC11
-2725	-2450	$6.0 \cdot 10^{-3}$	increase	ENCFF503DSN	CTCF	WTC11
-3900	-3625	$6.0 \cdot 10^{-3}$	increase	ENCFF503DSN	CTCF	WTC11
-2400	-2075	$2.0 \cdot 10^{-3}$	increase	ENCFF503DSN	CTCF	WTC11
-1475	1800	$1.0 \cdot 10^{-3}$	increase	ENCFF503DSN	CTCF	WTC11
2250	2425	$3.4 \cdot 10^{-2}$	increase	ENCFF503DSN	CTCF	WTC11
-2000	525	$1.0 \cdot 10^{-3}$	increase	ENCFF578XIP	FOXK2	GM12878
-1425	1525	$1.0 \cdot 10^{-3}$	increase	ENCFF855EOH	FOXK2	GM12878
-1450	1900	$1.0 \cdot 10^{-3}$	increase	ENCFF008HBF	FOXK2	HepG2
-1400	275	$1.0 \cdot 10^{-3}$	increase	ENCFF997KQI	FOXK2	HepG2
-1775	1725	$1.0 \cdot 10^{-3}$	increase	ENCFF022HGL	FOXK2	K562
-1975	-1700	$1.7 \cdot 10^{-2}$	increase	ENCFF154SRU	FOXK2	K562
-1650	2150	$1.0 \cdot 10^{-3}$	increase	ENCFF154SRU	FOXK2	K562
-1275	200	$1.0 \cdot 10^{-3}$	increase	ENCFF356XXS	FOXK2	MCF-7
-1325	1425	$1.0 \cdot 10^{-3}$	increase	ENCFF583QAD	FOXK2	MCF-7
-1650	1575	$1.0 \cdot 10^{-3}$	increase	ENCFF174QAR	IRF1	HepG2
-2625	-2450	$1.4 \cdot 10^{-2}$	increase	ENCFF019LOH	IRF1	K562
-2300	-2150	$3.1 \cdot 10^{-2}$	increase	ENCFF019LOH	IRF1	K562
-2100	-1675	$1.0 \cdot 10^{-3}$	increase	ENCFF019LOH	IRF1	K562
-1625	1725	$1.0 \cdot 10^{-3}$	increase	ENCFF019LOH	IRF1	K562
1800	2550	$1.0 \cdot 10^{-3}$	increase	ENCFF019LOH	IRF1	K562
1775	2000	$1.6 \cdot 10^{-2}$	increase	ENCFF251ZSJ	IRF1	K562
-2575	-2225	$3.0 \cdot 10^{-3}$	increase	ENCFF251ZSJ	IRF1	K562
2250	2550	$6.0 \cdot 10^{-3}$	increase	ENCFF251ZSJ	IRF1	K562
-1175	1650	$1.0 \cdot 10^{-3}$	increase	ENCFF251ZSJ	IRF1	K562
-1850	1950	$1.0 \cdot 10^{-3}$	increase	ENCFF910ZUK	IRF1	WTC11
25	475	$1.0 \cdot 10^{-3}$	increase	ENCFF131JIX	MEF2A	GM12878

-650	-25	$1.0 \cdot 10^{-3}$	increase	ENCFF131JIX	MEF2A	GM12878
-1100	-925	$1.7 \cdot 10^{-2}$	increase	ENCFF775DWU	MEF2A	GM12878
-1625	-1475	$3.2 \cdot 10^{-2}$	increase	ENCFF775DWU	MEF2A	GM12878
-825	1000	$1.0 \cdot 10^{-3}$	increase	ENCFF775DWU	MEF2A	GM12878
-1375	1350	$1.0 \cdot 10^{-3}$	increase	ENCFF987BRO	MEF2A	HepG2
-900	-250	$1.0 \cdot 10^{-3}$	increase	ENCFF190IHG	MEF2A	K562
425	575	$4.0 \cdot 10^{-2}$	increase	ENCFF190IHG	MEF2A	K562
25	225	$6.0 \cdot 10^{-3}$	increase	ENCFF190IHG	MEF2A	K562
-175	-25	$9.0 \cdot 10^{-3}$	increase	ENCFF190IHG	MEF2A	K562
-1300	700	$1.0 \cdot 10^{-3}$	increase	ENCFF288TSN	MEF2A	K562
1325	1500	$1.3 \cdot 10^{-2}$	increase	ENCFF288TSN	MEF2A	K562
750	1100	$4.0 \cdot 10^{-3}$	increase	ENCFF288TSN	MEF2A	K562
-2175	-1875	$2.3 \cdot 10^{-2}$	increase	ENCFF356PWE	MYC	A549
-1825	1850	$1.0 \cdot 10^{-3}$	increase	ENCFF356PWE	MYC	A549
-2200	1800	$1.0 \cdot 10^{-3}$	increase	ENCFF674RQX	MYC	A549
-1475	1500	$1.0 \cdot 10^{-3}$	increase	ENCFF765CKK	MYC	GM12878
-1575	375	$1.0 \cdot 10^{-3}$	increase	ENCFF372RQZ	MYC	H1
-1625	1825	$1.0 \cdot 10^{-3}$	increase	ENCFF700CXD	MYC	H1
-1900	-1700	$3.8 \cdot 10^{-2}$	increase	ENCFF700CXD	MYC	H1
-1675	1700	$1.0 \cdot 10^{-3}$	increase	ENCFF800JFG	MYC	HepG2
2000	2275	$1.7 \cdot 10^{-2}$	increase	ENCFF800JFG	MYC	HepG2
-1750	1675	$1.0 \cdot 10^{-3}$	increase	ENCFF043WTJ	MYC	K562
-1700	1825	$1.0 \cdot 10^{-3}$	increase	ENCFF149BIS	MYC	MCF-7
2000	2225	$1.8 \cdot 10^{-2}$	increase	ENCFF149BIS	MYC	MCF-7
175	700	$1.0 \cdot 10^{-3}$	decrease	ENCFF463AFM	NANOG	GM23338
-600	-275	$2.0 \cdot 10^{-3}$	decrease	ENCFF463AFM	NANOG	GM23338
-125	125	$1.0 \cdot 10^{-3}$	increase	ENCFF463AFM	NANOG	GM23338
175	700	$1.0 \cdot 10^{-3}$	decrease	ENCFF897LBK	NANOG	GM23338
-125	125	$1.0 \cdot 10^{-3}$	increase	ENCFF897LBK	NANOG	GM23338
-475	-275	$6.0 \cdot 10^{-3}$	decrease	ENCFF897LBK	NANOG	GM23338
650	1450	$1.0 \cdot 10^{-3}$	increase	ENCFF655XZF	NANOG	H1
-475	500	$1.0 \cdot 10^{-3}$	increase	ENCFF655XZF	NANOG	H1
-675	-525	$2.3 \cdot 10^{-2}$	increase	ENCFF655XZF	NANOG	H1
-900	-725	$2.3 \cdot 10^{-2}$	increase	ENCFF655XZF	NANOG	H1
-475	150	$1.0 \cdot 10^{-3}$	increase	ENCFF722JFZ	NANOG	H1
-675	-525	$3.0 \cdot 10^{-2}$	increase	ENCFF722JFZ	NANOG	H1
-1025	-750	$5.0 \cdot 10^{-3}$	increase	ENCFF722JFZ	NANOG	H1
-1575	-1300	$9.0 \cdot 10^{-3}$	increase	ENCFF722JFZ	NANOG	H1
-1225	1375	$1.0 \cdot 10^{-3}$	increase	ENCFF252JYE	NFRKB	HepG2
-1200	500	$1.0 \cdot 10^{-3}$	increase	ENCFF748JYG	NFRKB	HepG2
-3125	1775	$1.0 \cdot 10^{-3}$	increase	ENCFF048HYG	NFRKB	K562
2175	2900	$1.0 \cdot 10^{-3}$	increase	ENCFF048HYG	NFRKB	K562
1850	2125	$1.0 \cdot 10^{-2}$	increase	ENCFF048HYG	NFRKB	K562
-2900	-2100	$1.0 \cdot 10^{-3}$	increase	ENCFF594KPG	NFRKB	K562
-2050	2850	$1.0 \cdot 10^{-3}$	increase	ENCFF594KPG	NFRKB	K562
-300	325	$1.0 \cdot 10^{-3}$	decrease	ENCFF360OXK	NFRKB	MCF-7
-325	325	$1.0 \cdot 10^{-3}$	decrease	ENCFF768VQG	NFRKB	MCF-7
1650	1875	$3.3 \cdot 10^{-2}$	increase	ENCFF015ADG	RUNX1	K562
-1325	1400	$1.0 \cdot 10^{-3}$	increase	ENCFF015ADG	RUNX1	K562
-1325	1225	$1.0 \cdot 10^{-3}$	increase	ENCFF264QLP	RUNX1	K562
-1650	-1475	$3.3 \cdot 10^{-2}$	increase	ENCFF198SPL	SPI1	GM12878
-1425	1425	$1.0 \cdot 10^{-3}$	increase	ENCFF198SPL	SPI1	GM12878
-1150	1375	$1.0 \cdot 10^{-3}$	increase	ENCFF881ARS	SPI1	GM12878
-2325	-2150	$4.2 \cdot 10^{-2}$	increase	ENCFF185DGL	SPI1	K562
-1925	1575	$1.0 \cdot 10^{-3}$	increase	ENCFF185DGL	SPI1	K562

1625	1775	$2.6 \cdot 10^{-2}$	increase	ENCFF185DGL	SPI1	K562
-1575	1825	$1.0 \cdot 10^{-3}$	increase	ENCFF456BUE	SPI1	K562
-1925	-1650	$1.0 \cdot 10^{-2}$	increase	ENCFF456BUE	SPI1	K562
-1725	1600	$1.0 \cdot 10^{-3}$	increase	ENCFF488WQN	TP53	A549
-1650	1725	$1.0 \cdot 10^{-3}$	increase	ENCFF701DTE	TP53	HepG2
2450	2750	$3.9 \cdot 10^{-2}$	increase	ENCFF280RDQ	TP53	WTC11
-1900	2175	$1.0 \cdot 10^{-3}$	increase	ENCFF280RDQ	TP53	WTC11

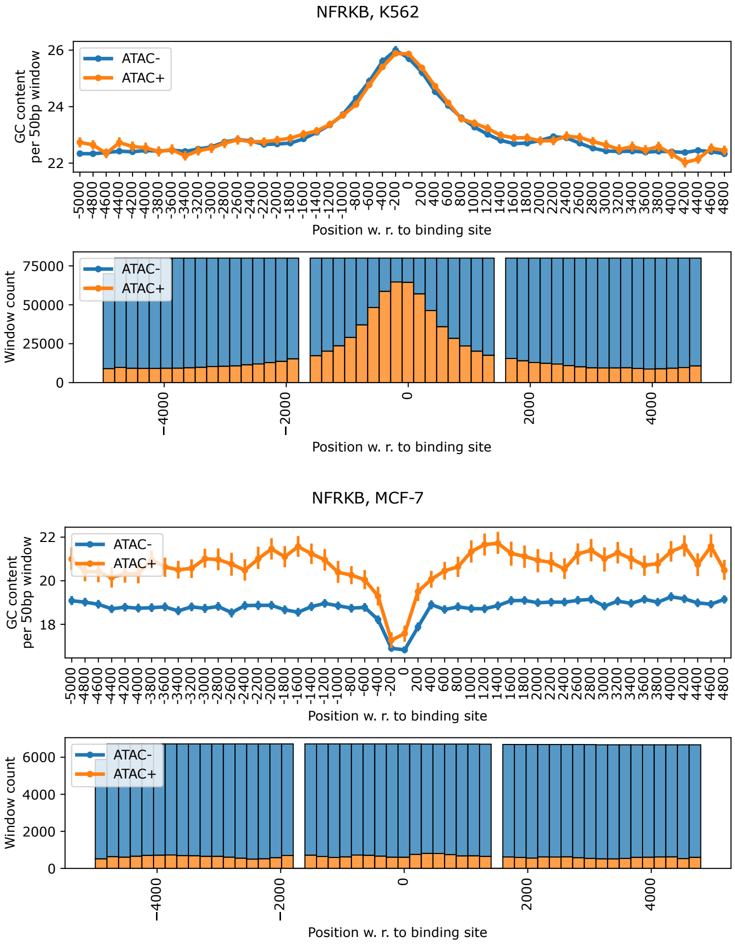

Fig. S11. Overview of ATAC-seq and ChIP-seq combined information on NFRKB in K562 (top pair of plots) and in MCF-7 (bottom pair of plots). In the top plot, the average GC content (with 95% confidence interval shown as error bars) by position with respect to the binding site, distinguished for k-mers overlapping with an ATAC-seq peak (ATAC+) and k-mers not overlapping with an ATAC-seq peak (ATAC-). In the bottom plot, the distribution of ATAC+ aand ATAC- k-mers per position.

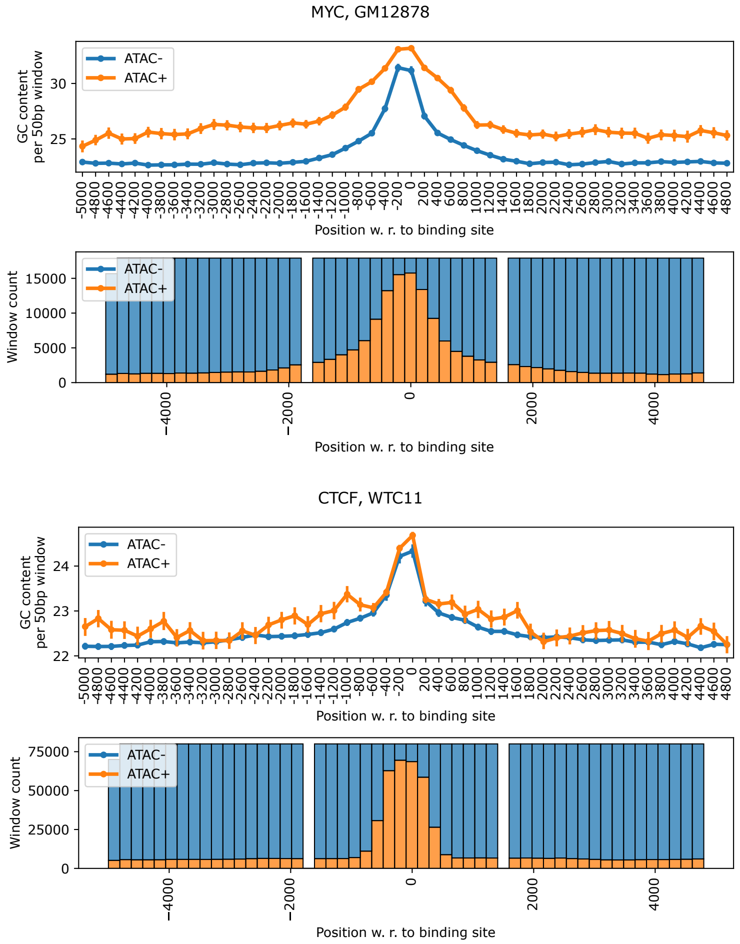

Fig. S12. Overview of ATAC-seq and ChIP-seq combined information on MYC in GM12878 (top pair of plots) and on CTCF in WTC11 (bottom pair of plots). In the top plot, the average GC content (with 95% confidence interval shown as error bars) by position with respect to the binding site, distinguished for k-mers overlapping with an ATAC-seq peak (ATAC+) and k-mers not overlapping with an ATAC-seq peak (ATAC-). In the bottom plot, the distribution of ATAC+ aand ATAC- k-mers per position.

Table S4. Average GC content for k-mers overlapping with an ATAC-seq peak (ATAC+) and k-mers not overlapping with an ATAC-seq peak (ATAC-) per chosen positions (w. r. to the binding site). In every cell, first value is the average GC content in ATAC+ windows, second one described the average GC content in ATAC- windows. Statistical significance (Mann-Whitney U test with Holm correction for multiple testing for ATAC+ and ATAC- windows, "greater" alternative hypothesis) encoded as stars: $^{****}p < 10^{-4}$, $^{***}p < 10^{-3}$, $^{**}p < 10^{-2}$, $^{*}p < 0.05$; ns omitted.

ENCODE file ID	Target TF	Cell line	-3000	-1000	0	1000	3000
ENCFF966KGI	CTCF	A549	25.9 (s. d. 6.5) / 22.0 (s. d. 6.2)****	26.3 (s. d. 7.1) / 22.2 (s. d. 6.3)****	25.0 (s. d. 6.9) / 21.6 (s. d. 6.9)****	26.2 (s. d. 6.9) / 22.2 (s. d. 6.3)****	26.2 (s. d. 6.8) / 22.0 (s. d. 6.3)****
ENCFF017XLW	CTCF	GM12878	23.5 (s. d. 6.7) / 22.1 (s. d. 6.3)****	23.9 (s. d. 7.1) / 22.6 (s. d. 6.5)****	24.6 (s. d. 6.8) / 24.3 (s. d. 6.7)****	23.8 (s. d. 6.9) / 22.4 (s. d. 6.5)****	23.3 (s. d. 6.9) / 22.1 (s. d. 6.3)****
ENCFF536RGD	CTCF	GM12878	24.5 (s. d. 6.8) / 22.0 (s. d. 6.3)****	25.7 (s. d. 7.5) / 22.3 (s. d. 6.4)****	24.6 (s. d. 6.8) / 23.7 (s. d. 6.7)****	25.2 (s. d. 7.1) / 22.3 (s. d. 6.3)****	24.9 (s. d. 7.2) / 22.0 (s. d. 6.3)****
ENCFF123DAC	CTCF	HepG2	23.8 (s. d. 6.8) / 22.2 (s. d. 6.4)****	24.4 (s. d. 7.0) / 22.6 (s. d. 6.5)****	25.2 (s. d. 6.7) / 24.1 (s. d. 6.8)****	24.1 (s. d. 6.8) / 22.6 (s. d. 6.5)****	23.8 (s. d. 6.8) / 22.3 (s. d. 6.4)****
ENCFF252PLM	CTCF	HepG2	23.1 (s. d. 6.6) / 22.3 (s. d. 6.4)****	23.7 (s. d. 6.6) / 22.8 (s. d. 6.6)****	24.9 (s. d. 6.7) / 24.4 (s. d. 6.7)****	23.7 (s. d. 6.9) / 22.6 (s. d. 6.5)****	23.2 (s. d. 6.7) / 22.3 (s. d. 6.4)****
ENCFF769AUF	CTCF	K562	22.8 (s. d. 6.4) / 22.8 (s. d. 6.4)	23.6 (s. d. 6.5) / 23.3 (s. d. 6.6)***	25.2 (s. d. 6.5) / 24.6 (s. d. 6.5)****	23.3 (s. d. 6.4) / 23.2 (s. d. 6.5)	23.1 (s. d. 6.6) / 22.8 (s. d. 6.4)**
ENCFF820BVW	CTCF	MCF-7	23.6 (s. d. 6.7) / 23.3 (s. d. 6.4)*	24.8 (s. d. 7.0) / 24.2 (s. d. 6.7)****	27.7 (s. d. 6.4) / 27.6 (s. d. 6.4)	24.8 (s. d. 6.6) / 24.0 (s. d. 6.6)****	24.0 (s. d. 6.6) / 23.2 (s. d. 6.4)****
ENCFF962IZR	CTCF	MCF-7	23.3 (s. d. 6.6) / 23.4 (s. d. 6.4)	24.2 (s. d. 6.8) / 24.3 (s. d. 6.7)	27.7 (s. d. 6.4) / 27.6 (s. d. 6.4)	24.0 (s. d. 6.5) / 24.1 (s. d. 6.6)	23.4 (s. d. 6.5) / 23.3 (s. d. 6.4)
ENCFF503DSN	CTCF	WTC11	22.3 (s. d. 6.3) / 22.3 (s. d. 6.4)	23.4 (s. d. 6.7) / 22.7 (s. d. 6.6)****	24.7 (s. d. 6.9) / 24.3 (s. d. 6.7)***	23.0 (s. d. 6.7) / 22.6 (s. d. 6.5)**	22.6 (s. d. 6.6) / 22.3 (s. d. 6.4)
ENCFF578XIP	FOXK2	GM12878	23.1 (s. d. 6.5) / 23.0 (s. d. 6.5)	24.6 (s. d. 6.8) / 24.2 (s. d. 6.7)****	27.4 (s. d. 6.6) / 26.8 (s. d. 6.5)****	23.8 (s. d. 6.7) / 23.8 (s. d. 6.7)	23.0 (s. d. 6.4) / 23.0 (s. d. 6.5)
ENCFF855EOH	FOXK2	GM12878	23.3 (s. d. 6.2) / 23.1 (s. d. 6.3)	24.4 (s. d. 6.4) / 24.3 (s. d. 6.7)	27.9 (s. d. 6.4) / 27.0 (s. d. 6.2)****	24.1 (s. d. 6.9) / 23.9 (s. d. 6.5)	23.2 (s. d. 6.3) / 23.1 (s. d. 6.3)
ENCFF008HBF	FOXK2	HepG2	22.6 (s. d. 6.4) / 22.7 (s. d. 6.3)	23.6 (s. d. 6.6) / 23.4 (s. d. 6.5)	25.2 (s. d. 6.2) / 24.9 (s. d. 6.1)***	23.4 (s. d. 6.4) / 23.2 (s. d. 6.4)*	22.6 (s. d. 6.3) / 22.7 (s. d. 6.3)
ENCFF997KQI	FOXK2	HepG2	22.5 (s. d. 6.2) / 22.5 (s. d. 6.4)	23.1 (s. d. 6.6) / 23.2 (s. d. 6.5)	24.9 (s. d. 6.1) / 23.9 (s. d. 5.7)****	23.3 (s. d. 6.4) / 22.9 (s. d. 6.4)****	22.5 (s. d. 6.6) / 22.4 (s. d. 6.4)

ENCFF022HGL	FOXK2	K562	22.5 (s. d. 6.4) / 22.3 (s. d. 6.4)*	23.4 (s. d. 6.8) / 23.2 (s. d. 6.8)	25.2 (s. d. 6.4) / 25.0 (s. d. 6.5)	23.0 (s. d. 6.7) / 23.0 (s. d. 6.6)	22.6 (s. d. 6.4) / 22.4 (s. d. 6.3)
ENCFF154SRU	FOXK2	K562	22.3 (s. d. 6.3) / 22.3 (s. d. 6.4)	23.4 (s. d. 6.8) / 23.3 (s. d. 6.7)	25.6 (s. d. 6.4) / 25.5 (s. d. 6.4)	23.1 (s. d. 6.8) / 23.0 (s. d. 6.6)	22.6 (s. d. 6.4) / 22.4 (s. d. 6.3)**
ENCFF356XXS	FOXK2	MCF-7	25.5 (s. d. 6.3) / 22.7 (s. d. 6.6)****	26.3 (s. d. 6.3) / 23.5 (s. d. 6.6)****	29.2 (s. d. 6.6) / 23.6 (s. d. 5.2)****	26.1 (s. d. 6.5) / 23.5 (s. d. 6.4)****	26.5 (s. d. 6.5) / 22.7 (s. d. 6.6)****
ENCFF583QAD	FOXK2	MCF-7	24.7 (s. d. 6.8) / 23.4 (s. d. 6.3)***	23.8 (s. d. 6.3) / 24.4 (s. d. 6.4)	28.0 (s. d. 6.7) / 24.8 (s. d. 5.6)****	23.8 (s. d. 6.0) / 24.3 (s. d. 6.4)	23.1 (s. d. 6.1) / 23.2 (s. d. 6.2)
ENCFF174QAR	IRF1	HepG2	27.5 (s. d. 6.8) / 22.7 (s. d. 6.3)****	28.7 (s. d. 7.1) / 23.8 (s. d. 6.6)****	31.3 (s. d. 6.0) / 28.0 (s. d. 6.3)****	27.8 (s. d. 7.0) / 23.5 (s. d. 6.3)****	27.6 (s. d. 6.7) / 22.8 (s. d. 6.2)****
ENCFF019LOH	IRF1	K562	21.9 (s. d. 6.3) / 21.9 (s. d. 6.2)	22.4 (s. d. 6.6) / 22.3 (s. d. 6.5)	23.0 (s. d. 6.2) / 23.2 (s. d. 6.1)	22.2 (s. d. 6.3) / 22.3 (s. d. 6.4)	21.9 (s. d. 6.3) / 22.0 (s. d. 6.2)
ENCFF251ZSJ	IRF1	K562	21.7 (s. d. 6.2) / 21.9 (s. d. 6.2)	22.4 (s. d. 6.6) / 22.4 (s. d. 6.5)	23.1 (s. d. 6.2) / 23.1 (s. d. 6.2)	22.3 (s. d. 6.4) / 22.2 (s. d. 6.4)	22.0 (s. d. 6.3) / 22.0 (s. d. 6.2)
ENCFF910ZUK	IRF1	WTC11	28.2 (s. d. 6.5) / 22.8 (s. d. 6.3)****	30.9 (s. d. 6.9) / 24.4 (s. d. 6.7)****	33.4 (s. d. 5.3) / 31.6 (s. d. 5.8)****	29.9 (s. d. 7.1) / 24.1 (s. d. 6.5)****	28.0 (s. d. 6.9) / 22.8 (s. d. 6.3)****
ENCFF131JIX	MEF2A	GM12878	23.2 (s. d. 6.7) / 21.5 (s. d. 6.1)****	23.3 (s. d. 6.6) / 21.7 (s. d. 6.2)****	23.0 (s. d. 6.8) / 22.4 (s. d. 7.0)****	23.0 (s. d. 6.7) / 21.7 (s. d. 6.2)****	22.9 (s. d. 6.4) / 21.5 (s. d. 6.2)****
ENCFF775DWU	MEF2A	GM12878	23.3 (s. d. 6.6) / 21.3 (s. d. 6.0)****	23.2 (s. d. 6.6) / 21.6 (s. d. 6.2)****	23.0 (s. d. 6.7) / 21.6 (s. d. 6.7)****	23.0 (s. d. 6.7) / 21.6 (s. d. 6.1)****	23.1 (s. d. 6.5) / 21.3 (s. d. 6.0)****
ENCFF987BRO	MEF2A	HepG2	22.4 (s. d. 6.5) / 22.1 (s. d. 6.3)	22.9 (s. d. 6.6) / 22.9 (s. d. 6.7)	24.8 (s. d. 7.1) / 24.5 (s. d. 7.3)	22.6 (s. d. 6.4) / 22.6 (s. d. 6.5)	22.3 (s. d. 6.5) / 22.2 (s. d. 6.2)
ENCFF190IHG	MEF2A	K562	24.4 (s. d. 6.9) / 22.1 (s. d. 6.2)****	25.1 (s. d. 6.8) / 22.1 (s. d. 6.4)****	23.5 (s. d. 6.2) / 21.2 (s. d. 6.1)****	24.3 (s. d. 6.7) / 22.1 (s. d. 6.2)****	24.6 (s. d. 6.3) / 21.9 (s. d. 6.2)****
ENCFF288TSN	MEF2A	K562	24.6 (s. d. 6.7) / 21.8 (s. d. 6.2)****	25.1 (s. d. 6.9) / 21.9 (s. d. 6.3)****	24.3 (s. d. 6.4) / 20.7 (s. d. 5.7)****	24.8 (s. d. 6.9) / 21.8 (s. d. 6.3)****	24.7 (s. d. 6.6) / 21.7 (s. d. 6.2)****
ENCFF356PWE	MYC	A549	23.2 (s. d. 6.3) / 23.1 (s. d. 6.3)	25.1 (s. d. 7.0) / 25.1 (s. d. 7.0)	30.6 (s. d. 6.8) / 30.7 (s. d. 6.9)	24.5 (s. d. 6.8) / 24.5 (s. d. 6.9)	23.2 (s. d. 6.4) / 23.1 (s. d. 6.4)

ENCFF674RQX	MYC	A549	25.0 (s. d. 6.7) / 23.0 (s. d. 6.2)****	26.5 (s. d. 7.5) / 24.5 (s. d. 6.8)****	30.8 (s. d. 6.8) / 29.2 (s. d. 7.1)****	26.0 (s. d. 7.2) / 24.0 (s. d. 6.7)****	25.1 (s. d. 7.0) / 22.9 (s. d. 6.3)****
ENCFF765CKK	MYC	GM12878	26.3 (s. d. 7.2) / 22.9 (s. d. 6.2)****	27.9 (s. d. 7.4) / 24.2 (s. d. 6.8)****	33.2 (s. d. 5.3) / 31.2 (s. d. 6.3)****	26.2 (s. d. 7.4) / 23.9 (s. d. 6.6)****	25.6 (s. d. 6.9) / 23.0 (s. d. 6.3)****
ENCFF800JFG	MYC	HepG2	26.8 (s. d. 6.5) / 23.1 (s. d. 6.3)****	28.7 (s. d. 7.1) / 24.0 (s. d. 6.4)****	31.2 (s. d. 6.2) / 29.8 (s. d. 6.8)****	28.5 (s. d. 6.9) / 23.6 (s. d. 6.3)****	27.1 (s. d. 6.5) / 23.0 (s. d. 6.3)****
ENCFF043WTJ	MYC	K562	23.6 (s. d. 6.5) / 23.4 (s. d. 6.4)	25.3 (s. d. 7.1) / 25.1 (s. d. 7.2)	29.1 (s. d. 6.2) / 28.5 (s. d. 6.3)****	24.7 (s. d. 6.9) / 24.7 (s. d. 6.9)	23.5 (s. d. 6.6) / 23.4 (s. d. 6.4)
ENCFF149BIS	MYC	MCF-7	26.3 (s. d. 6.4) / 23.0 (s. d. 6.3)****	28.9 (s. d. 7.2) / 24.0 (s. d. 6.5)****	30.2 (s. d. 5.9) / 27.4 (s. d. 6.3)****	28.0 (s. d. 7.1) / 23.8 (s. d. 6.4)****	26.5 (s. d. 6.6) / 23.0 (s. d. 6.3)****
ENCFF463AFM	NANOG	GM23338	23.9 (s. d. 6.8) / 20.1 (s. d. 5.7)****	22.9 (s. d. 6.5) / 19.9 (s. d. 5.6)****	21.7 (s. d. 5.2) / 19.6 (s. d. 5.0)****	22.7 (s. d. 6.6) / 20.1 (s. d. 5.8)****	23.9 (s. d. 6.9) / 20.2 (s. d. 5.7)****
ENCFF897LBK	NANOG	GM23338	24.0 (s. d. 7.0) / 20.1 (s. d. 5.7)****	23.0 (s. d. 6.6) / 20.0 (s. d. 5.7)****	21.7 (s. d. 5.3) / 19.3 (s. d. 4.9)****	22.7 (s. d. 6.6) / 20.1 (s. d. 5.8)****	23.8 (s. d. 6.9) / 20.2 (s. d. 5.7)****
ENCFF252JYE	NFRKB	HepG2	22.8 (s. d. 6.1) / 22.4 (s. d. 6.3)	23.3 (s. d. 6.4) / 23.6 (s. d. 6.5)	28.2 (s. d. 6.3) / 26.5 (s. d. 6.5)****	23.4 (s. d. 6.1) / 23.1 (s. d. 6.5)	22.5 (s. d. 6.2) / 22.1 (s. d. 6.4)
ENCFF748JYG	NFRKB	HepG2	22.3 (s. d. 6.7) / 22.0 (s. d. 6.4)	23.7 (s. d. 6.7) / 22.9 (s. d. 6.4)****	27.7 (s. d. 6.4) / 24.8 (s. d. 6.2)****	23.0 (s. d. 6.3) / 22.7 (s. d. 6.6)	22.4 (s. d. 6.1) / 21.8 (s. d. 6.6)*
ENCFF048HYG	NFRKB	K562	22.8 (s. d. 6.4) / 22.6 (s. d. 6.4)**	23.6 (s. d. 7.0) / 23.6 (s. d. 7.0)	25.6 (s. d. 6.6) / 25.7 (s. d. 6.7)	23.3 (s. d. 6.7) / 23.1 (s. d. 6.8)****	22.7 (s. d. 6.7) / 22.4 (s. d. 6.4)****
ENCFF594KPG	NFRKB	K562	22.5 (s. d. 6.5) / 22.6 (s. d. 6.5)	23.7 (s. d. 7.0) / 23.7 (s. d. 7.0)	25.9 (s. d. 6.7) / 25.7 (s. d. 6.5)	23.4 (s. d. 6.7) / 23.3 (s. d. 6.8)	22.6 (s. d. 6.6) / 22.4 (s. d. 6.4)
ENCFF360OXK	NFRKB	MCF-7	21.1 (s. d. 4.7) / 18.9 (s. d. 5.4)****	20.6 (s. d. 5.6) / 18.9 (s. d. 5.3)****	17.5 (s. d. 3.8) / 16.9 (s. d. 3.8)***	21.2 (s. d. 5.4) / 18.9 (s. d. 5.3)****	20.6 (s. d. 5.2) / 18.9 (s. d. 5.3)****
ENCFF768VQG	NFRKB	MCF-7	21.0 (s. d. 4.9) / 18.7 (s. d. 5.7)****	20.4 (s. d. 5.3) / 18.9 (s. d. 5.6)****	17.6 (s. d. 3.9) / 16.8 (s. d. 4.1)****	21.3 (s. d. 5.9) / 18.7 (s. d. 5.5)****	21.0 (s. d. 5.5) / 18.8 (s. d. 5.4)****
ENCFF015ADG	RUNX1	K562	25.4 (s. d. 6.3) / 22.8 (s. d. 6.3)****	27.1 (s. d. 7.0) / 23.3 (s. d. 6.4)****	28.9 (s. d. 7.4) / 26.7 (s. d. 7.2)****	26.5 (s. d. 7.0) / 23.1 (s. d. 6.4)****	26.1 (s. d. 6.7) / 22.8 (s. d. 6.3)****

ENCFF264QLP	RUNX1	K562	25.3 (s. d. 6.2) / 22.9 (s. d. 6.3)****	27.1 (s. d. 7.0) / 23.3 (s. d. 6.4)****	28.8 (s. d. 7.3) / 26.6 (s. d. 7.5)****	26.3 (s. d. 6.9) / 23.2 (s. d. 6.4)****	26.2 (s. d. 6.6) / 22.8 (s. d. 6.3)****
ENCFF198SPL	SPI1	GM12878	22.1 (s. d. 6.3) / 21.8 (s. d. 6.2)**	22.8 (s. d. 6.5) / 22.1 (s. d. 6.4)****	23.3 (s. d. 6.0) / 23.1 (s. d. 5.9)	22.7 (s. d. 6.6) / 22.0 (s. d. 6.3)****	22.0 (s. d. 6.4) / 21.8 (s. d. 6.1)**
ENCFF881ARS	SPI1	GM12878	22.0 (s. d. 6.4) / 21.8 (s. d. 6.2)	22.3 (s. d. 6.5) / 22.2 (s. d. 6.4)	23.4 (s. d. 6.1) / 23.3 (s. d. 5.9)	22.4 (s. d. 6.4) / 22.1 (s. d. 6.3)****	22.3 (s. d. 6.4) / 21.7 (s. d. 6.2)****
ENCFF185DGL	SPI1	K562	25.1 (s. d. 6.4) / 22.1 (s. d. 6.2)****	26.1 (s. d. 6.9) / 22.3 (s. d. 6.2)****	25.6 (s. d. 6.2) / 22.7 (s. d. 5.4)****	26.1 (s. d. 6.9) / 22.2 (s. d. 6.2)****	25.0 (s. d. 6.4) / 22.2 (s. d. 6.2)****
ENCFF456BUE	SPI1	K562	24.8 (s. d. 6.4) / 22.2 (s. d. 6.2)****	26.1 (s. d. 6.9) / 22.4 (s. d. 6.2)****	25.6 (s. d. 6.2) / 22.6 (s. d. 5.6)****	25.9 (s. d. 6.9) / 22.3 (s. d. 6.2)****	24.7 (s. d. 6.3) / 22.2 (s. d. 6.2)****
ENCFF488WQN	TP53	A549	23.6 (s. d. 6.4) / 23.6 (s. d. 6.5)	25.0 (s. d. 7.0) / 25.0 (s. d. 7.1)	28.5 (s. d. 6.8) / 28.6 (s. d. 6.9)	24.6 (s. d. 7.1) / 24.6 (s. d. 7.0)	23.6 (s. d. 6.5) / 23.4 (s. d. 6.5)
ENCFF701DTE	TP53	HepG2	23.0 (s. d. 6.4) / 23.0 (s. d. 6.5)	24.3 (s. d. 7.0) / 24.4 (s. d. 6.9)	28.9 (s. d. 6.6) / 28.5 (s. d. 6.8)****	23.8 (s. d. 6.7) / 24.0 (s. d. 6.8)	23.1 (s. d. 6.4) / 23.0 (s. d. 6.4)
ENCFF280RDQ	TP53	WTC11	23.0 (s. d. 6.7) / 23.3 (s. d. 6.5)	25.7 (s. d. 7.1) / 25.5 (s. d. 7.1)	33.2 (s. d. 5.9) / 33.1 (s. d. 6.0)	25.2 (s. d. 7.0) / 25.0 (s. d. 6.9)	23.2 (s. d. 6.3) / 23.3 (s. d. 6.5)

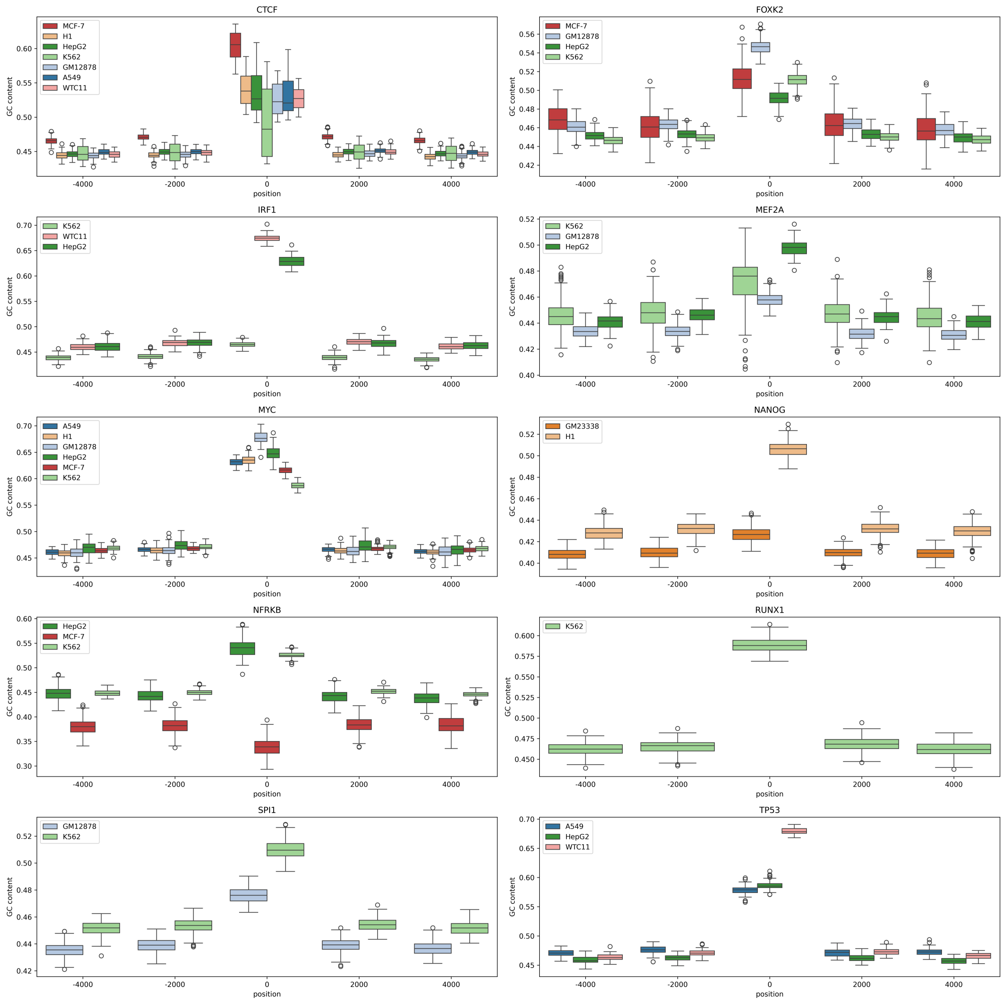

Fig. S13. GC content in the vicinity of the position (± 50bp) on the x-axis per TF and cell line. The GC content is calculated per offset from the putative binding site (experimental peak center). Positions were chosen arbitrarily.

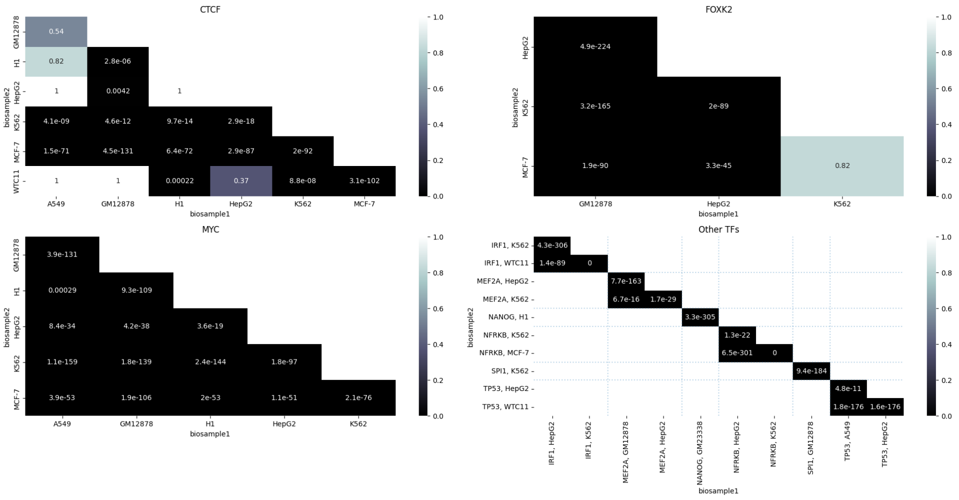

Fig. S14. Results of the statistical testing for GC content difference between cell lines in the vicinity of the binding site (±50 bp, as shown in S13 at position 0). The values represent p-values from Mann–Whitney U tests comparing each pair of cell lines, adjusted for multiple testing using the Holm correction.

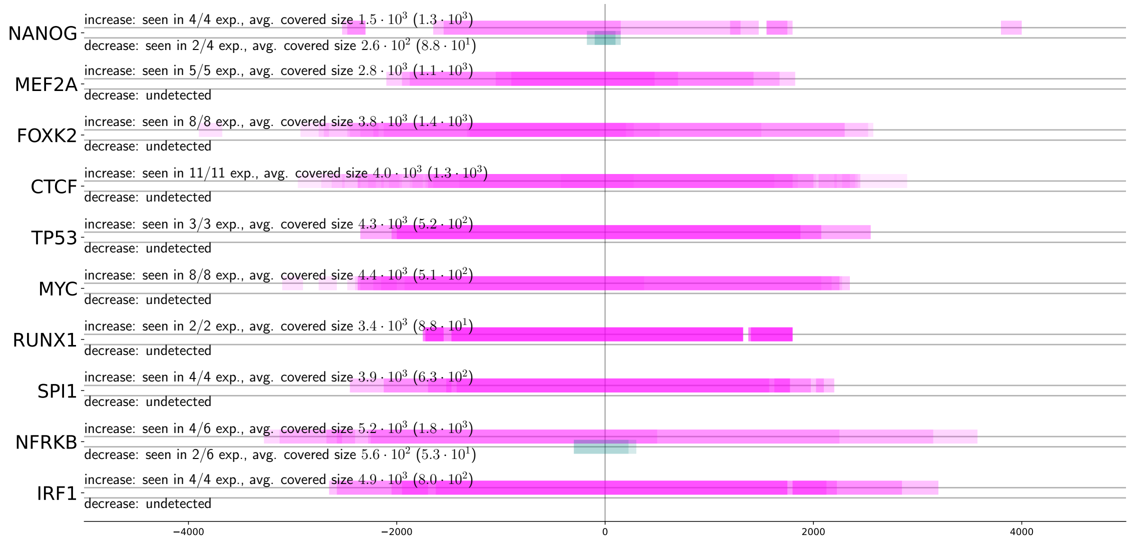

Fig. S15. Areas with significant increase or decrease in frequency of CG dinucleotide w. r. to the putative binding site position (middle of the experimental peak) for the tested TFs, using the 50 % most relevant experimental peaks. Decrease is depicted in teal, increase in magenta. Color intensity is proportional to the number of experiments corresponding to the TF – if the color if fully opaque, the excess (increase or decrease) was seen in all experiments for the given TF. The average size of covered area is calculcated as the mean area covered by excessive patches in the given excess direction. If there are no excessive patches found for a direction and experiment, it is not included in the average.

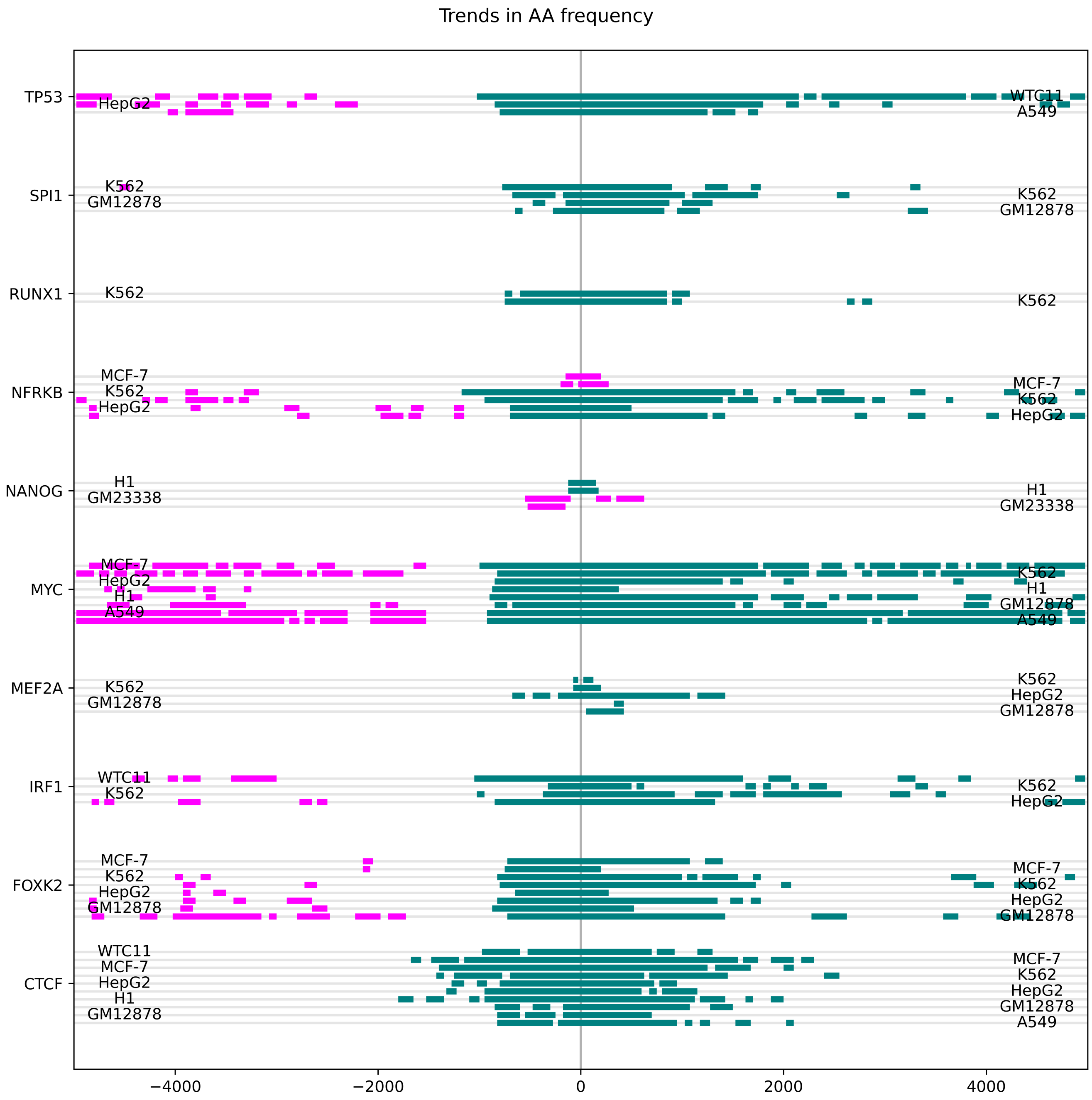

Fig. S16. Areas with significant increase or decrease (excessive patches) in frequency of the AA dinucleotide w. r. to the putative binding site position (middle of the experimental peak) for the tested TFs, using the 50 % most relevant experimental peaks. Decrease is depicted in teal, increase in magenta. The excessive patches are estimated using the permutation test.

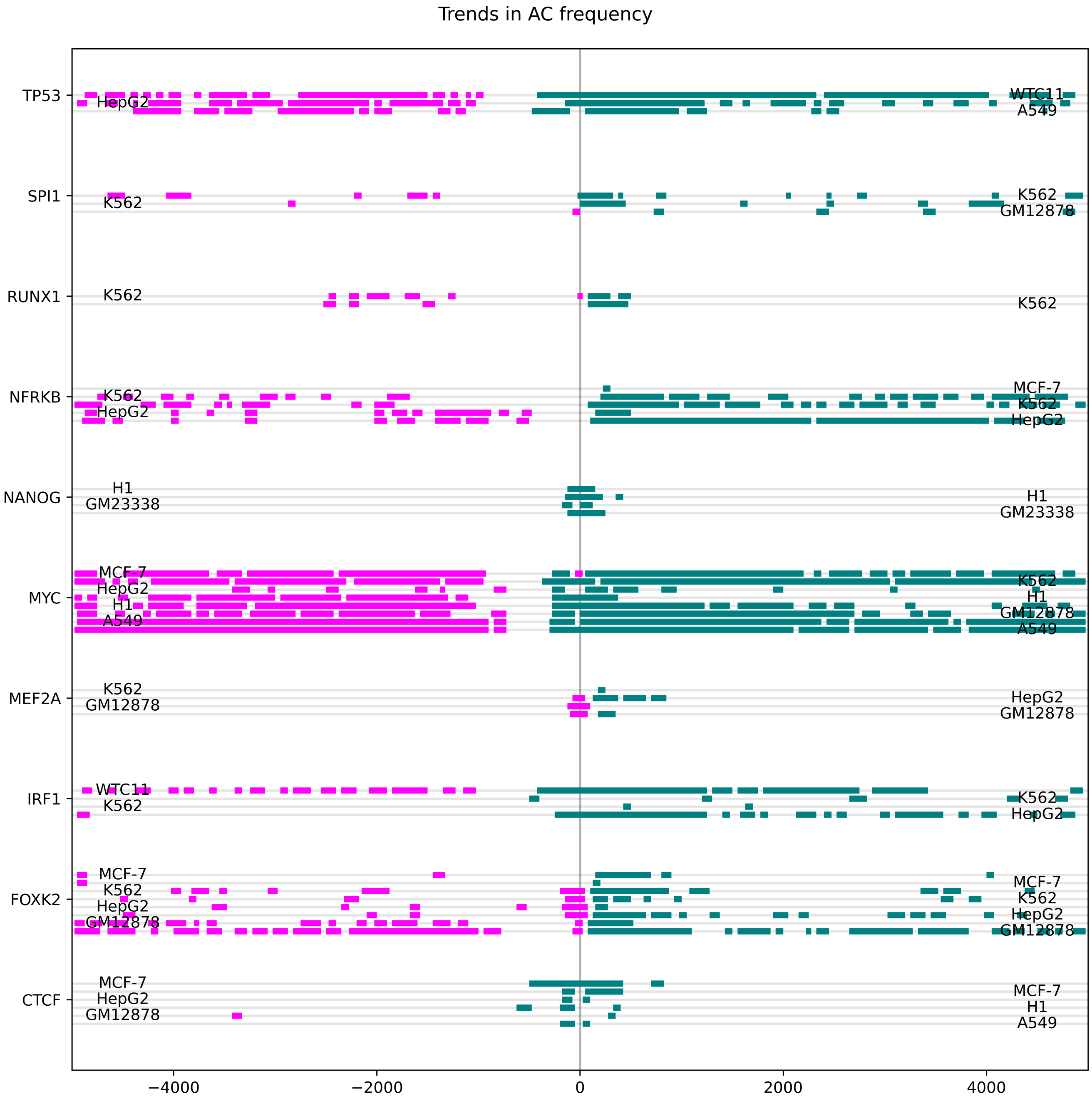

Fig. S17. Areas with significant increase or decrease (excessive patches) in frequency of the AC dinucleotide w. r. to the putative binding site position (middle of the experimental peak) for the tested TFs, using the 50 % most relevant experimental peaks. Decrease is depicted in teal, increase in magenta. The excessive patches are estimated using the permutation test.

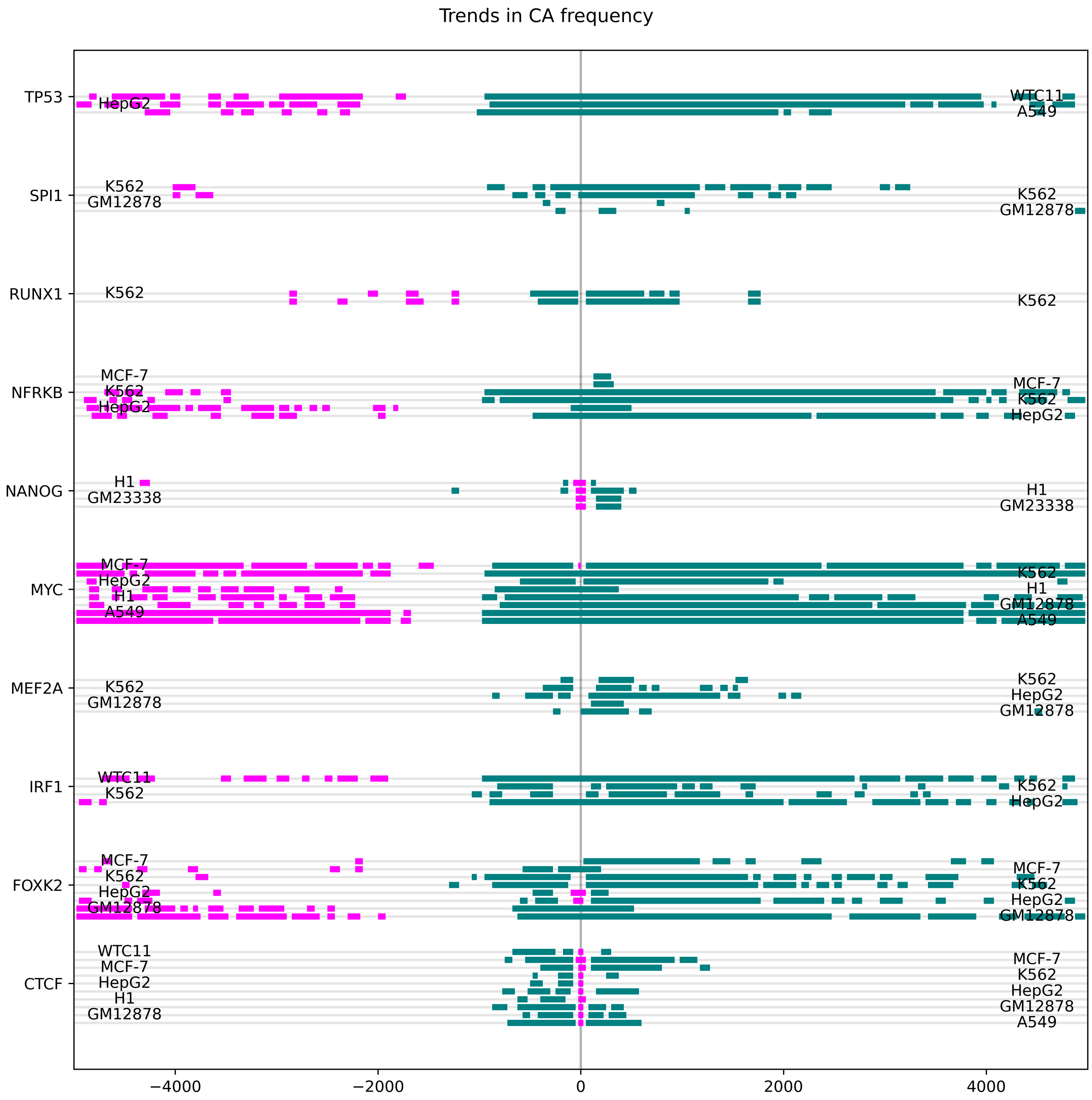

Fig. S18. Areas with significant increase or decrease (excessive patches) in frequency of the CA dinucleotide w. r. to the putative binding site position (middle of the experimental peak) for the tested TFs, using the 50 % most relevant experimental peaks. Decrease is depicted in teal, increase in magenta. The excessive patches are estimated using the permutation test.

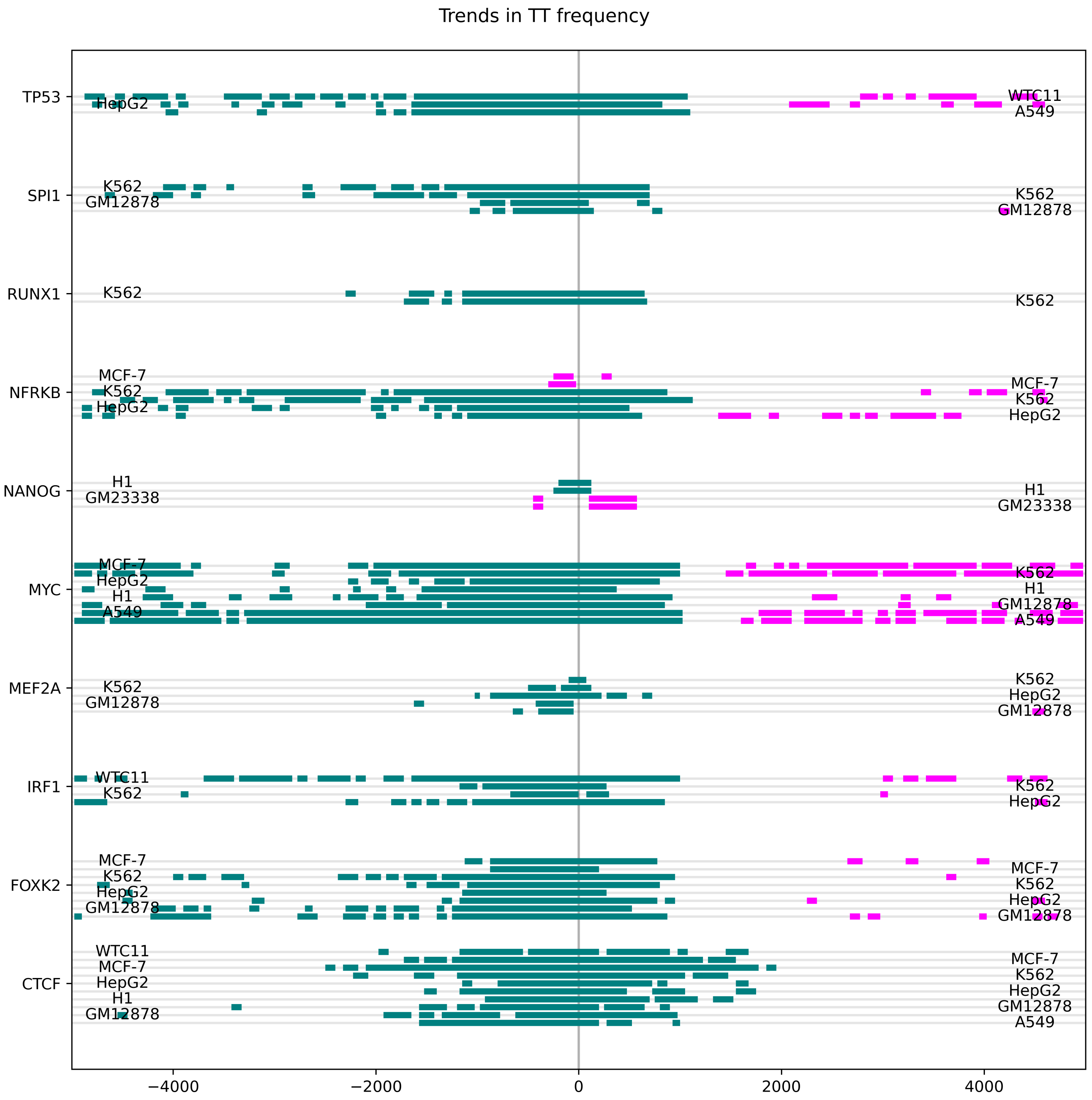

Fig. S19. Areas with significant increase or decrease (excessive patches) in frequency of the TT dinucleotide w. r. to the putative binding site position (middle of the experimental peak) for the tested TFs, using the 50 % most relevant experimental peaks. Decrease is depicted in teal, increase in magenta. The excessive patches are estimated using the permutation test.

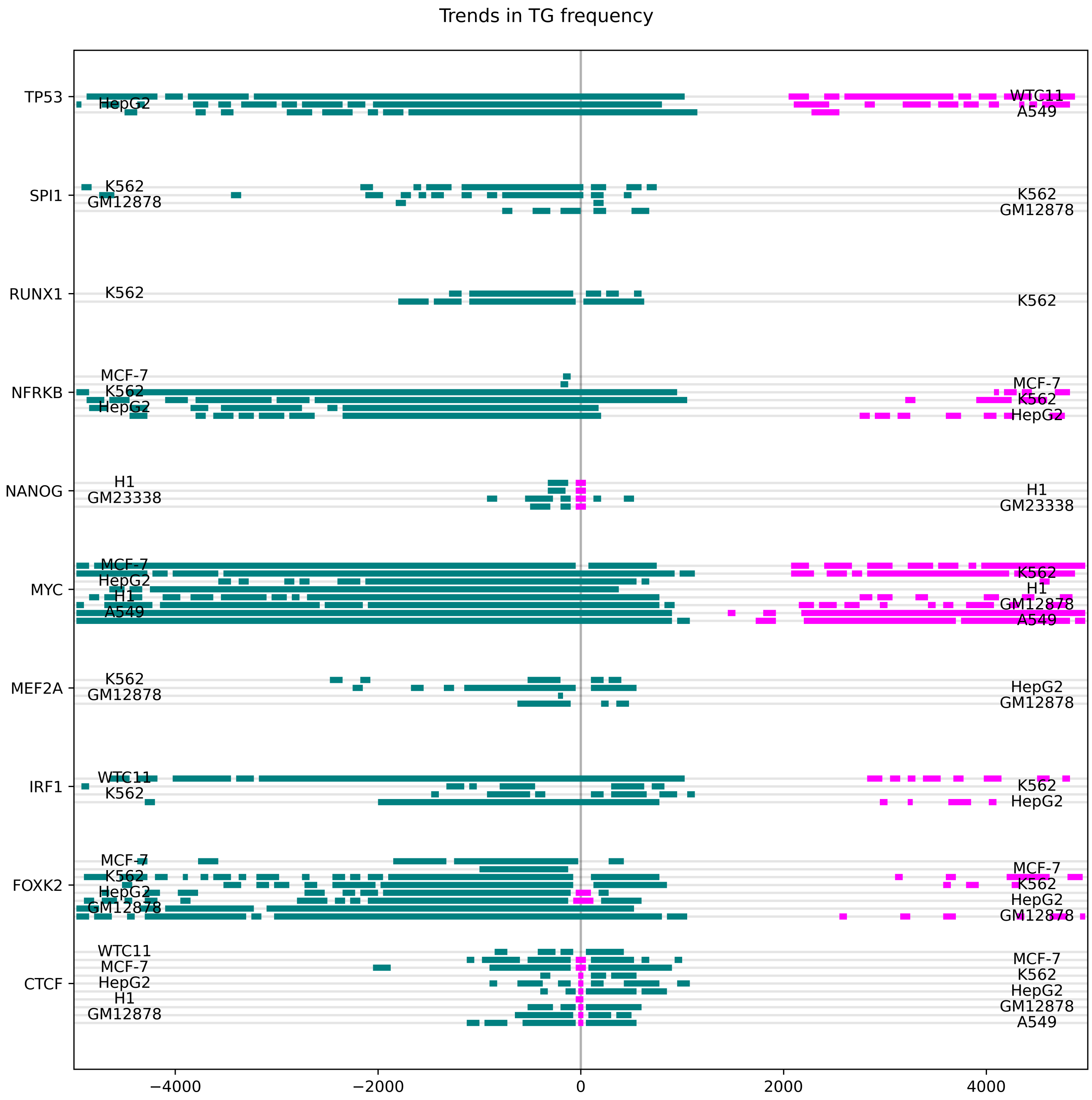

Fig. S20. Areas with significant increase or decrease (excessive patches) in frequency of the TG dinucleotide w. r. to the putative binding site position (middle of the experimental peak) for the tested TFs, using the 50 % most relevant experimental peaks. Decrease is depicted in teal, increase in magenta. The excessive patches are estimated using the permutation test.

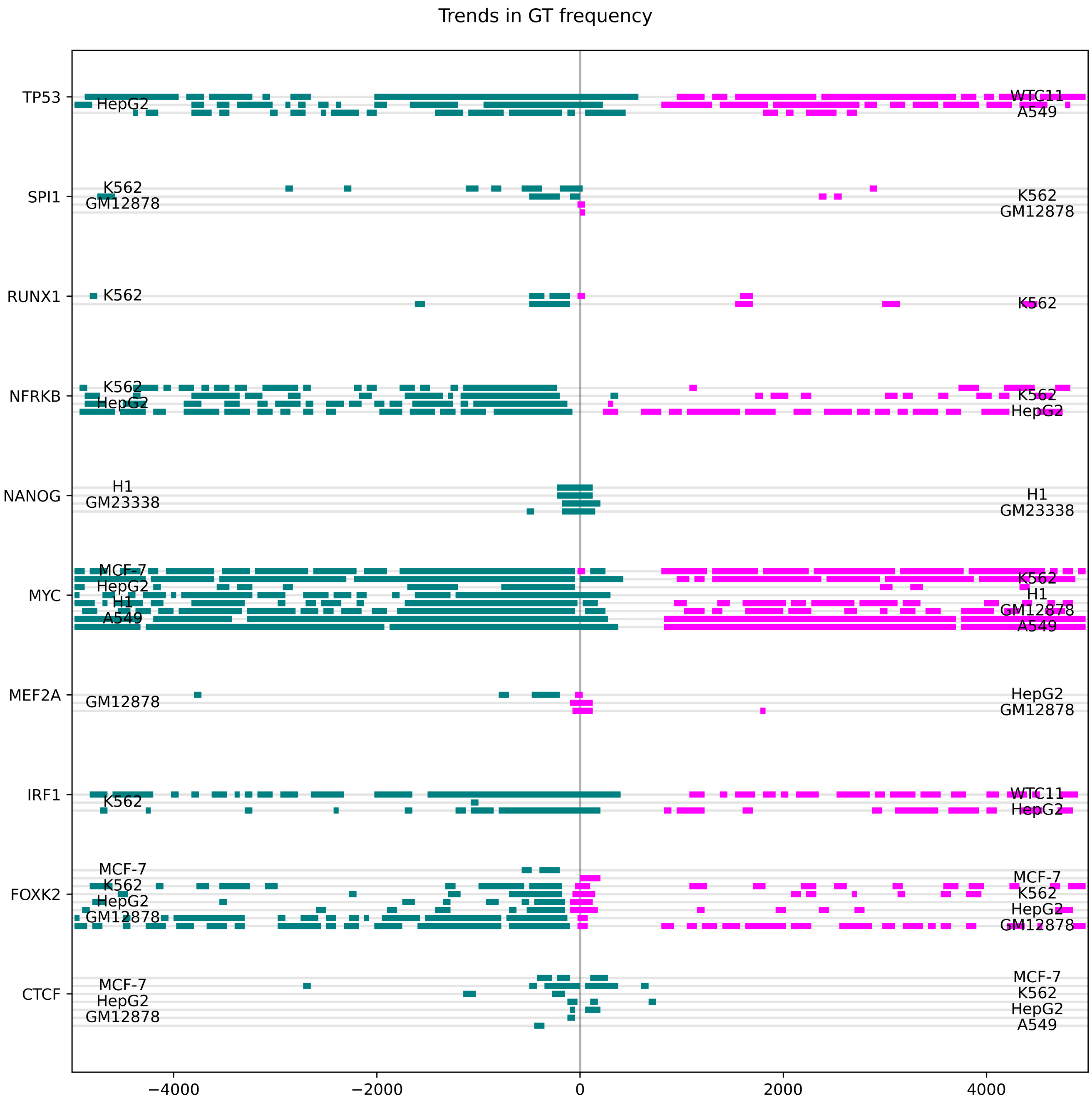

Fig. S21. Areas with significant increase or decrease (excessive patches) in frequency of the GT dinucleotide w. r. to the putative binding site position (middle of the experimental peak) for the tested TFs, using the 50 % most relevant experimental peaks. Decrease is depicted in teal, increase in magenta. The excessive patches are estimated using the permutation test.

Offset	-3000	-1500	0	1500	3000
AA	1.1 ± 2.5	1.0 ± 2.3*	-0.0 ± 1.6****	0.7 ± 2.2****	0.7 ± 2.2
ANA	1.1 ± 2.5	1.0 ± 2.3***	-0.0 ± 1.6****	0.7 ± 2.2****	0.7 ± 2.2
ANNA	0.9 ± 2.2	0.8 ± 2.1***	-0.2 ± 1.4****	0.5 ± 2.0****	0.5 ± 2.0
ANNNA	0.9 ± 2.3	0.7 ± 2.1***	-0.2 ± 1.5****	0.5 ± 2.0****	0.5 ± 2.0
ANNNNA	0.8 ± 2.1	0.7 ± 2.0***	-0.3 ± 1.4****	0.4 ± 1.9****	0.4 ± 1.9
ANNNNNA	0.8 ± 2.2	0.7 ± 2.0****	-0.2 ± 1.4****	0.4 ± 1.9****	0.4 ± 1.9
AC	0.1 ± 1.1	0.1 ± 1.1	-0.1 ± 1.1****	-0.2 ± 1.0****	-0.2 ± 1.0
ANC	0.2 ± 1.1	0.2 ± 1.1	0.1 ± 1.0****	-0.1 ± 1.0****	-0.1 ± 1.0
ANNC	0.3 ± 1.1	0.3 ± 1.1	0.1 ± 1.1****	0.0 ± 1.0****	0.0 ± 1.0
ANNNC	0.2 ± 1.1	0.2 ± 1.1	0.1 ± 1.0****	-0.0 ± 1.0****	-0.0 ± 1.0
ANNNNC	0.1 ± 1.1	0.2 ± 1.0	0.1 ± 1.0****	-0.1 ± 1.0****	-0.1 ± 1.0
ANNNNNC	-0.0 ± 1.0	0.0 ± 1.0**	-0.1 ± 1.0****	-0.2 ± 0.9****	-0.2 ± 1.0
CA	0.8 ± 1.2	0.8 ± 1.2***	0.5 ± 1.2****	0.5 ± 1.1	0.5 ± 1.1**
CNA	0.1 ± 1.1	0.1 ± 1.0	-0.0 ± 1.0****	-0.1 ± 1.0****	-0.1 ± 1.0
CNNA	0.2 ± 1.1	0.2 ± 1.1	0.1 ± 1.1****	-0.1 ± 1.0****	-0.1 ± 1.0
CNNNA	0.2 ± 1.0	0.2 ± 1.1	0.1 ± 1.0****	-0.1 ± 1.0****	-0.1 ± 1.0
CNNNNA	0.1 ± 1.0	0.1 ± 1.0	0.0 ± 1.0****	-0.1 ± 1.0****	-0.1 ± 1.0
CNNNNNA	0.0 ± 1.0	0.1 ± 1.0	-0.1 ± 1.0****	-0.2 ± 0.9****	-0.2 ± 0.9
TT	0.9 ± 2.3	0.8 ± 2.2*	-0.1 ± 1.5****	0.9 ± 2.3****	0.9 ± 2.4*
TNT	0.9 ± 2.3	0.8 ± 2.2**	-0.1 ± 1.5****	0.9 ± 2.3****	1.0 ± 2.4*
TNNT	0.7 ± 2.1	0.6 ± 2.0***	-0.2 ± 1.4****	0.6 ± 2.1****	0.7 ± 2.2**
TNNNT	0.7 ± 2.1	0.6 ± 2.0**	-0.2 ± 1.4****	0.7 ± 2.1****	0.7 ± 2.3*
TNNNNT	0.6 ± 2.0	0.5 ± 1.9**	-0.3 ± 1.3****	0.6 ± 2.0****	0.6 ± 2.1*
TNNNNNT	0.6 ± 2.0	0.5 ± 1.9**	-0.2 ± 1.4****	0.6 ± 2.0****	0.6 ± 2.1
GT	-0.1 ± 1.0	-0.1 ± 1.0	-0.1 ± 1.0	-0.1 ± 1.0	-0.1 ± 1.0
GNT	0.1 ± 1.0	0.0 ± 1.0	0.1 ± 1.0	0.0 ± 1.0	0.0 ± 1.0
GNNT	0.1 ± 1.1	0.1 ± 1.0	0.2 ± 1.1	0.2 ± 1.1	0.1 ± 1.0**
GNNNT	0.1 ± 1.0	0.1 ± 1.0	0.1 ± 1.0	0.1 ± 1.0	0.1 ± 1.0
GNNNNT	0.0 ± 1.0	0.0 ± 1.0	0.1 ± 1.0*	0.0 ± 1.0**	-0.0 ± 1.0
GNNNNNT	-0.1 ± 1.0	-0.1 ± 1.0	-0.1 ± 1.0*	-0.1 ± 1.0	-0.1 ± 1.0
TG	0.7 ± 1.2	0.6 ± 1.1***	0.5 ± 1.2****	0.6 ± 1.2****	0.7 ± 1.2*
TNG	0.0 ± 1.0	-0.0 ± 1.0	-0.0 ± 1.0	-0.0 ± 1.0	0.0 ± 1.0
TNNG	0.1 ± 1.0	0.1 ± 1.0	0.1 ± 1.1	0.1 ± 1.0	0.1 ± 1.1
TNNNG	0.0 ± 1.0	0.1 ± 1.0	0.0 ± 1.0	0.0 ± 1.0	0.0 ± 1.0
TNNNNG	-0.0 ± 1.0	-0.0 ± 1.0	0.0 ± 1.0	-0.0 ± 1.0*	-0.0 ± 1.0
TNNNNNG	-0.1 ± 1.0	-0.1 ± 1.0	-0.1 ± 1.0	-0.1 ± 1.0	-0.1 ± 1.0

Table S5. Average dinucleotide count z-score in a 50 bp k-mer centered at the specified offset for experiment ENCFF043WTJ, a ChIP-seq experiment targetting MYC in K562. Stars indicate whether the average count is significantly different from the preceding offset in this table (Welch t-test, 0.0001, indicated by "****", 0.001 by "***", 0.01 by "**" and 0.05 by"*"). Addendum to Figure 3.2. As background distribution for z-score normalization was acquired by scrambling available k-mer sequences at a given offset and identifying dinucleotide frequencies in these scrambled sequences. The process mirrors calculating scramble-normalized TF affinity (see Methods) to keep base pair frequencies but not their order.

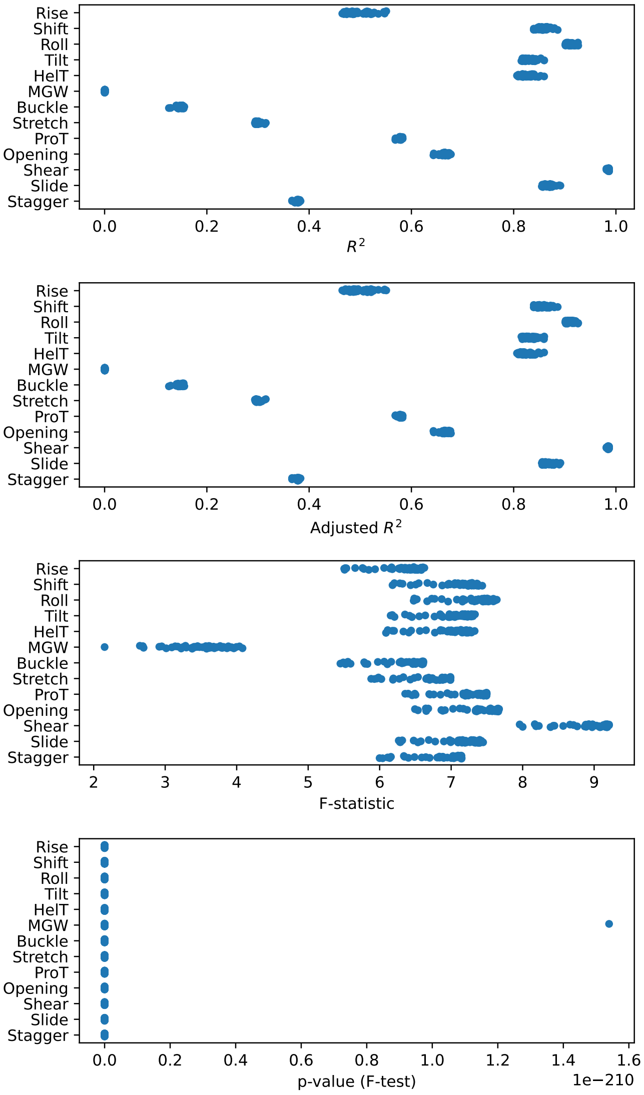

Fig. S22. Coefficients of determination and model statistics for all trained linear models by shape feature. Panels show (from top to bottom): the coefficient of determination (R^2), adjusted coefficient of determination (adjusted R^2), F-statistic values, and the corresponding p-values of the F-statistic for each model.

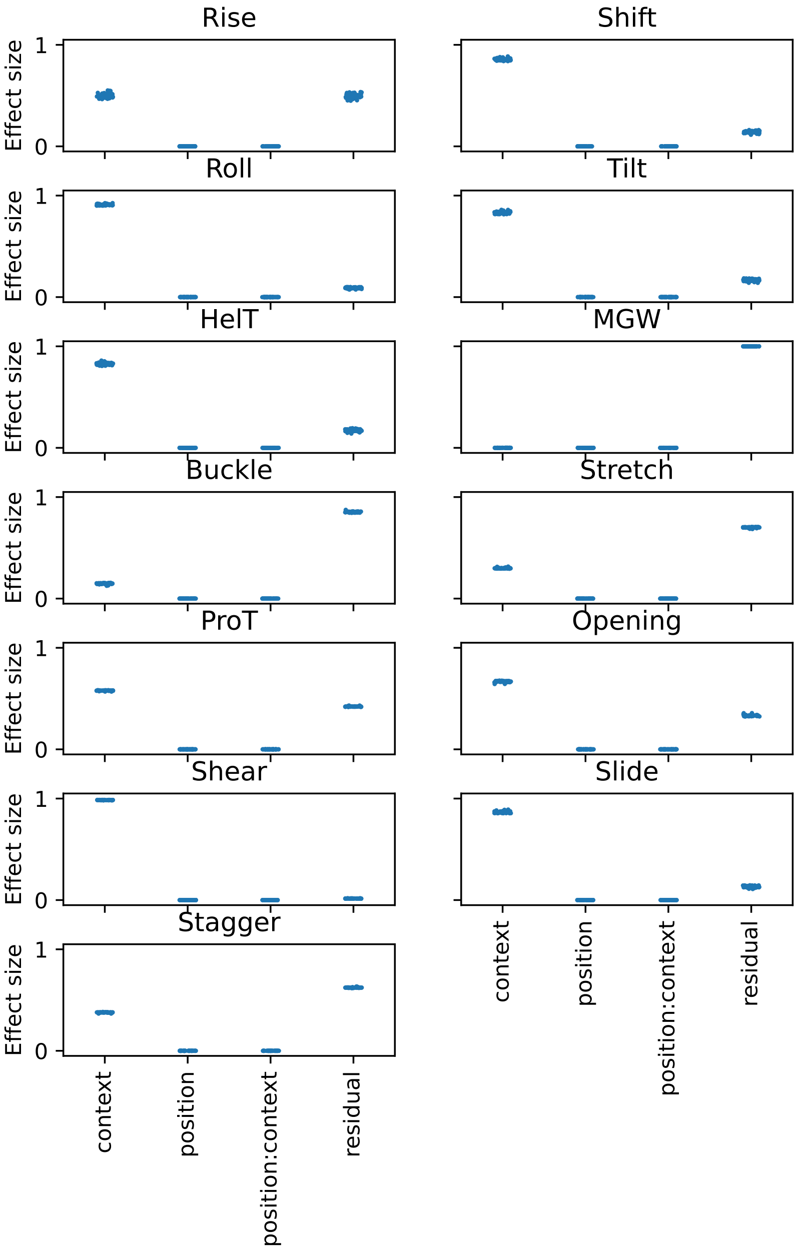

Fig. S23. Effect size of different variables. One point corresponds to one experiment. Each shape feature was modeled as a function of sequence context (defined as either a single base pair or a pair of neighboring base pairs), position relative to the binding site, and their interaction (position:context on the plot). ANOVA tables were generated using the *anova_lm* function from the *statsmodels* package, and effect size (η^2) was calculated as the ratio of the variable sum of squares to the total sum of squares.

		-3000 bp	-1500 bp	at binding site	1500 bp	3000 bp
CTCF	A	-9.51 ± 2.38	-9.51 ± 2.37	-8.44 ± 2.07	-9.49 ± 2.38	-9.52 ± 2.39
	C	-4.11 ± 2.21	-4.07 ± 2.21	-3.72 ± 2.02	-4.09 ± 2.21	-4.11 ± 2.22
	G	-4.1 ± 2.22	-4.07 ± 2.19	-3.7 ± 2.0	-4.09 ± 2.21	-4.08 ± 2.22
	T	-9.51 ± 2.38	-9.49 ± 2.38	-8.46 ± 2.08	-9.49 ± 2.37	-9.52 ± 2.38
FOXK2	A	-9.54 ± 2.43	-9.52 ± 2.42	-9.18 ± 2.21	-9.47 ± 2.38	-9.51 ± 2.41
	C	-4.1 ± 2.21	-4.11 ± 2.23	-4.22 ± 2.28	-4.1 ± 2.2	-4.12 ± 2.2
	G	-4.1 ± 2.16	-4.05 ± 2.18	-4.26 ± 2.26	-4.09 ± 2.2	-4.13 ± 2.21
	T	-9.53 ± 2.38	-9.51 ± 2.39	-9.22 ± 2.23	-9.56 ± 2.41	-9.56 ± 2.44
IRF1	A	-9.59 ± 2.41	-9.55 ± 2.37	-9.3 ± 2.32	-9.53 ± 2.38	-9.47 ± 2.37
	C	-4.16 ± 2.2	-4.03 ± 2.18	-3.94 ± 2.09	-4.09 ± 2.21	-4.09 ± 2.16
	G	-4.05 ± 2.2	-4.04 ± 2.17	-3.93 ± 2.07	-4.16 ± 2.2	-4.16 ± 2.26
	T	-9.58 ± 2.37	-9.56 ± 2.41	-9.19 ± 2.3	-9.59 ± 2.41	-9.58 ± 2.44
MEF2A	A	-9.57 ± 2.38	-9.57 ± 2.41	-9.5 ± 2.33	-9.61 ± 2.42	-9.63 ± 2.4
	C	-4.16 ± 2.2	-4.11 ± 2.18	-4.2 ± 2.19	-4.12 ± 2.21	-4.16 ± 2.23
	G	-4.11 ± 2.2	-4.12 ± 2.22	-4.15 ± 2.18	-4.19 ± 2.24	-4.16 ± 2.19
	T	-9.59 ± 2.38	-9.56 ± 2.4	-9.55 ± 2.35	-9.61 ± 2.39	-9.6 ± 2.38
MYC	A	-9.56 ± 2.44	-9.46 ± 2.43	-8.61 ± 2.08	-9.43 ± 2.37	-9.44 ± 2.42
	C	-4.1 ± 2.24	-4.06 ± 2.18	-3.78 ± 2.0	-4.01 ± 2.15	-4.03 ± 2.21
	G	-4.03 ± 2.17	-3.98 ± 2.16	-3.85 ± 2.03	-4.05 ± 2.17	-4.15 ± 2.22
	T	-9.4 ± 2.39	-9.44 ± 2.41	-8.55 ± 2.11	-9.55 ± 2.46	-9.5 ± 2.42
NANOG	A	-9.74 ± 2.38	-9.68 ± 2.37	-9.49 ± 2.21	-9.68 ± 2.34	-9.69 ± 2.36
	C	-4.25 ± 2.21	-4.2 ± 2.21	-4.15 ± 2.23	-4.16 ± 2.23	-4.2 ± 2.24
	G	-4.18 ± 2.2	-4.13 ± 2.21	-4.09 ± 2.24	-4.18 ± 2.27	-4.31 ± 2.28
	T	-9.74 ± 2.39	-9.68 ± 2.37	-9.55 ± 2.2	-9.69 ± 2.37	-9.73 ± 2.36
NFRKB	A	-9.61 ± 2.44	-9.6 ± 2.43	-9.21 ± 2.3	-9.56 ± 2.38	-9.56 ± 2.39
	C	-4.19 ± 2.22	-4.23 ± 2.23	-4.02 ± 2.16	-4.1 ± 2.21	-4.09 ± 2.21
	G	-4.13 ± 2.19	-4.08 ± 2.19	-4.06 ± 2.13	-4.14 ± 2.2	-4.15 ± 2.25
	T	-9.55 ± 2.43	-9.49 ± 2.37	-9.18 ± 2.26	-9.59 ± 2.4	-9.66 ± 2.42
RUNX1	A	-9.33 ± 2.46	-9.46 ± 2.4	-8.77 ± 2.26	-9.3 ± 2.34	-9.31 ± 2.42
	C	-4.08 ± 2.13	-3.97 ± 2.1	-3.84 ± 2.05	-3.98 ± 2.19	-4.06 ± 2.21
	G	-4.03 ± 2.2	-3.98 ± 2.2	-3.88 ± 2.04	-4.07 ± 2.16	-4.12 ± 2.19
	T	-9.48 ± 2.43	-9.36 ± 2.35	-8.9 ± 2.1	-9.32 ± 2.4	-9.45 ± 2.37
SPI1	A	-9.53 ± 2.38	-9.52 ± 2.38	-9.18 ± 2.22	-9.52 ± 2.38	-9.58 ± 2.4
	C	-4.1 ± 2.21	-4.13 ± 2.21	-3.93 ± 2.08	-4.11 ± 2.22	-4.14 ± 2.24
	G	-4.16 ± 2.2	-4.13 ± 2.22	-3.92 ± 2.11	-4.19 ± 2.25	-4.14 ± 2.23
	T	-9.56 ± 2.38	-9.54 ± 2.39	-9.14 ± 2.2	-9.57 ± 2.4	-9.6 ± 2.38
TP53	A	-9.56 ± 2.45	-9.47 ± 2.43	-8.63 ± 2.16	-9.39 ± 2.37	-9.48 ± 2.42
	C	-4.06 ± 2.17	-4.05 ± 2.21	-3.74 ± 1.95	-3.98 ± 2.13	-3.99 ± 2.19
	G	-4.07 ± 2.18	-3.99 ± 2.15	-3.77 ± 1.98	-4.06 ± 2.2	-4.08 ± 2.21
	T	-9.43 ± 2.41	-9.38 ± 2.4	-8.65 ± 2.15	-9.47 ± 2.41	-9.52 ± 2.39

Table S6. Average propeller twist values from selected positions relative to the binding site (prolonged peak center). The shape feature values were acquired using deepDNAshape from 50 bp windows.

		-3000 bp	-1500 bp	at binding site	1500 bp	3000 bp
CTCF	A	5.1 ± 0.65	5.1 ± 0.65	5.28 ± 0.52	5.1 ± 0.65	5.09 ± 0.65
	C	5.11 ± 0.5	5.11 ± 0.49	5.17 ± 0.41	5.11 ± 0.5	5.11 ± 0.5
	G	5.11 ± 0.5	5.11 ± 0.5	5.17 ± 0.42	5.11 ± 0.5	5.11 ± 0.49
	T	5.1 ± 0.66	5.1 ± 0.65	5.27 ± 0.53	5.11 ± 0.66	5.11 ± 0.65
FOXK2	A	5.09 ± 0.66	5.09 ± 0.66	5.18 ± 0.58	5.1 ± 0.65	5.09 ± 0.66
	C	5.11 ± 0.5	5.11 ± 0.49	5.16 ± 0.46	5.11 ± 0.49	5.11 ± 0.5
	G	5.11 ± 0.5	5.11 ± 0.5	5.17 ± 0.47	5.11 ± 0.49	5.11 ± 0.49
	T	5.09 ± 0.66	5.08 ± 0.66	5.18 ± 0.58	5.09 ± 0.66	5.1 ± 0.66
IRF1	A	5.08 ± 0.67	5.1 ± 0.66	5.1 ± 0.62	5.09 ± 0.66	5.1 ± 0.66
	C	5.11 ± 0.5	5.11 ± 0.49	5.14 ± 0.48	5.12 ± 0.49	5.12 ± 0.5
	G	5.11 ± 0.5	5.1 ± 0.5	5.13 ± 0.47	5.11 ± 0.5	5.1 ± 0.51
	T	5.08 ± 0.66	5.09 ± 0.67	5.15 ± 0.6	5.09 ± 0.67	5.09 ± 0.67
MEF2A	A	5.1 ± 0.67	5.1 ± 0.66	5.1 ± 0.63	5.1 ± 0.67	5.08 ± 0.66
	C	5.11 ± 0.51	5.11 ± 0.5	5.14 ± 0.5	5.1 ± 0.51	5.11 ± 0.51
	G	5.11 ± 0.51	5.11 ± 0.5	5.13 ± 0.5	5.12 ± 0.51	5.12 ± 0.51
	T	5.08 ± 0.67	5.08 ± 0.66	5.1 ± 0.64	5.08 ± 0.67	5.09 ± 0.66
MYC	A	5.09 ± 0.66	5.1 ± 0.65	5.23 ± 0.54	5.09 ± 0.66	5.1 ± 0.66
	C	5.12 ± 0.49	5.12 ± 0.49	5.19 ± 0.41	5.12 ± 0.48	5.11 ± 0.5
	G	5.12 ± 0.5	5.12 ± 0.48	5.2 ± 0.41	5.12 ± 0.49	5.11 ± 0.5
	T	5.11 ± 0.65	5.09 ± 0.65	5.24 ± 0.54	5.09 ± 0.67	5.11 ± 0.65
NANOG	A	5.07 ± 0.67	5.09 ± 0.67	5.13 ± 0.6	5.1 ± 0.66	5.09 ± 0.67
	C	5.11 ± 0.52	5.1 ± 0.52	5.13 ± 0.51	5.1 ± 0.51	5.09 ± 0.52
	G	5.11 ± 0.52	5.09 ± 0.51	5.13 ± 0.5	5.1 ± 0.52	5.1 ± 0.54
	T	5.07 ± 0.68	5.08 ± 0.68	5.13 ± 0.6	5.07 ± 0.67	5.08 ± 0.67
NFRKB	A	5.08 ± 0.66	5.09 ± 0.66	5.13 ± 0.6	5.09 ± 0.66	5.08 ± 0.66
	C	5.1 ± 0.5	5.12 ± 0.5	5.14 ± 0.46	5.11 ± 0.5	5.11 ± 0.5
	G	5.1 ± 0.5	5.11 ± 0.5	5.13 ± 0.47	5.12 ± 0.51	5.11 ± 0.51
	T	5.09 ± 0.66	5.09 ± 0.66	5.14 ± 0.61	5.09 ± 0.67	5.08 ± 0.67
RUNX1	A	5.09 ± 0.65	5.13 ± 0.65	5.25 ± 0.56	5.15 ± 0.62	5.11 ± 0.65
	C	5.12 ± 0.52	5.1 ± 0.49	5.17 ± 0.45	5.11 ± 0.48	5.11 ± 0.49
	G	5.11 ± 0.49	5.1 ± 0.48	5.17 ± 0.45	5.11 ± 0.49	5.16 ± 0.48
	T	5.1 ± 0.66	5.14 ± 0.65	5.25 ± 0.53	5.11 ± 0.64	5.12 ± 0.63
SPI1	A	5.09 ± 0.66	5.1 ± 0.66	5.07 ± 0.58	5.11 ± 0.66	5.08 ± 0.66
	C	5.12 ± 0.5	5.11 ± 0.5	5.09 ± 0.48	5.11 ± 0.5	5.11 ± 0.5
	G	5.11 ± 0.51	5.11 ± 0.5	5.09 ± 0.48	5.11 ± 0.51	5.11 ± 0.5
	T	5.09 ± 0.67	5.1 ± 0.66	5.07 ± 0.58	5.1 ± 0.66	5.1 ± 0.66
TP53	A	5.09 ± 0.67	5.1 ± 0.65	5.21 ± 0.55	5.11 ± 0.64	5.09 ± 0.65
	C	5.12 ± 0.49	5.11 ± 0.49	5.18 ± 0.43	5.11 ± 0.49	5.12 ± 0.49
	G	5.12 ± 0.49	5.12 ± 0.49	5.18 ± 0.42	5.11 ± 0.48	5.12 ± 0.5
	T	5.11 ± 0.65	5.1 ± 0.66	5.21 ± 0.55	5.1 ± 0.65	5.09 ± 0.67

Table S7. Average minor groove width values from selected positions relative to the binding site (prolonged peak center). The shape feature values were acquired using deepDNAshape from 50 bp windows.

		-3000 bp	-1500 bp	at binding site	1500 bp	3000 bp
CTCF	A	-0.88 ± 0.55	-0.88 ± 0.55	-0.73 ± 0.46	-0.87 ± 0.55	-0.88 ± 0.55
	C	0.31 ± 0.14	0.31 ± 0.14	0.3 ± 0.13	0.31 ± 0.14	0.31 ± 0.14
	G	0.31 ± 0.15	0.31 ± 0.14	0.31 ± 0.13	0.31 ± 0.15	0.31 ± 0.14
	T	-0.87 ± 0.55	-0.87 ± 0.55	-0.74 ± 0.46	-0.88 ± 0.55	-0.88 ± 0.55
FOXK2	A	-0.89 ± 0.56	-0.88 ± 0.56	-0.85 ± 0.52	-0.87 ± 0.56	-0.88 ± 0.55
	C	0.31 ± 0.15	0.31 ± 0.15	0.32 ± 0.14	0.31 ± 0.14	0.31 ± 0.15
	G	0.31 ± 0.14	0.31 ± 0.15	0.32 ± 0.15	0.31 ± 0.15	0.31 ± 0.14
	T	-0.88 ± 0.56	-0.88 ± 0.56	-0.85 ± 0.52	-0.89 ± 0.56	-0.9 ± 0.56
IRF1	A	-0.89 ± 0.56	-0.89 ± 0.55	-0.84 ± 0.53	-0.88 ± 0.56	-0.87 ± 0.55
	C	0.31 ± 0.14	0.31 ± 0.15	0.31 ± 0.14	0.31 ± 0.15	0.31 ± 0.14
	G	0.31 ± 0.15	0.31 ± 0.14	0.31 ± 0.14	0.31 ± 0.15	0.31 ± 0.15
	T	-0.88 ± 0.56	-0.89 ± 0.56	-0.84 ± 0.53	-0.89 ± 0.57	-0.89 ± 0.56
MEF2A	A	-0.89 ± 0.56	-0.9 ± 0.56	-0.88 ± 0.54	-0.9 ± 0.56	-0.9 ± 0.56
	C	0.31 ± 0.14	0.31 ± 0.14	0.31 ± 0.15	0.31 ± 0.14	0.31 ± 0.15
	G	0.31 ± 0.14	0.31 ± 0.15	0.31 ± 0.15	0.31 ± 0.15	0.31 ± 0.15
	T	-0.89 ± 0.55	-0.88 ± 0.57	-0.89 ± 0.54	-0.89 ± 0.56	-0.89 ± 0.55
MYC	A	-0.9 ± 0.57	-0.88 ± 0.56	-0.76 ± 0.47	-0.86 ± 0.55	-0.86 ± 0.56
	C	0.31 ± 0.14	0.31 ± 0.14	0.32 ± 0.13	0.31 ± 0.14	0.31 ± 0.14
	G	0.31 ± 0.14	0.31 ± 0.14	0.32 ± 0.13	0.31 ± 0.14	0.31 ± 0.15
	T	-0.87 ± 0.55	-0.86 ± 0.56	-0.75 ± 0.47	-0.9 ± 0.57	-0.89 ± 0.56
NANOG	A	-0.91 ± 0.56	-0.91 ± 0.55	-0.89 ± 0.52	-0.9 ± 0.55	-0.9 ± 0.56
	C	0.31 ± 0.15	0.31 ± 0.15	0.31 ± 0.14	0.31 ± 0.15	0.31 ± 0.14
	G	0.31 ± 0.14	0.31 ± 0.15	0.31 ± 0.14	0.31 ± 0.15	0.32 ± 0.15
	T	-0.91 ± 0.56	-0.9 ± 0.55	-0.89 ± 0.52	-0.9 ± 0.55	-0.91 ± 0.56
NFRKB	A	-0.9 ± 0.57	-0.9 ± 0.56	-0.84 ± 0.54	-0.88 ± 0.55	-0.89 ± 0.55
	C	0.32 ± 0.15	0.31 ± 0.15	0.32 ± 0.14	0.31 ± 0.15	0.31 ± 0.15
	G	0.31 ± 0.14	0.31 ± 0.15	0.32 ± 0.14	0.31 ± 0.15	0.31 ± 0.15
	T	-0.89 ± 0.56	-0.87 ± 0.55	-0.82 ± 0.53	-0.9 ± 0.56	-0.91 ± 0.57
RUNX1	A	-0.84 ± 0.56	-0.89 ± 0.57	-0.81 ± 0.5	-0.87 ± 0.55	-0.85 ± 0.55
	C	0.3 ± 0.14	0.31 ± 0.15	0.31 ± 0.13	0.31 ± 0.14	0.31 ± 0.14
	G	0.31 ± 0.14	0.31 ± 0.14	0.32 ± 0.14	0.32 ± 0.15	0.31 ± 0.15
	T	-0.88 ± 0.55	-0.88 ± 0.54	-0.83 ± 0.5	-0.85 ± 0.53	-0.88 ± 0.54
SPI1	A	-0.88 ± 0.56	-0.88 ± 0.54	-0.81 ± 0.53	-0.89 ± 0.55	-0.89 ± 0.56
	C	0.31 ± 0.14	0.31 ± 0.15	0.31 ± 0.14	0.31 ± 0.14	0.31 ± 0.15
	G	0.31 ± 0.14	0.31 ± 0.14	0.31 ± 0.13	0.32 ± 0.15	0.31 ± 0.15
	T	-0.88 ± 0.55	-0.89 ± 0.55	-0.8 ± 0.52	-0.9 ± 0.55	-0.89 ± 0.55
TP53	A	-0.89 ± 0.57	-0.88 ± 0.56	-0.75 ± 0.49	-0.87 ± 0.55	-0.87 ± 0.56
	C	0.31 ± 0.15	0.32 ± 0.15	0.32 ± 0.13	0.31 ± 0.14	0.31 ± 0.14
	G	0.31 ± 0.14	0.31 ± 0.14	0.32 ± 0.13	0.31 ± 0.14	0.31 ± 0.15
	T	-0.87 ± 0.55	-0.86 ± 0.55	-0.75 ± 0.48	-0.88 ± 0.56	-0.88 ± 0.55

Table S8. Average opening values from selected positions relative to the binding site (prolonged peak center). The shape feature values were acquired using deepDNAshape from 50 bp windows.

		-3000 bp	-1500 bp	at binding site	1500 bp	3000 bp
CTCF	A	-0.02 ± 0.01	-0.02 ± 0.01	-0.02 ± 0.01	-0.02 ± 0.01	-0.02 ± 0.01
	C	-0.03 ± 0.01	-0.03 ± 0.01	-0.03 ± 0.01	-0.03 ± 0.01	-0.03 ± 0.01
	G	-0.03 ± 0.01	-0.03 ± 0.01	-0.03 ± 0.01	-0.03 ± 0.01	-0.03 ± 0.01
	T	-0.02 ± 0.01	-0.02 ± 0.01	-0.02 ± 0.01	-0.02 ± 0.01	-0.02 ± 0.01
FOXK2	A	-0.02 ± 0.01	-0.02 ± 0.01	-0.02 ± 0.01	-0.02 ± 0.01	-0.02 ± 0.01
	C	-0.03 ± 0.01	-0.03 ± 0.01	-0.03 ± 0.01	-0.03 ± 0.01	-0.03 ± 0.01
	G	-0.03 ± 0.01	-0.03 ± 0.01	-0.03 ± 0.01	-0.03 ± 0.01	-0.03 ± 0.01
	T	-0.02 ± 0.01	-0.02 ± 0.01	-0.02 ± 0.01	-0.02 ± 0.01	-0.02 ± 0.01
IRF1	A	-0.02 ± 0.01	-0.02 ± 0.01	-0.02 ± 0.01	-0.02 ± 0.01	-0.02 ± 0.01
	C	-0.03 ± 0.01	-0.03 ± 0.01	-0.03 ± 0.01	-0.03 ± 0.01	-0.03 ± 0.01
	G	-0.03 ± 0.01	-0.03 ± 0.01	-0.03 ± 0.01	-0.03 ± 0.01	-0.03 ± 0.01
	T	-0.02 ± 0.01	-0.02 ± 0.01	-0.02 ± 0.01	-0.02 ± 0.01	-0.02 ± 0.01
MEF2A	A	-0.02 ± 0.01	-0.02 ± 0.01	-0.02 ± 0.01	-0.02 ± 0.01	-0.02 ± 0.01
	C	-0.03 ± 0.01	-0.03 ± 0.01	-0.03 ± 0.01	-0.03 ± 0.01	-0.03 ± 0.01
	G	-0.03 ± 0.01	-0.03 ± 0.01	-0.03 ± 0.01	-0.03 ± 0.01	-0.03 ± 0.01
	T	-0.02 ± 0.01	-0.02 ± 0.01	-0.02 ± 0.01	-0.02 ± 0.01	-0.02 ± 0.01
MYC	A	-0.02 ± 0.01	-0.02 ± 0.01	-0.02 ± 0.01	-0.02 ± 0.01	-0.02 ± 0.01
	C	-0.03 ± 0.01	-0.03 ± 0.01	-0.03 ± 0.01	-0.03 ± 0.01	-0.03 ± 0.01
	G	-0.03 ± 0.01	-0.03 ± 0.01	-0.03 ± 0.01	-0.03 ± 0.01	-0.03 ± 0.01
	T	-0.02 ± 0.01	-0.02 ± 0.01	-0.02 ± 0.01	-0.02 ± 0.01	-0.02 ± 0.01
NANOG	A	-0.02 ± 0.01	-0.02 ± 0.01	-0.02 ± 0.01	-0.02 ± 0.01	-0.02 ± 0.01
	C	-0.03 ± 0.01	-0.03 ± 0.01	-0.03 ± 0.01	-0.03 ± 0.01	-0.03 ± 0.01
	G	-0.03 ± 0.01	-0.03 ± 0.01	-0.03 ± 0.01	-0.03 ± 0.01	-0.03 ± 0.01
	T	-0.02 ± 0.01	-0.02 ± 0.01	-0.02 ± 0.01	-0.02 ± 0.01	-0.02 ± 0.01
NFRKB	A	-0.02 ± 0.01	-0.02 ± 0.01	-0.02 ± 0.01	-0.02 ± 0.01	-0.02 ± 0.01
	C	-0.03 ± 0.01	-0.03 ± 0.01	-0.03 ± 0.01	-0.03 ± 0.01	-0.03 ± 0.01
	G	-0.03 ± 0.01	-0.03 ± 0.01	-0.03 ± 0.01	-0.03 ± 0.01	-0.03 ± 0.01
	T	-0.02 ± 0.01	-0.02 ± 0.01	-0.02 ± 0.01	-0.02 ± 0.01	-0.02 ± 0.01
RUNX1	A	-0.02 ± 0.01	-0.02 ± 0.01	-0.02 ± 0.01	-0.02 ± 0.01	-0.02 ± 0.01
	C	-0.03 ± 0.01	-0.03 ± 0.01	-0.03 ± 0.01	-0.03 ± 0.01	-0.03 ± 0.01
	G	-0.03 ± 0.01	-0.03 ± 0.01	-0.03 ± 0.01	-0.03 ± 0.01	-0.03 ± 0.01
	T	-0.02 ± 0.01	-0.02 ± 0.01	-0.02 ± 0.01	-0.02 ± 0.01	-0.02 ± 0.01
SPI1	A	-0.02 ± 0.01	-0.02 ± 0.01	-0.02 ± 0.01	-0.02 ± 0.01	-0.02 ± 0.01
	C	-0.03 ± 0.01	-0.03 ± 0.01	-0.03 ± 0.01	-0.03 ± 0.01	-0.03 ± 0.01
	G	-0.03 ± 0.01	-0.03 ± 0.01	-0.03 ± 0.01	-0.03 ± 0.01	-0.03 ± 0.01
	T	-0.02 ± 0.01	-0.02 ± 0.01	-0.02 ± 0.01	-0.02 ± 0.01	-0.02 ± 0.01
TP53	A	-0.02 ± 0.01	-0.02 ± 0.01	-0.02 ± 0.01	-0.02 ± 0.01	-0.02 ± 0.01
	C	-0.03 ± 0.01	-0.03 ± 0.01	-0.03 ± 0.01	-0.03 ± 0.01	-0.03 ± 0.01
	G	-0.03 ± 0.01	-0.03 ± 0.01	-0.03 ± 0.01	-0.03 ± 0.01	-0.03 ± 0.01
	T	-0.02 ± 0.01	-0.02 ± 0.01	-0.02 ± 0.01	-0.02 ± 0.01	-0.02 ± 0.01

Table S9. Average stretch values from selected positions relative to the binding site (prolonged peak center). The shape feature values were acquired using deepDNAshape from 50 bp windows.

		-3000 bp	-1500 bp	at binding site	1500 bp	3000 bp
CTCF	A	-0.08 ± 0.11	-0.08 ± 0.11	-0.07 ± 0.1	-0.08 ± 0.11	-0.08 ± 0.11
	C	0.06 ± 0.07	0.06 ± 0.07	0.06 ± 0.07	0.06 ± 0.07	0.06 ± 0.07
	G	0.06 ± 0.07	0.06 ± 0.07	0.06 ± 0.07	0.06 ± 0.07	0.06 ± 0.07
	T	-0.08 ± 0.11	-0.08 ± 0.11	-0.07 ± 0.1	-0.08 ± 0.11	-0.08 ± 0.11
FOXK2	A	-0.08 ± 0.11	-0.08 ± 0.11	-0.07 ± 0.11	-0.08 ± 0.11	-0.08 ± 0.11
	C	0.06 ± 0.07	0.06 ± 0.07	0.06 ± 0.07	0.06 ± 0.07	0.06 ± 0.07
	G	0.06 ± 0.07	0.06 ± 0.07	0.06 ± 0.07	0.06 ± 0.07	0.06 ± 0.07
	T	-0.08 ± 0.11	-0.08 ± 0.11	-0.07 ± 0.11	-0.08 ± 0.11	-0.08 ± 0.11
IRF1	A	-0.08 ± 0.11	-0.08 ± 0.11	-0.08 ± 0.11	-0.08 ± 0.11	-0.08 ± 0.11
	C	0.06 ± 0.07	0.06 ± 0.07	0.06 ± 0.07	0.06 ± 0.07	0.06 ± 0.07
	G	0.06 ± 0.07	0.06 ± 0.07	0.06 ± 0.07	0.06 ± 0.07	0.06 ± 0.07
	T	-0.08 ± 0.11	-0.08 ± 0.11	-0.08 ± 0.11	-0.08 ± 0.11	-0.08 ± 0.11
MEF2A	A	-0.08 ± 0.11	-0.08 ± 0.11	-0.08 ± 0.11	-0.08 ± 0.11	-0.08 ± 0.11
	C	0.06 ± 0.07	0.06 ± 0.07	0.06 ± 0.07	0.06 ± 0.07	0.06 ± 0.07
	G	0.06 ± 0.07	0.06 ± 0.07	0.06 ± 0.07	0.06 ± 0.07	0.06 ± 0.07
	T	-0.08 ± 0.11	-0.08 ± 0.11	-0.08 ± 0.11	-0.08 ± 0.11	-0.08 ± 0.11
MYC	A	-0.08 ± 0.11	-0.08 ± 0.11	-0.07 ± 0.1	-0.08 ± 0.11	-0.08 ± 0.11
	C	0.06 ± 0.07	0.06 ± 0.07	0.06 ± 0.07	0.06 ± 0.07	0.06 ± 0.07
	G	0.06 ± 0.07	0.06 ± 0.07	0.06 ± 0.07	0.06 ± 0.07	0.06 ± 0.07
	T	-0.08 ± 0.1	-0.08 ± 0.1	-0.06 ± 0.1	-0.08 ± 0.11	-0.08 ± 0.11
NANOG	A	-0.09 ± 0.11	-0.08 ± 0.11	-0.09 ± 0.11	-0.09 ± 0.11	-0.08 ± 0.11
	C	0.06 ± 0.07	0.06 ± 0.07	0.06 ± 0.07	0.07 ± 0.07	0.06 ± 0.07
	G	0.06 ± 0.07	0.06 ± 0.07	0.06 ± 0.07	0.06 ± 0.07	0.06 ± 0.07
	T	-0.08 ± 0.11	-0.08 ± 0.11	-0.09 ± 0.11	-0.08 ± 0.11	-0.09 ± 0.11
NFRKB	A	-0.08 ± 0.11	-0.08 ± 0.11	-0.08 ± 0.11	-0.08 ± 0.11	-0.08 ± 0.11
	C	0.06 ± 0.07	0.06 ± 0.07	0.06 ± 0.07	0.06 ± 0.07	0.06 ± 0.07
	G	0.06 ± 0.07	0.06 ± 0.07	0.06 ± 0.07	0.06 ± 0.07	0.06 ± 0.07
	T	-0.08 ± 0.11	-0.08 ± 0.11	-0.08 ± 0.11	-0.08 ± 0.11	-0.08 ± 0.11
RUNX1	A	-0.08 ± 0.1	-0.08 ± 0.1	-0.07 ± 0.1	-0.08 ± 0.11	-0.08 ± 0.1
	C	0.06 ± 0.07	0.06 ± 0.07	0.06 ± 0.07	0.06 ± 0.07	0.06 ± 0.07
	G	0.06 ± 0.07	0.06 ± 0.07	0.06 ± 0.07	0.06 ± 0.07	0.06 ± 0.07
	T	-0.08 ± 0.11	-0.07 ± 0.11	-0.07 ± 0.11	-0.08 ± 0.11	-0.08 ± 0.11
SPI1	A	-0.08 ± 0.11	-0.08 ± 0.11	-0.08 ± 0.11	-0.08 ± 0.11	-0.08 ± 0.11
	C	0.06 ± 0.07	0.06 ± 0.07	0.06 ± 0.07	0.06 ± 0.07	0.06 ± 0.07
	G	0.06 ± 0.07	0.06 ± 0.07	0.06 ± 0.07	0.06 ± 0.07	0.06 ± 0.07
	T	-0.08 ± 0.11	-0.08 ± 0.11	-0.08 ± 0.11	-0.08 ± 0.11	-0.08 ± 0.11
TP53	A	-0.08 ± 0.11	-0.08 ± 0.11	-0.07 ± 0.1	-0.08 ± 0.11	-0.08 ± 0.1
	C	0.06 ± 0.07	0.06 ± 0.07	0.06 ± 0.07	0.06 ± 0.07	0.06 ± 0.07
	G	0.06 ± 0.07	0.06 ± 0.07	0.06 ± 0.07	0.06 ± 0.07	0.06 ± 0.07
	T	-0.08 ± 0.11	-0.08 ± 0.1	-0.07 ± 0.1	-0.08 ± 0.11	-0.08 ± 0.11

Table S10. Average stagger values from selected positions relative to the binding site (prolonged peak center). The shape feature values were acquired using deepDNAshape from 50 bp windows.

Table S11. Average roll values from selected positions relative to the binding site (prolonged peak center). The shape feature values were acquired using deepDNAshape from 50 bp windows.

		-3000 bp	-1500 bp	at binding site	1500 bp	3000 bp
CTCF	AA	-3.31 ± 1.07	-3.31 ± 1.07	-3.24 ± 0.92	-3.3 ± 1.04	-3.32 ± 1.06
	AC	-3.0 ± 0.94	-3.01 ± 0.94	-2.84 ± 0.75	-3.0 ± 0.97	-3.01 ± 0.96
	AG	-2.39 ± 0.75	-2.42 ± 0.77	-2.22 ± 0.71	-2.39 ± 0.77	-2.39 ± 0.77
	AT	-4.67 ± 1.17	-4.67 ± 1.19	-4.54 ± 1.01	-4.69 ± 1.2	-4.68 ± 1.22
	CA	4.06 ± 1.2	4.05 ± 1.19	3.72 ± 1.08	4.05 ± 1.2	4.07 ± 1.18
	CC	-1.63 ± 0.7	-1.63 ± 0.7	-1.63 ± 0.69	-1.62 ± 0.7	-1.63 ± 0.7
	CG	3.42 ± 1.27	3.31 ± 1.24	3.0 ± 1.1	3.31 ± 1.26	3.4 ± 1.29
	CT	-2.4 ± 0.77	-2.39 ± 0.76	-2.23 ± 0.71	-2.4 ± 0.77	-2.39 ± 0.76
	GA	-1.11 ± 0.8	-1.11 ± 0.77	-1.15 ± 0.71	-1.1 ± 0.78	-1.11 ± 0.77
	GC	-2.17 ± 0.9	-2.19 ± 0.9	-1.92 ± 0.74	-2.16 ± 0.9	-2.16 ± 0.88
	GG	-1.64 ± 0.7	-1.63 ± 0.69	-1.64 ± 0.68	-1.64 ± 0.7	-1.62 ± 0.7
	GT	-3.01 ± 0.98	-3.01 ± 0.96	-2.86 ± 0.78	-3.01 ± 0.96	-3.01 ± 0.97
	TA	5.39 ± 1.27	5.43 ± 1.28	5.09 ± 1.1	5.42 ± 1.28	5.44 ± 1.31
	TC	-1.13 ± 0.79	-1.12 ± 0.78	-1.14 ± 0.71	-1.09 ± 0.79	-1.1 ± 0.78
	TG	4.07 ± 1.19	4.04 ± 1.2	3.69 ± 1.07	4.06 ± 1.19	4.07 ± 1.19
	TT	-3.32 ± 1.06	-3.31 ± 1.06	-3.25 ± 0.94	-3.33 ± 1.05	-3.32 ± 1.07
FOXK2	AA	-3.31 ± 1.06	-3.32 ± 1.08	-3.14 ± 1.02	-3.3 ± 1.04	-3.34 ± 1.07
	AC	-3.02 ± 0.97	-3.0 ± 0.96	-2.82 ± 0.86	-3.0 ± 0.93	-3.03 ± 0.95
	AG	-2.39 ± 0.76	-2.39 ± 0.76	-2.38 ± 0.74	-2.39 ± 0.79	-2.4 ± 0.76
	AT	-4.72 ± 1.21	-4.7 ± 1.17	-4.56 ± 1.02	-4.71 ± 1.18	-4.71 ± 1.21
	CA	4.07 ± 1.22	4.0 ± 1.2	4.13 ± 1.16	4.06 ± 1.19	4.08 ± 1.17
	CC	-1.63 ± 0.68	-1.61 ± 0.68	-1.58 ± 0.7	-1.6 ± 0.7	-1.62 ± 0.68
	CG	3.38 ± 1.26	3.45 ± 1.28	3.2 ± 1.22	3.31 ± 1.28	3.34 ± 1.27
	CT	-2.4 ± 0.78	-2.4 ± 0.76	-2.37 ± 0.72	-2.39 ± 0.77	-2.36 ± 0.75
	GA	-1.12 ± 0.78	-1.1 ± 0.79	-1.11 ± 0.74	-1.1 ± 0.77	-1.11 ± 0.75
	GC	-2.19 ± 0.89	-2.16 ± 0.91	-2.01 ± 0.84	-2.17 ± 0.91	-2.16 ± 0.86
	GG	-1.6 ± 0.68	-1.64 ± 0.7	-1.56 ± 0.7	-1.62 ± 0.7	-1.61 ± 0.69
	GT	-3.05 ± 0.98	-3.03 ± 0.97	-2.78 ± 0.84	-2.99 ± 0.97	-3.01 ± 0.95
	TA	5.44 ± 1.27	5.33 ± 1.29	5.35 ± 1.17	5.36 ± 1.26	5.42 ± 1.28
	TC	-1.11 ± 0.81	-1.07 ± 0.8	-1.14 ± 0.73	-1.11 ± 0.8	-1.13 ± 0.78
	TG	4.03 ± 1.18	4.02 ± 1.2	4.11 ± 1.15	4.05 ± 1.2	4.04 ± 1.22
	TT	-3.32 ± 1.07	-3.35 ± 1.08	-3.17 ± 1.04	-3.32 ± 1.05	-3.31 ± 1.08
MEF2A	AA	-3.34 ± 1.05	-3.31 ± 1.05	-3.3 ± 1.05	-3.31 ± 1.08	-3.34 ± 1.03
	AC	-3.0 ± 0.98	-2.99 ± 0.94	-3.02 ± 0.96	-3.0 ± 0.97	-3.0 ± 0.96
	AG	-2.4 ± 0.78	-2.38 ± 0.78	-2.4 ± 0.77	-2.37 ± 0.78	-2.39 ± 0.79
	AT	-4.72 ± 1.24	-4.7 ± 1.19	-4.79 ± 1.17	-4.69 ± 1.21	-4.72 ± 1.19
	CA	4.06 ± 1.22	4.06 ± 1.19	4.14 ± 1.15	4.1 ± 1.22	4.08 ± 1.19
	CC	-1.61 ± 0.71	-1.6 ± 0.71	-1.6 ± 0.69	-1.62 ± 0.7	-1.63 ± 0.7
	CG	3.42 ± 1.34	3.34 ± 1.21	3.17 ± 1.22	3.43 ± 1.28	3.42 ± 1.24
	CT	-2.41 ± 0.82	-2.39 ± 0.78	-2.37 ± 0.78	-2.4 ± 0.75	-2.41 ± 0.78
	GA	-1.12 ± 0.81	-1.11 ± 0.8	-1.11 ± 0.76	-1.07 ± 0.79	-1.09 ± 0.8
	GC	-2.16 ± 0.89	-2.09 ± 0.89	-2.08 ± 0.9	-2.1 ± 0.87	-2.15 ± 0.88
	GG	-1.62 ± 0.7	-1.59 ± 0.7	-1.59 ± 0.71	-1.62 ± 0.71	-1.58 ± 0.69
	GT	-3.01 ± 1.01	-3.03 ± 0.98	-3.0 ± 1.0	-3.0 ± 0.95	-3.01 ± 0.97
	TA	5.35 ± 1.27	5.4 ± 1.38	5.26 ± 1.29	5.48 ± 1.26	5.41 ± 1.26
	TC	-1.1 ± 0.81	-1.11 ± 0.79	-1.1 ± 0.79	-1.11 ± 0.81	-1.11 ± 0.8
	TG	4.11 ± 1.2	4.11 ± 1.17	4.08 ± 1.16	4.11 ± 1.16	4.05 ± 1.2
	TT	-3.32 ± 1.06	-3.31 ± 1.07	-3.26 ± 1.04	-3.34 ± 1.07	-3.29 ± 1.05
MYC	AA	-3.33 ± 1.07	-3.33 ± 1.06	-3.25 ± 0.91	-3.37 ± 1.04	-3.33 ± 1.08
	AC	-3.02 ± 0.95	-3.03 ± 0.98	-2.88 ± 0.78	-3.03 ± 0.97	-3.02 ± 0.93
	AG	-2.39 ± 0.75	-2.43 ± 0.75	-2.35 ± 0.69	-2.4 ± 0.78	-2.4 ± 0.77

	AT	-4.71 ± 1.2	-4.72 ± 1.22	-4.64 ± 1.04	-4.76 ± 1.22	-4.77 ± 1.21
	CA	4.08 ± 1.21	4.0 ± 1.22	3.73 ± 1.08	4.0 ± 1.19	4.04 ± 1.22
	CC	-1.63 ± 0.71	-1.63 ± 0.69	-1.58 ± 0.64	-1.62 ± 0.7	-1.64 ± 0.68
	CG	3.3 ± 1.22	3.24 ± 1.19	3.12 ± 1.2	3.32 ± 1.29	3.36 ± 1.18
	CT	-2.4 ± 0.75	-2.39 ± 0.76	-2.36 ± 0.69	-2.38 ± 0.73	-2.41 ± 0.76
	GA	-1.14 ± 0.81	-1.12 ± 0.77	-1.19 ± 0.67	-1.14 ± 0.79	-1.12 ± 0.8
	GC	-2.16 ± 0.91	-2.12 ± 0.86	-1.87 ± 0.75	-2.15 ± 0.89	-2.15 ± 0.85
	GG	-1.61 ± 0.68	-1.63 ± 0.69	-1.58 ± 0.66	-1.61 ± 0.71	-1.6 ± 0.7
	GT	-3.01 ± 0.95	-3.02 ± 0.93	-2.85 ± 0.78	-2.98 ± 0.92	-3.01 ± 0.96
	TA	5.37 ± 1.25	5.36 ± 1.27	5.13 ± 1.16	5.43 ± 1.24	5.36 ± 1.27
	TC	-1.09 ± 0.76	-1.13 ± 0.76	-1.21 ± 0.68	-1.15 ± 0.77	-1.09 ± 0.77
	TG	3.99 ± 1.19	3.98 ± 1.18	3.72 ± 1.09	4.0 ± 1.19	4.04 ± 1.2
	TT	-3.32 ± 1.04	-3.34 ± 1.07	-3.28 ± 0.94	-3.36 ± 1.05	-3.32 ± 1.04
NANOG	AA	-3.31 ± 1.07	-3.29 ± 1.07	-3.13 ± 1.01	-3.27 ± 1.05	-3.24 ± 1.07
	AC	-3.03 ± 0.96	-3.08 ± 1.01	-2.95 ± 0.93	-3.01 ± 0.99	-3.01 ± 1.0
	AG	-2.41 ± 0.78	-2.41 ± 0.82	-2.43 ± 0.76	-2.39 ± 0.76	-2.45 ± 0.77
	AT	-4.63 ± 1.2	-4.66 ± 1.23	-4.75 ± 1.06	-4.68 ± 1.23	-4.69 ± 1.26
	CA	4.18 ± 1.21	4.19 ± 1.19	4.2 ± 1.19	4.12 ± 1.19	4.15 ± 1.17
	CC	-1.63 ± 0.73	-1.63 ± 0.71	-1.69 ± 0.72	-1.59 ± 0.69	-1.57 ± 0.71
	CG	3.5 ± 1.15	3.52 ± 1.25	3.16 ± 1.15	3.4 ± 1.28	3.31 ± 1.26
	CT	-2.38 ± 0.76	-2.41 ± 0.82	-2.39 ± 0.77	-2.4 ± 0.79	-2.4 ± 0.78
	GA	-1.1 ± 0.8	-1.11 ± 0.81	-1.07 ± 0.83	-1.09 ± 0.82	-1.08 ± 0.83
	GC	-2.22 ± 0.97	-2.2 ± 0.91	-2.19 ± 0.97	-2.21 ± 0.94	-2.17 ± 0.93
	GG	-1.64 ± 0.7	-1.63 ± 0.7	-1.66 ± 0.71	-1.63 ± 0.7	-1.64 ± 0.72
	GT	-3.02 ± 0.95	-3.03 ± 1.0	-2.97 ± 0.95	-3.03 ± 1.01	-3.06 ± 1.03
	TA	5.54 ± 1.32	5.41 ± 1.31	5.5 ± 1.25	5.42 ± 1.29	5.44 ± 1.28
	TC	-1.08 ± 0.8	-1.11 ± 0.83	-1.05 ± 0.78	-1.07 ± 0.78	-1.06 ± 0.81
	TG	4.27 ± 1.19	4.17 ± 1.19	4.17 ± 1.25	4.17 ± 1.23	4.14 ± 1.17
	TT	-3.3 ± 1.07	-3.32 ± 1.06	-3.1 ± 0.97	-3.31 ± 1.04	-3.29 ± 1.06
NFRKB	AA	-3.34 ± 1.07	-3.36 ± 1.07	-3.33 ± 1.02	-3.34 ± 1.06	-3.36 ± 1.06
	AC	-3.01 ± 0.95	-2.99 ± 0.99	-2.95 ± 0.92	-3.04 ± 0.97	-3.04 ± 1.0
	AG	-2.41 ± 0.77	-2.41 ± 0.77	-2.38 ± 0.68	-2.38 ± 0.77	-2.4 ± 0.75
	AT	-4.7 ± 1.22	-4.69 ± 1.19	-4.67 ± 1.1	-4.68 ± 1.18	-4.74 ± 1.22
	CA	4.06 ± 1.22	4.13 ± 1.21	4.0 ± 1.18	4.06 ± 1.18	4.08 ± 1.22
	CC	-1.6 ± 0.68	-1.58 ± 0.71	-1.58 ± 0.7	-1.63 ± 0.71	-1.61 ± 0.68
	CG	3.36 ± 1.25	3.44 ± 1.28	3.19 ± 1.17	3.25 ± 1.28	3.36 ± 1.3
	CT	-2.4 ± 0.8	-2.39 ± 0.78	-2.38 ± 0.72	-2.39 ± 0.75	-2.4 ± 0.77
	GA	-1.12 ± 0.77	-1.08 ± 0.77	-1.12 ± 0.74	-1.12 ± 0.8	-1.08 ± 0.78
	GC	-2.2 ± 0.88	-2.13 ± 0.88	-2.04 ± 0.83	-2.16 ± 0.89	-2.18 ± 0.89
	GG	-1.65 ± 0.68	-1.65 ± 0.68	-1.58 ± 0.71	-1.6 ± 0.69	-1.61 ± 0.71
	GT	-3.02 ± 0.98	-3.03 ± 0.98	-2.97 ± 0.95	-3.05 ± 1.03	-3.02 ± 0.98
	TA	5.41 ± 1.24	5.36 ± 1.32	5.25 ± 1.31	5.42 ± 1.22	5.35 ± 1.25
	TC	-1.11 ± 0.74	-1.08 ± 0.82	-1.16 ± 0.76	-1.13 ± 0.78	-1.11 ± 0.82
	TG	4.07 ± 1.2	4.0 ± 1.22	4.0 ± 1.11	4.07 ± 1.18	4.09 ± 1.18
	TT	-3.32 ± 1.07	-3.34 ± 1.04	-3.29 ± 1.03	-3.32 ± 1.06	-3.33 ± 1.08
RUNX1	AA	-3.36 ± 1.11	-3.28 ± 1.02	-3.12 ± 0.92	-3.25 ± 1.01	-3.33 ± 1.11
	AC	-3.01 ± 0.91	-2.88 ± 0.9	-2.9 ± 0.88	-3.01 ± 0.92	-2.94 ± 0.92
	AG	-2.33 ± 0.79	-2.42 ± 0.82	-2.37 ± 0.65	-2.35 ± 0.79	-2.43 ± 0.74
	AT	-4.81 ± 1.17	-4.69 ± 1.14	-4.58 ± 1.03	-4.68 ± 1.19	-4.62 ± 1.18
	CA	3.88 ± 1.2	3.93 ± 1.14	3.88 ± 1.18	3.9 ± 1.2	4.18 ± 1.25
	CC	-1.55 ± 0.68	-1.68 ± 0.69	-1.53 ± 0.68	-1.59 ± 0.72	-1.68 ± 0.69
	CG	3.36 ± 1.22	3.28 ± 1.07	2.91 ± 1.12	3.16 ± 1.29	3.11 ± 1.28
	CT	-2.42 ± 0.8	-2.39 ± 0.84	-2.35 ± 0.71	-2.37 ± 0.7	-2.41 ± 0.74
	GA	-1.17 ± 0.76	-1.13 ± 0.77	-1.17 ± 0.68	-1.12 ± 0.73	-1.06 ± 0.77
	GC	-2.17 ± 0.84	-2.08 ± 0.77	-1.89 ± 0.76	-2.12 ± 0.84	-2.09 ± 0.9
	GG	-1.65 ± 0.72	-1.63 ± 0.73	-1.54 ± 0.7	-1.53 ± 0.69	-1.6 ± 0.68

	GT	-3.03 ± 0.98	-3.05 ± 0.92	-2.96 ± 0.89	-3.0 ± 0.96	-2.96 ± 0.91
	TA	5.32 ± 1.49	5.41 ± 1.22	5.25 ± 1.24	5.38 ± 1.25	5.25 ± 1.32
	TC	-1.09 ± 0.81	-1.12 ± 0.78	-1.1 ± 0.68	-1.05 ± 0.68	-1.1 ± 0.76
	TG	3.97 ± 1.22	4.01 ± 1.1	3.89 ± 1.16	3.95 ± 1.14	4.16 ± 1.2
	TT	-3.35 ± 1.1	-3.37 ± 1.03	-3.16 ± 0.94	-3.32 ± 1.12	-3.28 ± 1.03
SPI1	AA	-3.33 ± 1.07	-3.29 ± 1.06	-3.41 ± 0.95	-3.29 ± 1.04	-3.35 ± 1.06
	AC	-3.06 ± 0.97	-3.02 ± 0.94	-3.14 ± 0.91	-3.0 ± 0.94	-3.03 ± 0.95
	AG	-2.39 ± 0.76	-2.4 ± 0.79	-2.43 ± 0.72	-2.39 ± 0.79	-2.38 ± 0.79
	AT	-4.71 ± 1.19	-4.68 ± 1.18	-4.77 ± 1.08	-4.69 ± 1.21	-4.72 ± 1.22
	CA	4.07 ± 1.2	4.09 ± 1.2	3.91 ± 1.19	4.07 ± 1.17	4.1 ± 1.2
	CC	-1.65 ± 0.71	-1.62 ± 0.7	-1.65 ± 0.7	-1.62 ± 0.69	-1.64 ± 0.72
	CG	3.3 ± 1.25	3.3 ± 1.28	3.09 ± 1.2	3.33 ± 1.28	3.38 ± 1.29
	CT	-2.37 ± 0.76	-2.39 ± 0.76	-2.42 ± 0.71	-2.42 ± 0.77	-2.39 ± 0.75
	GA	-1.09 ± 0.79	-1.11 ± 0.79	-1.06 ± 0.68	-1.15 ± 0.79	-1.12 ± 0.78
	GC	-2.16 ± 0.87	-2.18 ± 0.89	-2.09 ± 0.9	-2.19 ± 0.88	-2.16 ± 0.86
	GG	-1.6 ± 0.71	-1.65 ± 0.69	-1.65 ± 0.71	-1.59 ± 0.69	-1.63 ± 0.7
	GT	-3.02 ± 0.96	-3.02 ± 1.0	-3.12 ± 0.93	-3.0 ± 0.97	-3.03 ± 0.98
	TA	5.4 ± 1.3	5.39 ± 1.34	5.23 ± 1.21	5.44 ± 1.23	5.42 ± 1.27
	TC	-1.12 ± 0.8	-1.1 ± 0.77	-1.11 ± 0.67	-1.09 ± 0.79	-1.11 ± 0.78
	TG	4.08 ± 1.19	4.07 ± 1.19	3.89 ± 1.21	4.05 ± 1.19	4.09 ± 1.2
	TT	-3.33 ± 1.08	-3.32 ± 1.06	-3.38 ± 0.94	-3.31 ± 1.09	-3.29 ± 1.06
TP53	AA	-3.33 ± 1.09	-3.36 ± 1.03	-3.27 ± 0.93	-3.3 ± 1.07	-3.34 ± 1.04
	AC	-2.98 ± 0.93	-3.02 ± 0.99	-2.94 ± 0.81	-2.96 ± 0.91	-3.01 ± 0.96
	AG	-2.4 ± 0.75	-2.4 ± 0.73	-2.38 ± 0.68	-2.42 ± 0.75	-2.4 ± 0.76
	AT	-4.74 ± 1.22	-4.71 ± 1.19	-4.7 ± 1.07	-4.76 ± 1.17	-4.76 ± 1.2
	CA	4.03 ± 1.2	4.03 ± 1.2	3.75 ± 1.11	3.95 ± 1.21	4.02 ± 1.18
	CC	-1.62 ± 0.68	-1.66 ± 0.7	-1.61 ± 0.63	-1.63 ± 0.7	-1.64 ± 0.68
	CG	3.23 ± 1.21	3.28 ± 1.23	2.99 ± 1.16	3.31 ± 1.22	3.2 ± 1.27
	CT	-2.38 ± 0.77	-2.39 ± 0.73	-2.38 ± 0.7	-2.4 ± 0.77	-2.39 ± 0.74
	GA	-1.1 ± 0.75	-1.12 ± 0.76	-1.16 ± 0.71	-1.12 ± 0.78	-1.14 ± 0.8
	GC	-2.16 ± 0.91	-2.13 ± 0.89	-1.92 ± 0.77	-2.17 ± 0.87	-2.14 ± 0.94
	GG	-1.63 ± 0.7	-1.62 ± 0.7	-1.59 ± 0.64	-1.62 ± 0.68	-1.58 ± 0.68
	GT	-2.99 ± 0.92	-3.0 ± 0.93	-2.9 ± 0.81	-3.0 ± 0.94	-3.01 ± 0.98
	TA	5.4 ± 1.29	5.29 ± 1.25	5.14 ± 1.22	5.37 ± 1.23	5.38 ± 1.3
	TC	-1.15 ± 0.78	-1.14 ± 0.75	-1.18 ± 0.7	-1.15 ± 0.77	-1.15 ± 0.77
	TG	4.01 ± 1.18	4.03 ± 1.19	3.74 ± 1.1	4.01 ± 1.2	4.04 ± 1.17
	TT	-3.33 ± 1.02	-3.38 ± 1.06	-3.28 ± 0.93	-3.32 ± 1.08	-3.38 ± 1.07

Table S12. Average helical twist values from selected positions relative to the binding site (prolonged peak center). The shape feature values were acquired using deepDNAshape from 50 bp windows.

		-3000 bp	-1500 bp	at binding site	1500 bp	3000 bp
CTCF	AA	35.85 ± 0.9	35.84 ± 0.9	35.57 ± 0.85	35.85 ± 0.89	35.86 ± 0.91
	AC	34.51 ± 0.77	34.53 ± 0.78	34.32 ± 0.64	34.52 ± 0.79	34.52 ± 0.78
	AG	31.84 ± 0.66	31.85 ± 0.66	31.63 ± 0.51	31.84 ± 0.66	31.84 ± 0.65
	AT	32.67 ± 0.8	32.68 ± 0.82	32.43 ± 0.71	32.68 ± 0.81	32.69 ± 0.82
	CA	34.62 ± 0.57	34.62 ± 0.56	34.44 ± 0.52	34.62 ± 0.57	34.62 ± 0.56
	CC	33.9 ± 0.59	33.88 ± 0.58	33.81 ± 0.53	33.9 ± 0.58	33.91 ± 0.6
	CG	32.99 ± 0.6	32.96 ± 0.6	32.74 ± 0.55	32.94 ± 0.58	32.96 ± 0.6
	CT	31.83 ± 0.65	31.83 ± 0.65	31.64 ± 0.52	31.84 ± 0.64	31.84 ± 0.64
	GA	35.78 ± 0.68	35.78 ± 0.66	35.73 ± 0.58	35.77 ± 0.67	35.78 ± 0.67
	GC	36.67 ± 0.62	36.69 ± 0.61	36.57 ± 0.53	36.67 ± 0.62	36.68 ± 0.62
	GG	33.91 ± 0.59	33.89 ± 0.58	33.81 ± 0.52	33.89 ± 0.58	33.89 ± 0.58
	GT	34.52 ± 0.79	34.52 ± 0.79	34.33 ± 0.66	34.53 ± 0.79	34.52 ± 0.78
	TA	34.66 ± 0.46	34.65 ± 0.46	34.52 ± 0.4	34.63 ± 0.45	34.64 ± 0.45
	TC	35.79 ± 0.69	35.77 ± 0.67	35.71 ± 0.59	35.76 ± 0.67	35.78 ± 0.67
	TG	34.61 ± 0.56	34.61 ± 0.55	34.43 ± 0.51	34.62 ± 0.56	34.62 ± 0.56
	TT	35.86 ± 0.9	35.85 ± 0.89	35.58 ± 0.86	35.86 ± 0.9	35.86 ± 0.9
FOXK2	AA	35.91 ± 0.89	35.88 ± 0.92	35.71 ± 0.83	35.87 ± 0.91	35.87 ± 0.9
	AC	34.53 ± 0.8	34.53 ± 0.79	34.42 ± 0.71	34.51 ± 0.76	34.53 ± 0.78
	AG	31.83 ± 0.65	31.84 ± 0.65	31.81 ± 0.61	31.84 ± 0.65	31.83 ± 0.66
	AT	32.69 ± 0.81	32.68 ± 0.81	32.49 ± 0.69	32.68 ± 0.81	32.7 ± 0.82
	CA	34.6 ± 0.56	34.62 ± 0.56	34.67 ± 0.57	34.62 ± 0.56	34.61 ± 0.55
	CC	33.89 ± 0.58	33.88 ± 0.57	33.84 ± 0.53	33.88 ± 0.57	33.91 ± 0.59
	CG	33.0 ± 0.6	32.97 ± 0.59	32.91 ± 0.57	32.92 ± 0.58	32.97 ± 0.59
	CT	31.83 ± 0.65	31.84 ± 0.64	31.8 ± 0.6	31.84 ± 0.65	31.84 ± 0.65
	GA	35.76 ± 0.68	35.78 ± 0.68	35.74 ± 0.62	35.78 ± 0.67	35.77 ± 0.67
	GC	36.68 ± 0.62	36.67 ± 0.63	36.56 ± 0.62	36.67 ± 0.62	36.67 ± 0.61
	GG	33.9 ± 0.58	33.89 ± 0.57	33.84 ± 0.54	33.9 ± 0.58	33.91 ± 0.59
	GT	34.53 ± 0.78	34.53 ± 0.8	34.4 ± 0.69	34.51 ± 0.77	34.52 ± 0.78
	TA	34.62 ± 0.45	34.65 ± 0.46	34.58 ± 0.42	34.65 ± 0.46	34.64 ± 0.45
	TC	35.79 ± 0.69	35.74 ± 0.67	35.75 ± 0.62	35.78 ± 0.69	35.79 ± 0.68
	TG	34.61 ± 0.55	34.61 ± 0.57	34.67 ± 0.56	34.6 ± 0.55	34.6 ± 0.55
	TT	35.87 ± 0.91	35.89 ± 0.9	35.71 ± 0.83	35.89 ± 0.92	35.87 ± 0.91
MEF2A	AA	35.85 ± 0.9	35.92 ± 0.89	35.84 ± 0.85	35.89 ± 0.91	35.89 ± 0.9
	AC	34.5 ± 0.78	34.5 ± 0.78	34.55 ± 0.8	34.54 ± 0.81	34.54 ± 0.8
	AG	31.87 ± 0.65	31.85 ± 0.64	31.85 ± 0.65	31.86 ± 0.65	31.88 ± 0.67
	AT	32.71 ± 0.83	32.69 ± 0.82	32.71 ± 0.83	32.68 ± 0.84	32.7 ± 0.82
	CA	34.64 ± 0.57	34.61 ± 0.56	34.65 ± 0.56	34.62 ± 0.57	34.63 ± 0.55
	CC	33.92 ± 0.6	33.9 ± 0.56	33.88 ± 0.59	33.89 ± 0.59	33.91 ± 0.58
	CG	32.98 ± 0.61	32.92 ± 0.55	32.87 ± 0.56	32.99 ± 0.63	33.01 ± 0.6
	CT	31.87 ± 0.67	31.85 ± 0.65	31.84 ± 0.64	31.89 ± 0.66	31.86 ± 0.67
	GA	35.82 ± 0.69	35.8 ± 0.69	35.74 ± 0.67	35.77 ± 0.69	35.8 ± 0.7
	GC	36.69 ± 0.62	36.63 ± 0.63	36.62 ± 0.65	36.63 ± 0.62	36.67 ± 0.62
	GG	33.91 ± 0.61	33.9 ± 0.6	33.91 ± 0.59	33.91 ± 0.6	33.91 ± 0.6
	GT	34.54 ± 0.83	34.55 ± 0.78	34.56 ± 0.83	34.56 ± 0.79	34.54 ± 0.8
	TA	34.65 ± 0.45	34.64 ± 0.47	34.67 ± 0.48	34.62 ± 0.46	34.65 ± 0.46
	TC	35.78 ± 0.68	35.79 ± 0.68	35.77 ± 0.67	35.78 ± 0.7	35.79 ± 0.7
	TG	34.64 ± 0.56	34.62 ± 0.56	34.62 ± 0.53	34.63 ± 0.56	34.62 ± 0.56
	TT	35.89 ± 0.9	35.88 ± 0.92	35.82 ± 0.87	35.88 ± 0.9	35.87 ± 0.88
MYC	AA	35.87 ± 0.93	35.85 ± 0.9	35.54 ± 0.88	35.85 ± 0.91	35.86 ± 0.91
	AC	34.52 ± 0.79	34.51 ± 0.79	34.32 ± 0.67	34.54 ± 0.79	34.52 ± 0.77
	AG	31.83 ± 0.64	31.82 ± 0.64	31.69 ± 0.55	31.85 ± 0.66	31.83 ± 0.65

	AT	32.71 ± 0.83	32.69 ± 0.84	32.47 ± 0.73	32.72 ± 0.84	32.7 ± 0.82
	CA	34.61 ± 0.55	34.6 ± 0.55	34.48 ± 0.51	34.59 ± 0.54	34.6 ± 0.56
	CC	33.87 ± 0.58	33.89 ± 0.59	33.75 ± 0.47	33.86 ± 0.55	33.87 ± 0.57
	CG	32.95 ± 0.58	32.89 ± 0.55	32.86 ± 0.57	32.94 ± 0.61	32.97 ± 0.59
	CT	31.81 ± 0.63	31.82 ± 0.63	31.63 ± 0.51	31.82 ± 0.64	31.82 ± 0.66
	GA	35.74 ± 0.68	35.76 ± 0.67	35.66 ± 0.58	35.76 ± 0.67	35.76 ± 0.69
	GC	36.68 ± 0.62	36.64 ± 0.6	36.48 ± 0.58	36.66 ± 0.62	36.67 ± 0.6
	GG	33.9 ± 0.58	33.87 ± 0.57	33.76 ± 0.48	33.85 ± 0.56	33.89 ± 0.59
	GT	34.49 ± 0.78	34.52 ± 0.77	34.3 ± 0.67	34.51 ± 0.75	34.52 ± 0.79
	TA	34.63 ± 0.45	34.62 ± 0.44	34.54 ± 0.4	34.62 ± 0.44	34.64 ± 0.45
	TC	35.76 ± 0.68	35.77 ± 0.67	35.68 ± 0.58	35.76 ± 0.66	35.74 ± 0.66
	TG	34.57 ± 0.55	34.58 ± 0.56	34.48 ± 0.51	34.59 ± 0.55	34.58 ± 0.55
	TT	35.83 ± 0.89	35.85 ± 0.92	35.57 ± 0.87	35.89 ± 0.94	35.85 ± 0.91
NANOG	AA	35.91 ± 0.91	35.89 ± 0.87	35.71 ± 0.76	35.86 ± 0.86	35.87 ± 0.85
	AC	34.56 ± 0.76	34.61 ± 0.83	34.47 ± 0.72	34.55 ± 0.81	34.55 ± 0.78
	AG	31.89 ± 0.67	31.89 ± 0.67	31.87 ± 0.67	31.86 ± 0.64	31.9 ± 0.65
	AT	32.69 ± 0.82	32.69 ± 0.8	32.64 ± 0.74	32.7 ± 0.82	32.72 ± 0.87
	CA	34.66 ± 0.56	34.68 ± 0.58	34.67 ± 0.55	34.66 ± 0.56	34.66 ± 0.56
	CC	33.92 ± 0.6	33.9 ± 0.62	33.86 ± 0.57	33.94 ± 0.6	33.91 ± 0.6
	CG	33.02 ± 0.61	33.06 ± 0.6	32.84 ± 0.48	33.02 ± 0.64	32.96 ± 0.58
	CT	31.88 ± 0.68	31.88 ± 0.69	31.88 ± 0.67	31.87 ± 0.65	31.92 ± 0.69
	GA	35.79 ± 0.7	35.84 ± 0.69	35.82 ± 0.67	35.79 ± 0.69	35.77 ± 0.72
	GC	36.69 ± 0.65	36.67 ± 0.64	36.64 ± 0.66	36.71 ± 0.64	36.65 ± 0.63
	GG	33.9 ± 0.59	33.93 ± 0.62	33.87 ± 0.6	33.96 ± 0.59	33.93 ± 0.63
	GT	34.57 ± 0.8	34.57 ± 0.77	34.5 ± 0.78	34.56 ± 0.81	34.58 ± 0.82
	TA	34.65 ± 0.45	34.65 ± 0.47	34.58 ± 0.45	34.66 ± 0.46	34.67 ± 0.47
	TC	35.78 ± 0.71	35.81 ± 0.69	35.78 ± 0.67	35.79 ± 0.69	35.79 ± 0.72
	TG	34.7 ± 0.57	34.64 ± 0.55	34.66 ± 0.56	34.69 ± 0.6	34.66 ± 0.57
	TT	35.89 ± 0.91	35.89 ± 0.89	35.73 ± 0.78	35.89 ± 0.87	35.9 ± 0.88
NFRKB	AA	35.89 ± 0.91	35.9 ± 0.92	35.77 ± 0.9	35.87 ± 0.88	35.9 ± 0.88
	AC	34.55 ± 0.79	34.52 ± 0.78	34.46 ± 0.72	34.53 ± 0.8	34.51 ± 0.79
	AG	31.86 ± 0.67	31.86 ± 0.66	31.79 ± 0.59	31.84 ± 0.67	31.86 ± 0.66
	AT	32.68 ± 0.81	32.69 ± 0.81	32.61 ± 0.78	32.69 ± 0.78	32.71 ± 0.81
	CA	34.62 ± 0.57	34.64 ± 0.58	34.58 ± 0.55	34.61 ± 0.55	34.6 ± 0.55
	CC	33.93 ± 0.6	33.89 ± 0.6	33.82 ± 0.52	33.93 ± 0.58	33.91 ± 0.6
	CG	32.95 ± 0.61	32.95 ± 0.58	32.86 ± 0.53	32.92 ± 0.6	32.95 ± 0.58
	CT	31.86 ± 0.66	31.85 ± 0.64	31.79 ± 0.62	31.85 ± 0.63	31.84 ± 0.67
	GA	35.81 ± 0.68	35.83 ± 0.67	35.75 ± 0.64	35.76 ± 0.67	35.76 ± 0.69
	GC	36.71 ± 0.61	36.64 ± 0.63	36.58 ± 0.6	36.67 ± 0.62	36.7 ± 0.61
	GG	33.91 ± 0.6	33.86 ± 0.59	33.84 ± 0.54	33.88 ± 0.58	33.9 ± 0.6
	GT	34.53 ± 0.78	34.51 ± 0.79	34.48 ± 0.79	34.54 ± 0.81	34.55 ± 0.81
	TA	34.65 ± 0.48	34.64 ± 0.45	34.6 ± 0.43	34.63 ± 0.45	34.65 ± 0.45
	TC	35.79 ± 0.69	35.75 ± 0.69	35.79 ± 0.66	35.79 ± 0.68	35.79 ± 0.7
	TG	34.6 ± 0.56	34.58 ± 0.56	34.6 ± 0.55	34.62 ± 0.56	34.66 ± 0.56
	TT	35.87 ± 0.92	35.87 ± 0.89	35.73 ± 0.9	35.88 ± 0.9	35.92 ± 0.91
RUNX1	AA	35.92 ± 0.95	35.85 ± 0.92	35.63 ± 0.91	35.85 ± 0.85	35.9 ± 0.92
	AC	34.47 ± 0.74	34.43 ± 0.74	34.4 ± 0.84	34.46 ± 0.77	34.49 ± 0.75
	AG	31.8 ± 0.65	31.87 ± 0.72	31.61 ± 0.52	31.81 ± 0.63	31.79 ± 0.62
	AT	32.69 ± 0.76	32.65 ± 0.8	32.52 ± 0.66	32.64 ± 0.79	32.65 ± 0.78
	CA	34.54 ± 0.55	34.61 ± 0.55	34.55 ± 0.57	34.56 ± 0.54	34.61 ± 0.57
	CC	33.9 ± 0.58	33.88 ± 0.59	33.85 ± 0.54	33.94 ± 0.58	33.88 ± 0.57
	CG	32.93 ± 0.64	32.85 ± 0.52	32.78 ± 0.44	32.9 ± 0.56	32.8 ± 0.6
	CT	31.86 ± 0.69	31.87 ± 0.67	31.74 ± 0.64	31.77 ± 0.64	31.82 ± 0.66
	GA	35.74 ± 0.62	35.8 ± 0.6	35.67 ± 0.64	35.81 ± 0.73	35.71 ± 0.67
	GC	36.69 ± 0.57	36.64 ± 0.58	36.44 ± 0.58	36.69 ± 0.6	36.6 ± 0.61
	GG	33.88 ± 0.57	33.87 ± 0.57	33.82 ± 0.55	33.83 ± 0.5	33.91 ± 0.58

	GT	34.52 ± 0.81	34.52 ± 0.79	34.46 ± 0.87	34.5 ± 0.76	34.48 ± 0.77
	TA	34.64 ± 0.5	34.58 ± 0.42	34.58 ± 0.36	34.61 ± 0.44	34.64 ± 0.48
	TC	35.73 ± 0.7	35.71 ± 0.65	35.72 ± 0.59	35.75 ± 0.66	35.78 ± 0.64
	TG	34.58 ± 0.53	34.57 ± 0.54	34.59 ± 0.47	34.56 ± 0.51	34.62 ± 0.57
	TT	35.89 ± 0.96	35.83 ± 0.92	35.55 ± 0.71	35.86 ± 0.92	35.81 ± 0.89
SPI1	AA	35.88 ± 0.92	35.86 ± 0.89	35.68 ± 0.83	35.86 ± 0.87	35.88 ± 0.89
	AC	34.56 ± 0.79	34.55 ± 0.79	34.58 ± 0.78	34.51 ± 0.76	34.54 ± 0.77
	AG	31.85 ± 0.63	31.85 ± 0.67	31.89 ± 0.63	31.84 ± 0.65	31.86 ± 0.66
	AT	32.69 ± 0.82	32.68 ± 0.81	32.63 ± 0.77	32.69 ± 0.83	32.72 ± 0.83
	CA	34.6 ± 0.56	34.63 ± 0.56	34.51 ± 0.52	34.61 ± 0.55	34.64 ± 0.57
	CC	33.89 ± 0.6	33.9 ± 0.59	33.9 ± 0.55	33.92 ± 0.59	33.92 ± 0.58
	CG	32.95 ± 0.59	32.89 ± 0.56	32.85 ± 0.56	32.97 ± 0.59	32.98 ± 0.6
	CT	31.84 ± 0.64	31.84 ± 0.63	31.89 ± 0.63	31.86 ± 0.67	31.86 ± 0.65
	GA	35.76 ± 0.68	35.77 ± 0.67	35.72 ± 0.61	35.79 ± 0.69	35.78 ± 0.69
	GC	36.67 ± 0.61	36.68 ± 0.62	36.62 ± 0.63	36.69 ± 0.62	36.69 ± 0.61
	GG	33.91 ± 0.6	33.91 ± 0.6	33.89 ± 0.55	33.9 ± 0.61	33.92 ± 0.6
	GT	34.57 ± 0.81	34.53 ± 0.78	34.56 ± 0.76	34.54 ± 0.79	34.55 ± 0.79
	TA	34.66 ± 0.46	34.64 ± 0.45	34.57 ± 0.42	34.65 ± 0.46	34.66 ± 0.46
	TC	35.82 ± 0.68	35.79 ± 0.67	35.76 ± 0.61	35.75 ± 0.68	35.79 ± 0.69
	TG	34.62 ± 0.57	34.61 ± 0.56	34.51 ± 0.54	34.62 ± 0.56	34.64 ± 0.55
	TT	35.87 ± 0.9	35.87 ± 0.9	35.66 ± 0.82	35.88 ± 0.9	35.86 ± 0.89
TP53	AA	35.87 ± 0.92	35.84 ± 0.91	35.54 ± 0.85	35.85 ± 0.87	35.86 ± 0.91
	AC	34.5 ± 0.75	34.51 ± 0.81	34.4 ± 0.69	34.51 ± 0.79	34.51 ± 0.79
	AG	31.85 ± 0.67	31.8 ± 0.64	31.66 ± 0.57	31.83 ± 0.65	31.81 ± 0.64
	AT	32.72 ± 0.83	32.68 ± 0.8	32.53 ± 0.79	32.7 ± 0.81	32.73 ± 0.81
	CA	34.58 ± 0.54	34.59 ± 0.54	34.46 ± 0.51	34.56 ± 0.54	34.59 ± 0.56
	CC	33.89 ± 0.6	33.88 ± 0.59	33.75 ± 0.49	33.88 ± 0.59	33.87 ± 0.59
	CG	32.88 ± 0.57	32.89 ± 0.55	32.78 ± 0.5	32.9 ± 0.54	32.92 ± 0.59
	CT	31.81 ± 0.63	31.79 ± 0.63	31.68 ± 0.58	31.84 ± 0.64	31.81 ± 0.63
	GA	35.76 ± 0.64	35.74 ± 0.66	35.68 ± 0.59	35.77 ± 0.66	35.78 ± 0.69
	GC	36.65 ± 0.63	36.65 ± 0.62	36.51 ± 0.58	36.69 ± 0.63	36.64 ± 0.63
	GG	33.88 ± 0.61	33.89 ± 0.58	33.74 ± 0.47	33.89 ± 0.59	33.85 ± 0.57
	GT	34.51 ± 0.78	34.5 ± 0.78	34.37 ± 0.7	34.51 ± 0.81	34.5 ± 0.81
	TA	34.63 ± 0.45	34.62 ± 0.46	34.57 ± 0.41	34.59 ± 0.44	34.64 ± 0.44
	TC	35.76 ± 0.66	35.74 ± 0.66	35.7 ± 0.59	35.78 ± 0.66	35.82 ± 0.68
	TG	34.59 ± 0.56	34.6 ± 0.56	34.46 ± 0.51	34.6 ± 0.55	34.61 ± 0.55
	TT	35.84 ± 0.92	35.85 ± 0.95	35.59 ± 0.9	35.85 ± 0.93	35.88 ± 0.91

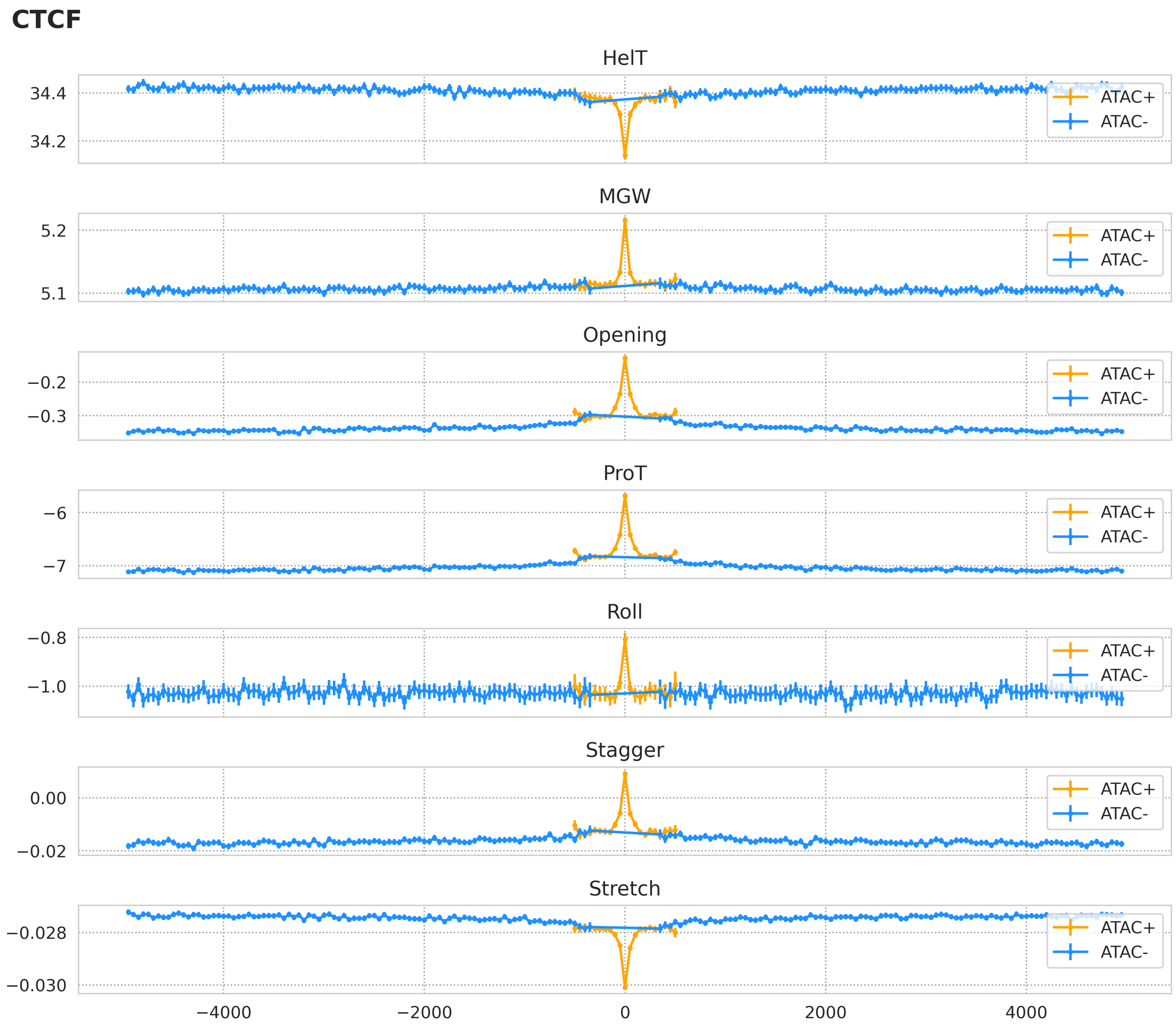

Fig. S24. Average DNA shape values (from top to bottom: helical twist, minor groove width, opening, propeller twist, roll, stagger, stretch) per every 25th offset for prolonged peaks in experiments targeting CTCF. We distinguish ATAC+ (orange, k-mers overlapping with an ATAC-seq peak in the same cell line) and ATAC- (blue, k-mers overlapping with **no** ATAC-seq peak in the same cell line) k-mers. Value is shown if at least 25% peaks at position are seen open/closed.

FOXK2

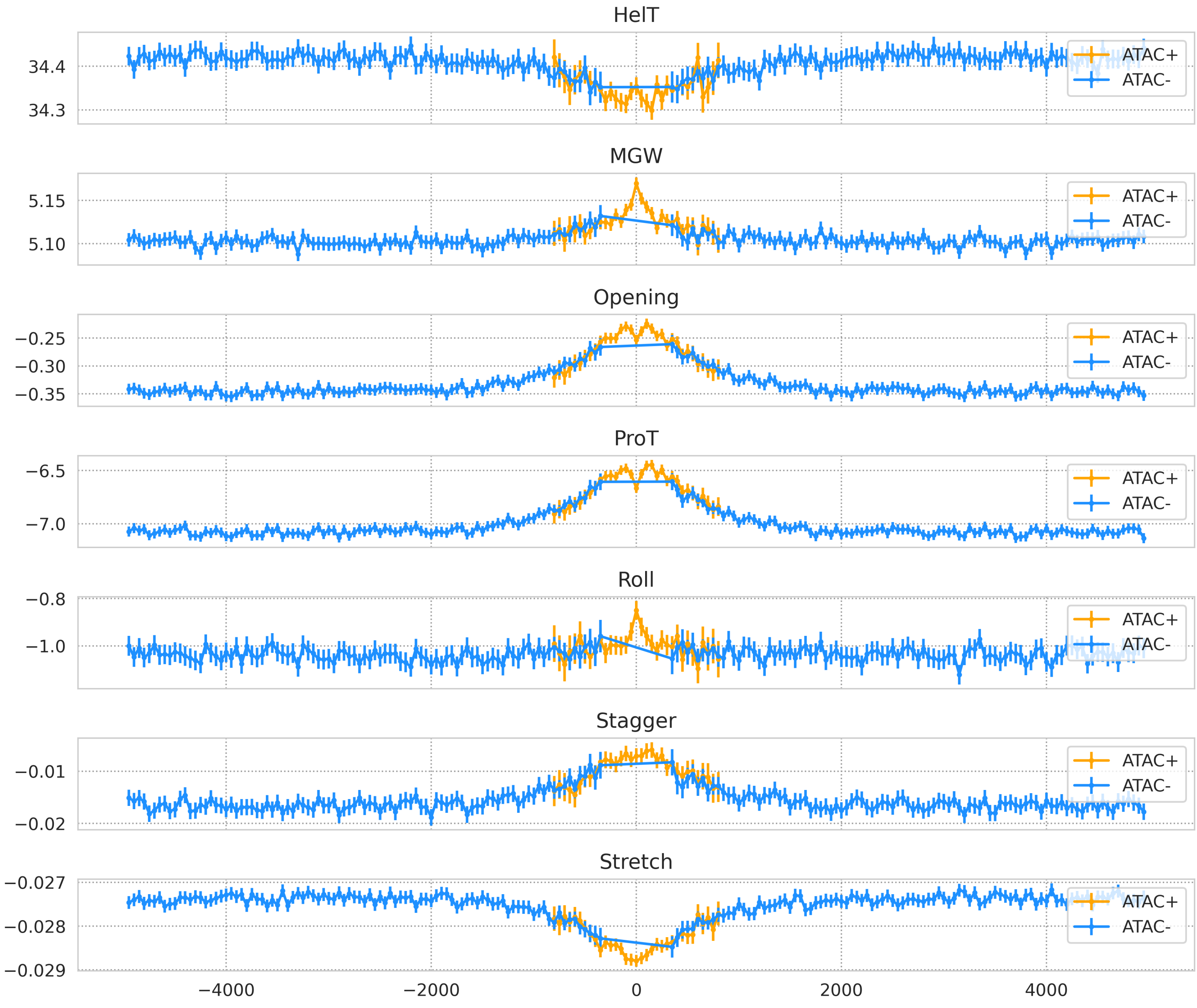

Fig. S25. Average DNA shape values (from top to bottom: helical twist, minor groove width, opening, propeller twist, roll, stagger, stretch) per every 25th offset for prolonged peaks in experiments targeting FOXK2. We distinguish ATAC+ (orange, k-mers overlapping with an ATAC-seq peak in the same cell line) and ATAC- (blue, k-mers overlapping with **no** ATAC-seq peak in the same cell line) k-mers. Value is shown if at least 25% peaks at position are seen open/closed.

IRF1

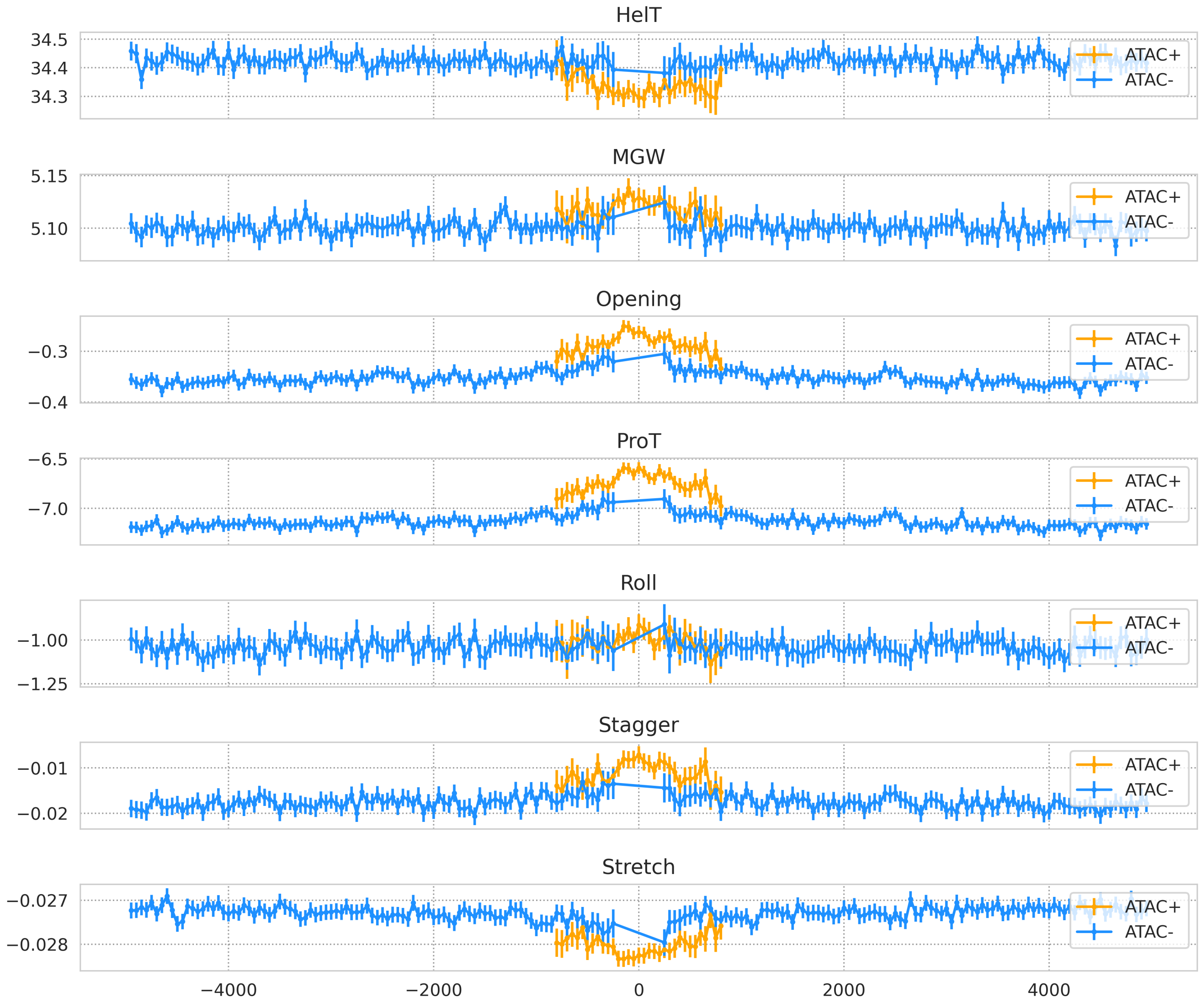

Fig. S26. Average DNA shape values (from top to bottom: helical twist, minor groove width, opening, propeller twist, roll, stagger, stretch) per every 25th offset for prolonged peaks in experiments targeting IRF1. We distinguish ATAC+ (orange, k-mers overlapping with an ATAC-seq peak in the same cell line) and ATAC- (blue, k-mers overlapping with **no** ATAC-seq peak in the same cell line) k-mers. Value is shown if at least 25% peaks at position are seen open/closed.

MEF2A

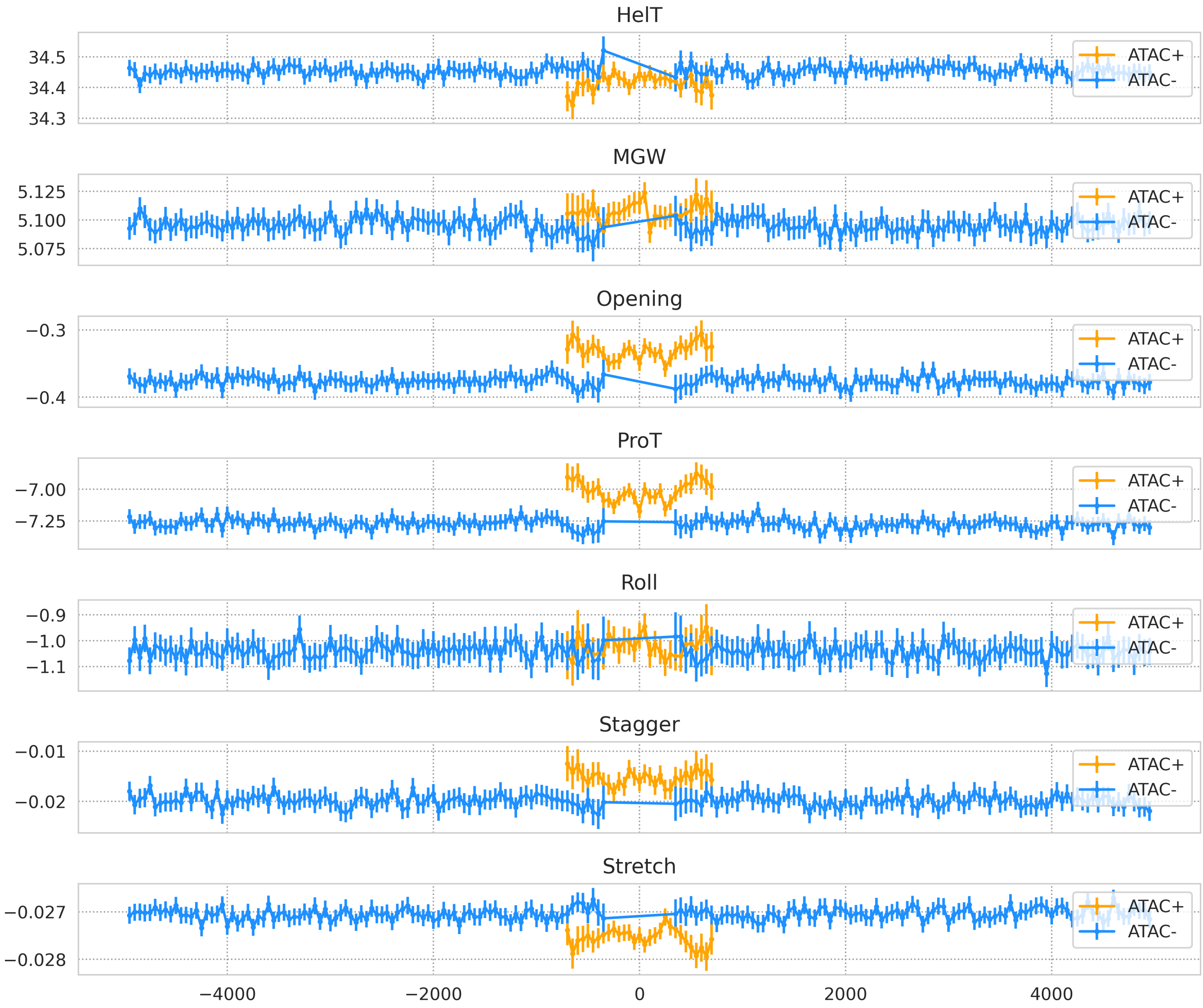

Fig. S27. Average DNA shape values (from top to bottom: helical twist, minor groove width, opening, propeller twist, roll, stagger, stretch) per every 25th offset for prolonged peaks in experiments targeting MEF2A. We distinguish ATAC+ (orange, k-mers overlapping with an ATAC-seq peak in the same cell line) and ATAC- (blue, k-mers overlapping with **no** ATAC-seq peak in the same cell line) k-mers. Value is shown if at least 25% peaks at position are seen open/closed.

MYC

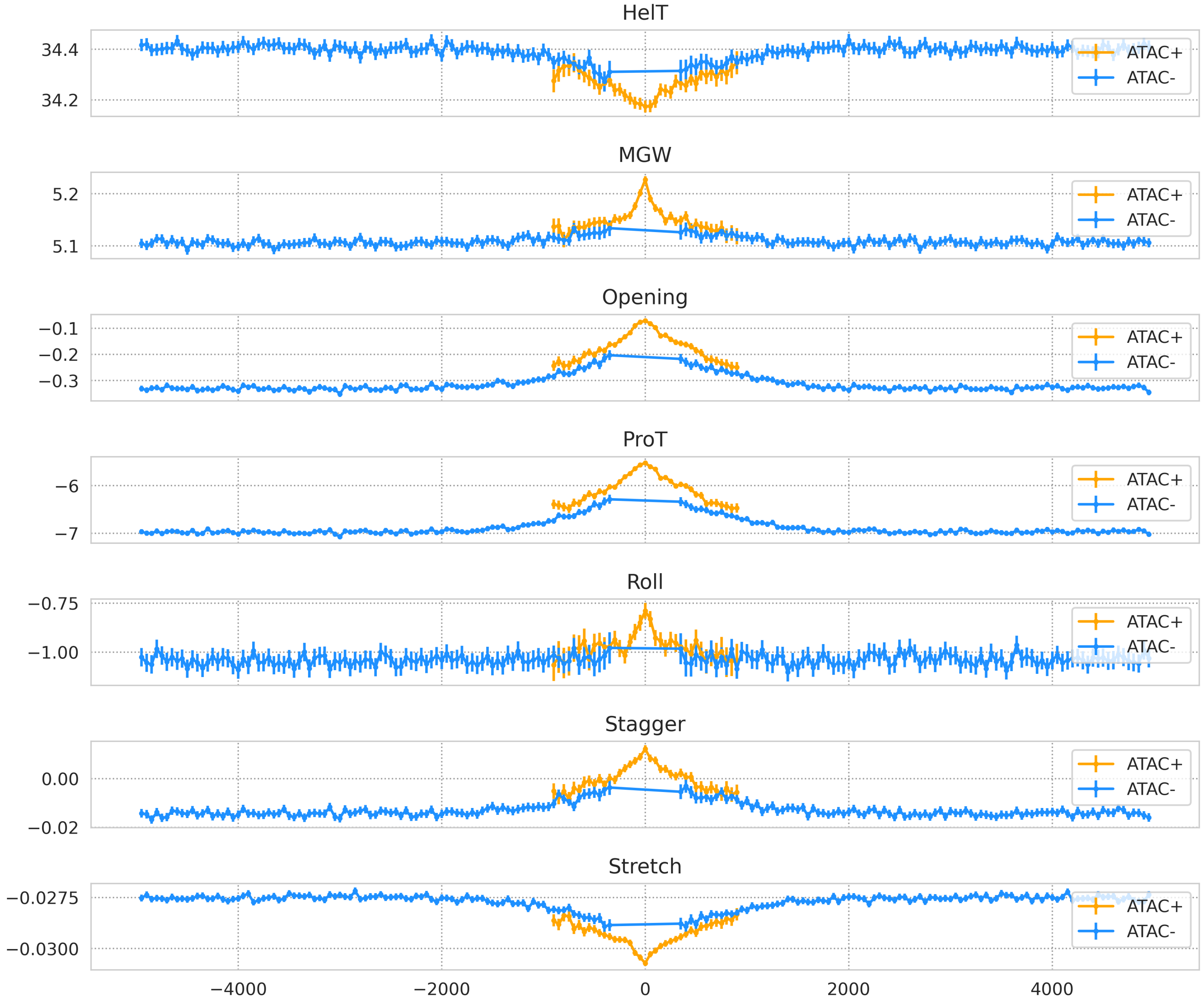

Fig. S28. Average DNA shape values (from top to bottom: helical twist, minor groove width, opening, propeller twist, roll, stagger, stretch) per every 25th offset for prolonged peaks in experiments targeting MYC. We distinguish ATAC+ (orange, k-mers overlapping with an ATAC-seq peak in the same cell line) and ATAC- (blue, k-mers overlapping with **no** ATAC-seq peak in the same cell line) k-mers. Value is shown if at least 25% peaks at position are seen open/closed.

NANOG

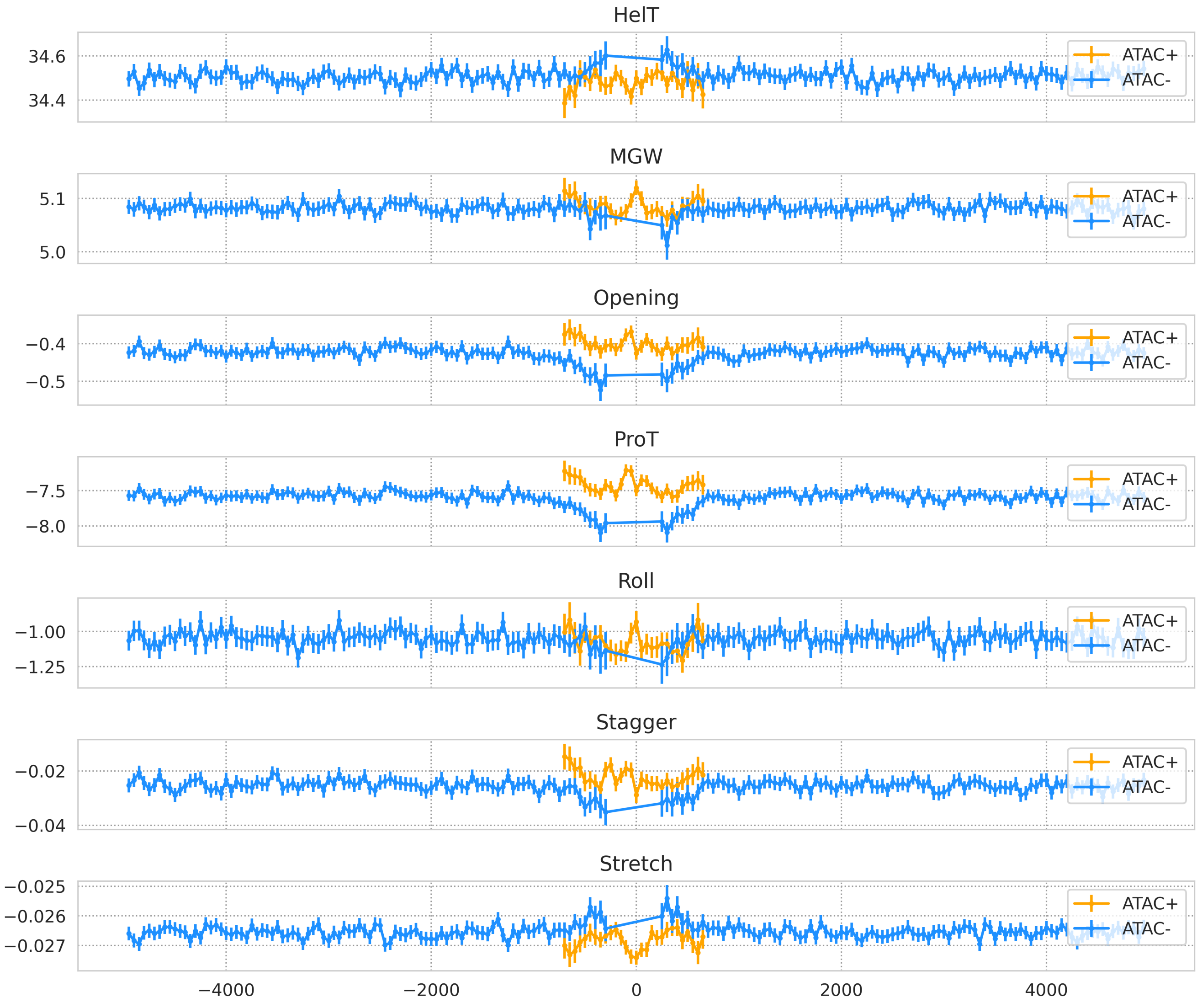

Fig. S29. Average DNA shape values (from top to bottom: helical twist, minor groove width, opening, propeller twist, roll, stagger, stretch) per every 25th offset for prolonged peaks in experiments targeting NANOG. We distinguish ATAC+ (orange, k-mers overlapping with an ATAC-seq peak in the same cell line) and ATAC- (blue, k-mers overlapping with **no** ATAC-seq peak in the same cell line) k-mers. Value is shown if at least 25% peaks at position are seen open/closed.

NFRKB

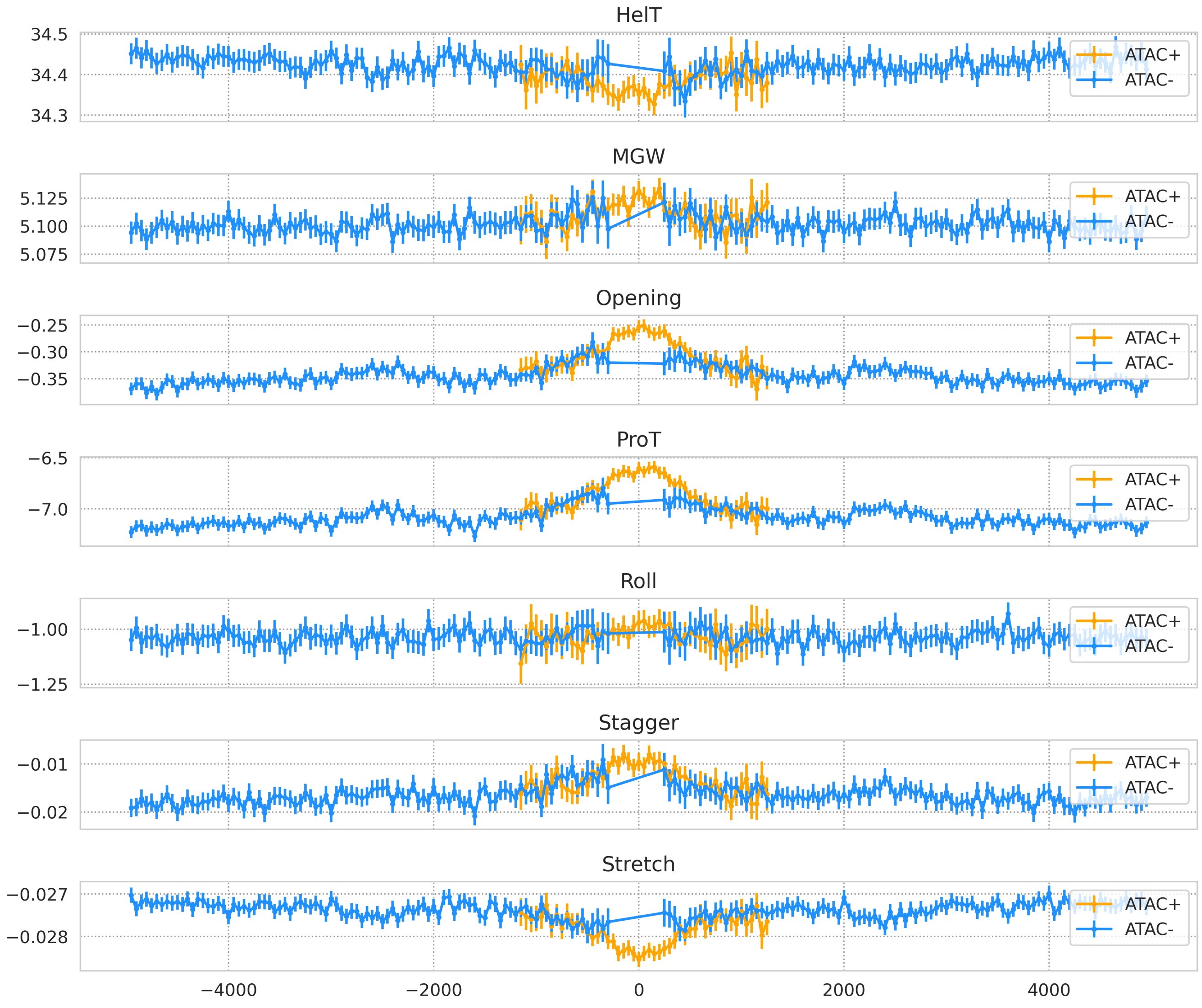

Fig. S30. Average DNA shape values (from top to bottom: helical twist, minor groove width, opening, propeller twist, roll, stagger, stretch) per every 25th offset for prolonged peaks in experiments targeting NFRKB. We distinguish ATAC+ (orange, k-mers overlapping with an ATAC-seq peak in the same cell line) and ATAC- (blue, k-mers overlapping with **no** ATAC-seq peak in the same cell line) k-mers. Value is shown if at least 25% peaks at position are seen open/closed.

RUNX1

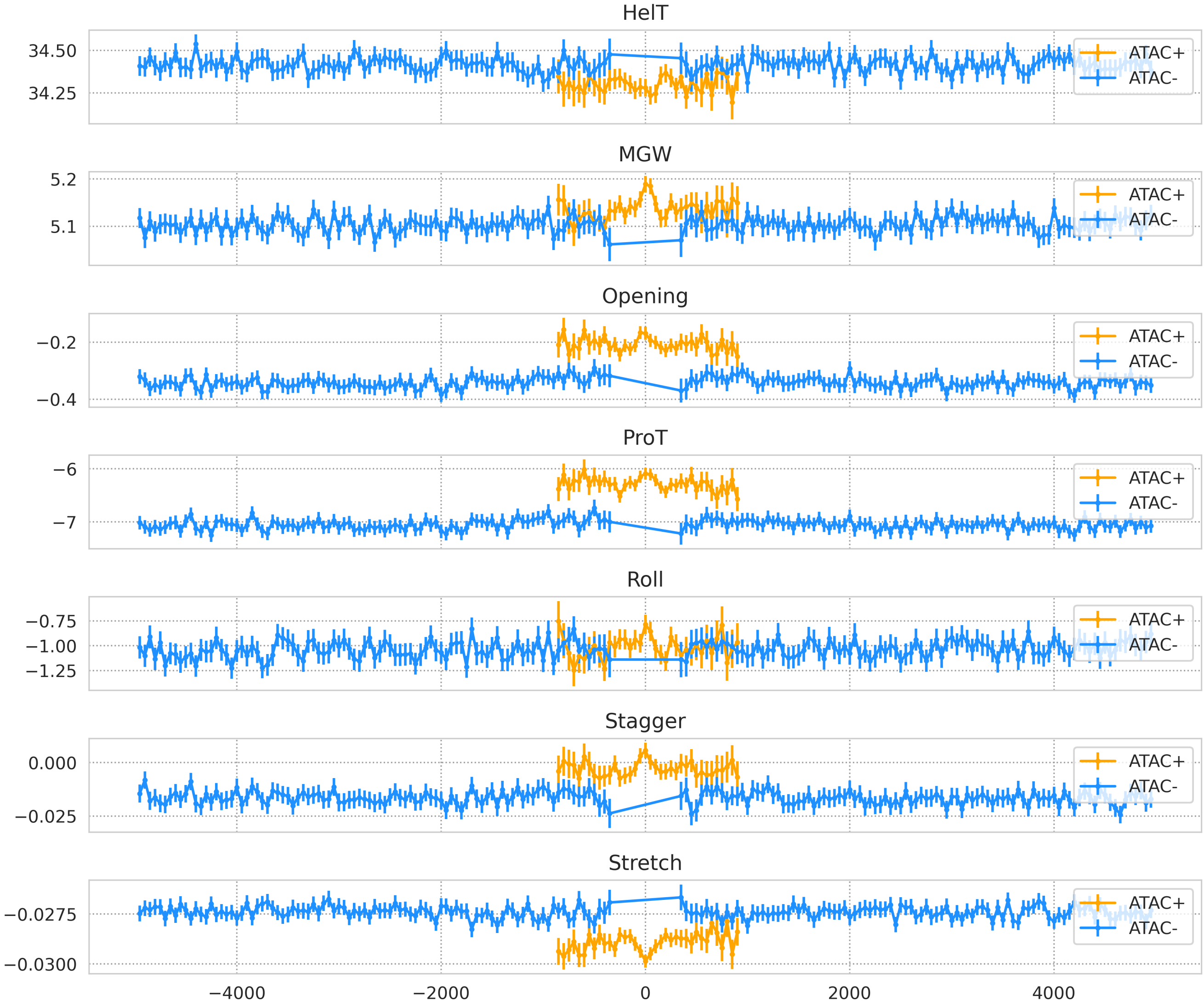

Fig. S31. Average DNA shape values (from top to bottom: helical twist, minor groove width, opening, propeller twist, roll, stagger, stretch) per every 25th offset for prolonged peaks in experiments targeting RUNX1. We distinguish ATAC+ (orange, k-mers overlapping with an ATAC-seq peak in the same cell line) and ATAC- (blue, k-mers overlapping with **no** ATAC-seq peak in the same cell line) k-mers. Value is shown if at least 25% peaks at position are seen open/closed.

SPI1

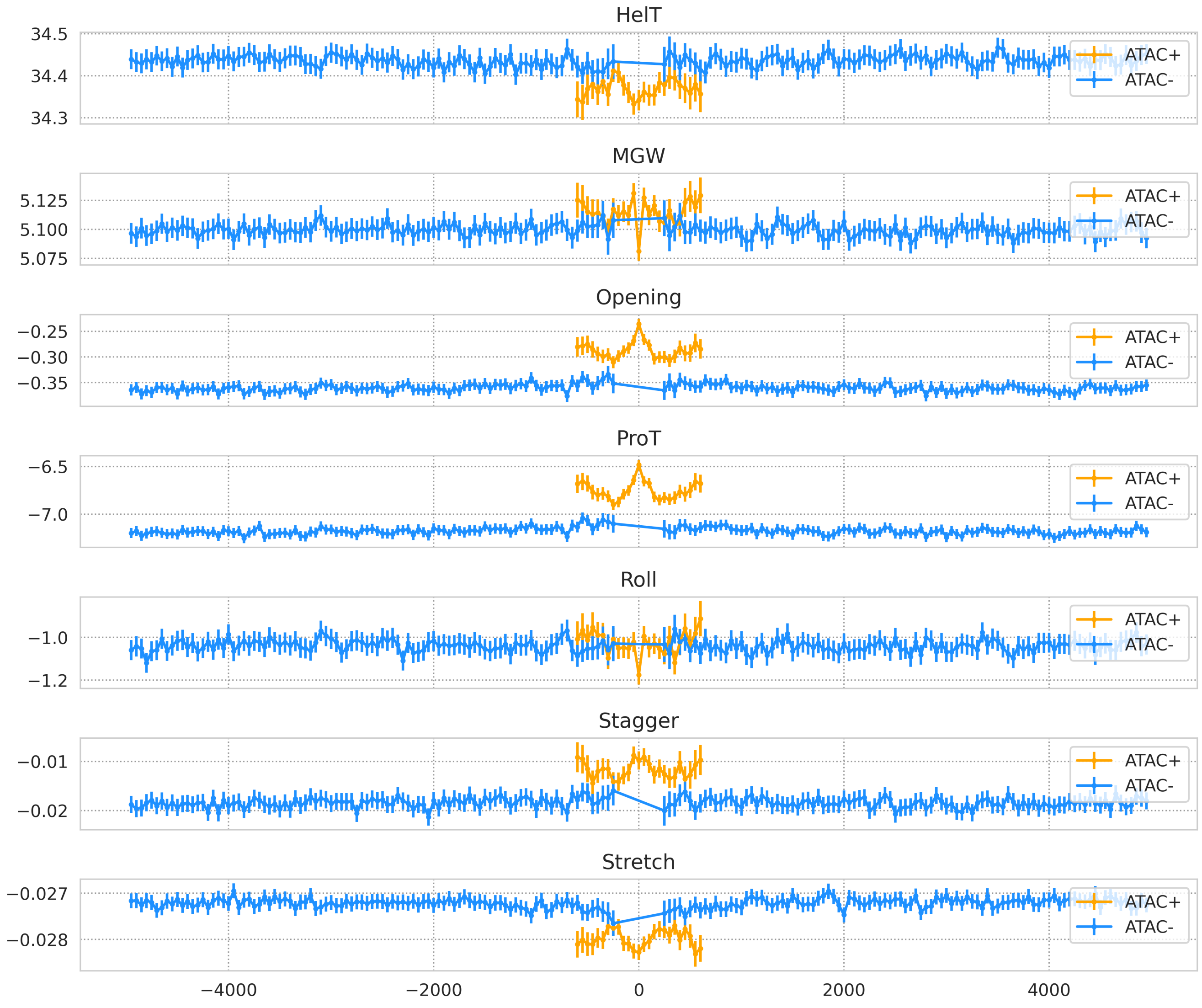

Fig. S32. Average DNA shape values (from top to bottom: helical twist, minor groove width, opening, propeller twist, roll, stagger, stretch) per every 25th offset for prolonged peaks in experiments targeting SPI1. We distinguish ATAC+ (orange, k-mers overlapping with an ATAC-seq peak in the same cell line) and ATAC- (blue, k-mers overlapping with **no** ATAC-seq peak in the same cell line) k-mers. Value is shown if at least 25% peaks at position are seen open/closed.

TP53

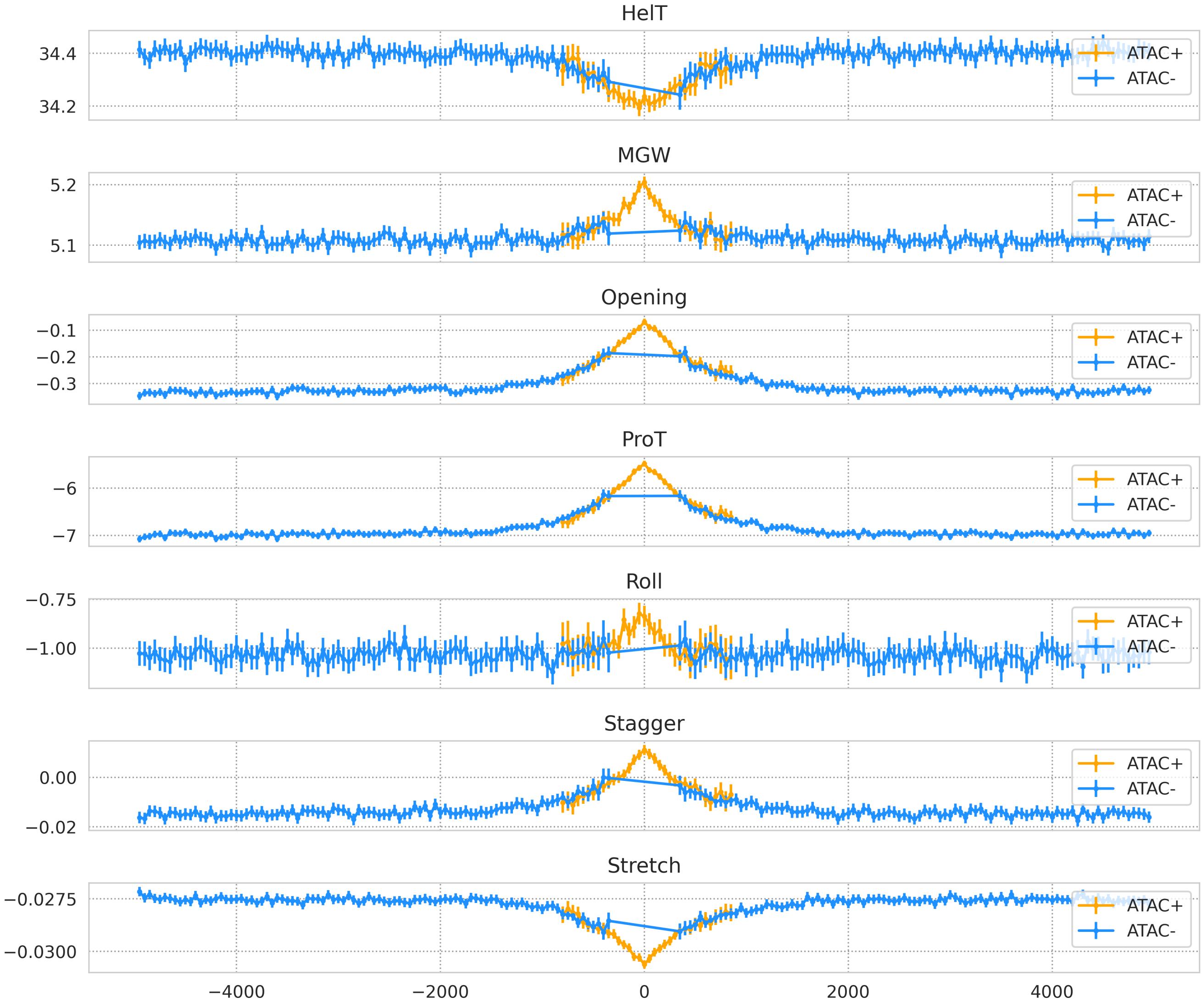

Fig. S33. Average DNA shape values (from top to bottom: helical twist, minor groove width, opening, propeller twist, roll, stagger, stretch) per every 25th offset for prolonged peaks in experiments targeting TP53. We distinguish ATAC+ (orange, k-mers overlapping with an ATAC-seq peak in the same cell line) and ATAC- (blue, k-mers overlapping with **no** ATAC-seq peak in the same cell line) k-mers. Value is shown if at least 25% peaks at position are seen open/closed.

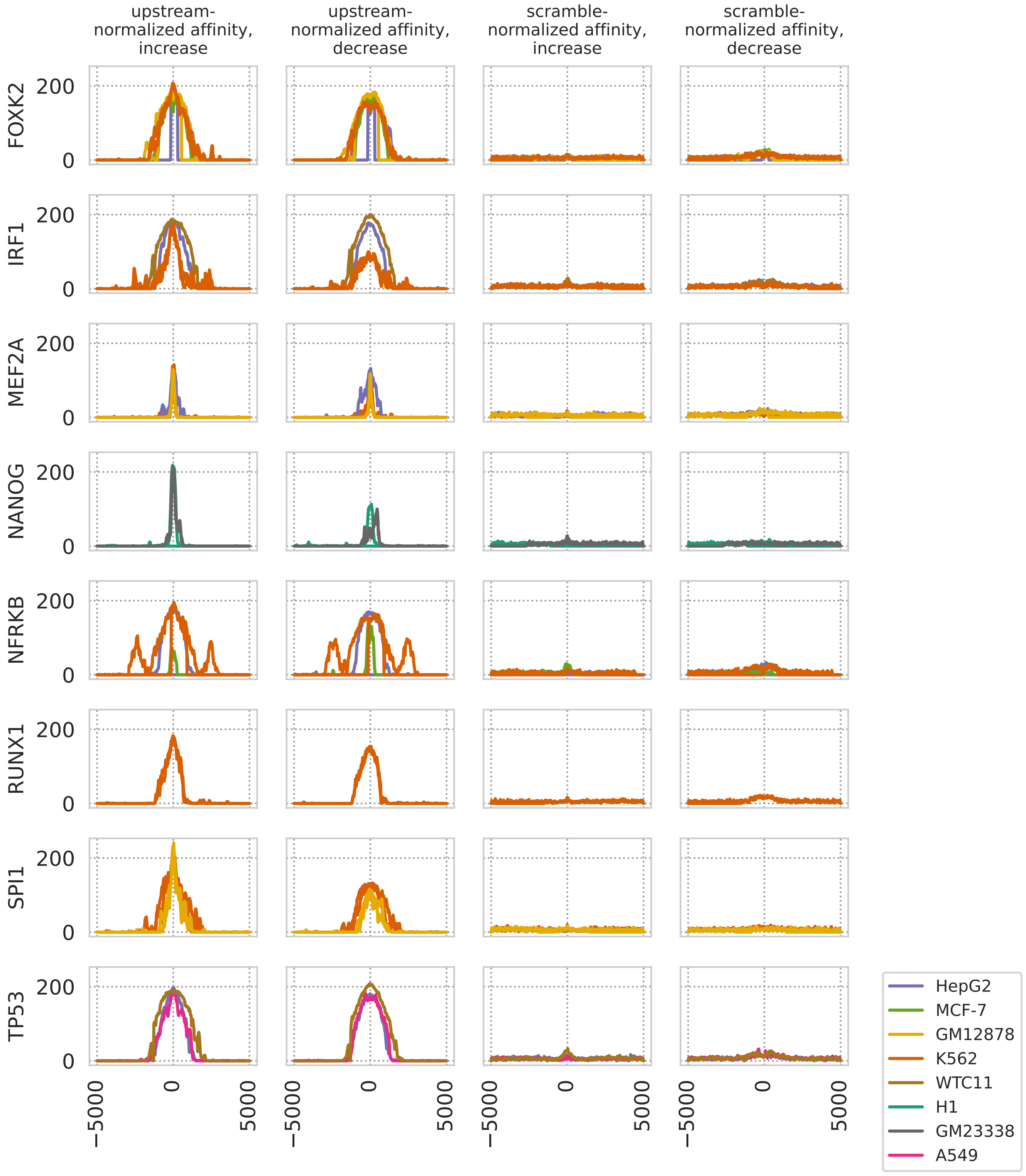

Fig. S34. First column: counts of TFs from the HOCOMOCO with increased upstream-normalized affinity when compared to prolonged peak edges, for each tested TF. Second column: counts of TFs from the HOCOMOCO with decreased upstream-normalized affinity when compared to prolonged peak edges, for each tested TF. Third column: counts of TFs from the HOCOMOCO with increased scramble-normalized affinity when compared to prolonged peak edges, for each tested TF. Fourth column: counts of TFs from the HOCOMOCO with decreased scramble-normalized affinity when compared to prolonged peak edges, for each tested TF. Each line color represents a different cell line. For a TF from the HOCOMOCO database, we assess the normalized affinity increases and decreases using the cluster analysis (Methods). The upstream-normalized affinity score is calculated as the k-mer affinity z-score with regards to affinity to sequences 5000 bases upstream of annotated transcription starts of RefSeq genes (hg19). The scramble-normalized affinity score is calculated using a bootstrapped set of k-mers found at a given offset from the putative binding site. Each of these k-mers is then randomly shuffled.

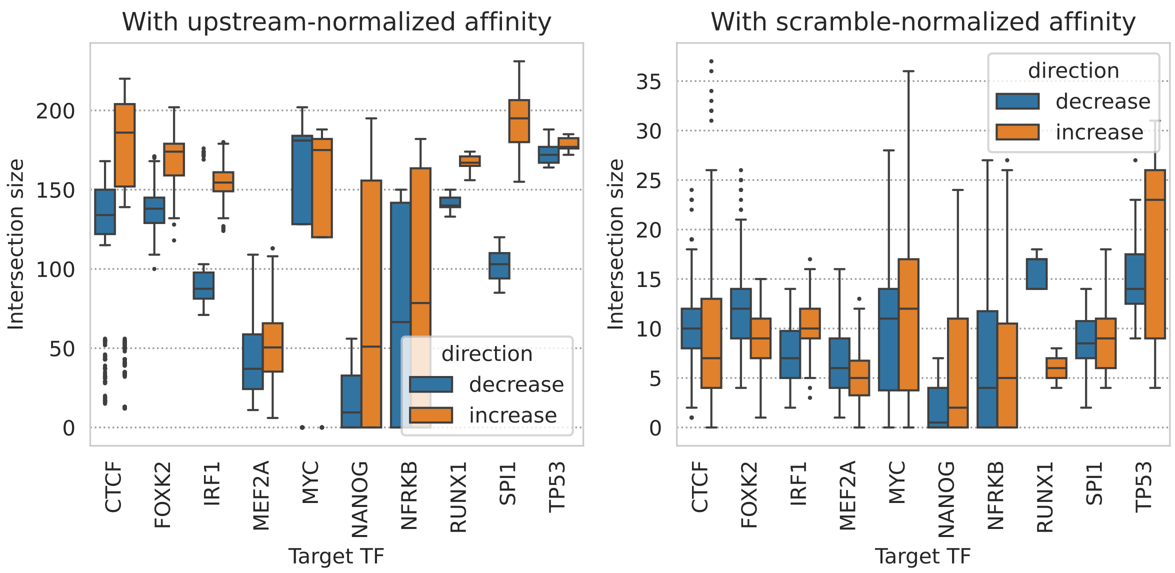

Fig. S35. Boxplots showing the distribution of intersection sizes for TFs from the HOCOMOCO database with altered normalized affinity between pairs of experiments targeting the same TF. Each data point represents the maximum number of overlapping TFs exhibiting differential affinity between two such experiments, for offsets up to 100 bp from the putative binding size. The left plot corresponds to the upstream-normalized affinity values, the right one to the scramble-normalized affinity values.

Table S13. Portion of experiments that displayed increased/decreased scramble-normalized affinity for a given HOCOMOCO TF at a given offset from the putative binding site. Only HOCOMOCO TFs that were observed inat least 75% of experiments (for the target TF) for at least one offset value are shown. We limit ourselves to the experiment that target TFs in three of more cell lines.

			offset from binding site								
Target TF	Change direction	HOCOMOCO TF	-100	-75	-50	-25	0	25	50	75	100
CTCF	decrease	CPEB1	10/11	9/11	10/11	10/11	10/11	10/11	10/11	10/11	8/11
	decrease	ZN562	9/11	9/11	9/11	5/11	2/11	7/11	9/11	6/11	7/11
	decrease	ZN649	9/11	9/11	6/11	4/11	4/11	3/11	3/11	7/11	8/11
	decrease	ZNF90	8/11	9/11	7/11	6/11	5/11	3/11	6/11	7/11	7/11
	increase	OZF	-	2/11	3/11	6/11	9/11	9/11	3/11	1/11	1/11
	increase	ZBT7A	5/11	4/11	9/11	8/11	7/11	8/11	8/11	6/11	3/11
	increase	ZFP57	-	-	1/11	8/11	9/11	7/11	1/11	-	-
	increase	ZN611	1/11	3/11	6/11	8/11	10/11	9/11	5/11	3/11	2/11
FOXK2	decrease	CPEB1	7/8	7/8	7/8	5/8	3/8	4/8	5/8	7/8	7/8
	decrease	OZF	6/8	3/8	2/8	4/8	4/8	2/8	2/8	2/8	3/8
	decrease	ZN550	6/8	4/8	3/8	3/8	4/8	3/8	5/8	4/8	2/8
	decrease	ZNF90	5/8	7/8	6/8	4/8	5/8	5/8	5/8	6/8	4/8
	increase	MYCN	5/8	3/8	5/8	5/8	6/8	5/8	6/8	5/8	5/8
	increase	ZBT7A	3/8	4/8	4/8	5/8	6/8	6/8	6/8	5/8	4/8
IRF1	decrease	HXD10	1/4	-	3/4	3/4	2/4	-	2/4	2/4	3/4
	decrease	SIX6	1/4	1/4	2/4	2/4	3/4	3/4	2/4	1/4	-
	decrease	ZN443	1/4	-	-	1/4	1/4	2/4	3/4	2/4	1/4
	decrease	ZN649	1/4	1/4	2/4	1/4	1/4	-	2/4	3/4	2/4
	decrease	ZNF90	-	1/4	2/4	1/4	2/4	2/4	1/4	2/4	3/4
	increase	MYCN	2/4	2/4	1/4	2/4	3/4	3/4	3/4	2/4	2/4
	increase	OZF	2/4	2/4	2/4	3/4	3/4	3/4	2/4	3/4	2/4
	increase	ZN302	-	2/4	2/4	2/4	2/4	2/4	2/4	2/4	3/4
	increase	ZN430	2/4	2/4	3/4	3/4	3/4	3/4	1/4	-	1/4
	increase	ZN443	3/4	2/4	2/4	2/4	1/4	1/4	1/4	1/4	2/4
	increase	ZN611	2/4	3/4	4/4	4/4	2/4	3/4	2/4	3/4	3/4
	increase	ZN623	-	3/4	3/4	1/4	2/4	3/4	2/4	2/4	1/4

	increase	ZN649	2/4	2/4	2/4	2/4	3/4	2/4	-	1/4	1/4
MEF2A	decrease	HMGA1	1/5	2/5	2/5	1/5	1/5	1/5	2/5	2/5	4/5
	decrease	OZF	4/5	4/5	3/5	2/5	2/5	1/5	2/5	2/5	4/5
	decrease	ZFP57	2/5	-	-	-	2/5	2/5	1/5	2/5	4/5
	decrease	ZN248	5/5	3/5	3/5	2/5	2/5	2/5	3/5	4/5	4/5
	decrease	ZN562	3/5	4/5	3/5	4/5	4/5	3/5	4/5	3/5	3/5
	decrease	ZN649	4/5	3/5	3/5	3/5	2/5	2/5	3/5	4/5	5/5
	increase	MYCN	3/5	3/5	2/5	2/5	3/5	5/5	5/5	2/5	-
	increase	ZN443	4/5	2/5	1/5	1/5	-	-	1/5	2/5	2/5
	increase	ZN550	3/5	1/5	1/5	3/5	4/5	2/5	3/5	3/5	1/5
	increase	ZN611	1/5	1/5	3/5	3/5	5/5	4/5	1/5	1/5	1/5
	increase	ZN623	-	-	3/5	4/5	4/5	3/5	1/5	-	1/5
MYC	decrease	CPEB1	7/8	5/8	5/8	6/8	6/8	7/8	6/8	5/8	6/8
	decrease	IRX2	4/8	4/8	5/8	4/8	6/8	6/8	5/8	4/8	4/8
	decrease	ZN580	6/8	6/8	4/8	5/8	6/8	5/8	6/8	6/8	4/8
	decrease	ZN736	5/8	3/8	1/8	3/8	5/8	7/8	5/8	6/8	5/8
	decrease	ZNF2	2/8	2/8	4/8	5/8	6/8	4/8	3/8	4/8	4/8
	decrease	ZNF90	6/8	4/8	2/8	3/8	3/8	3/8	4/8	4/8	5/8
	increase	MYCN	6/8	6/8	6/8	5/8	4/8	4/8	6/8	6/8	6/8
	increase	OZF	3/8	3/8	4/8	3/8	6/8	5/8	5/8	3/8	2/8
	increase	ZN302	4/8	2/8	2/8	4/8	5/8	5/8	5/8	6/8	3/8
	increase	ZN454	3/8	7/8	7/8	6/8	7/8	7/8	7/8	5/8	2/8
	increase	ZN578	2/8	3/8	5/8	6/8	7/8	5/8	4/8	4/8	4/8
	increase	ZN611	4/8	5/8	7/8	7/8	6/8	6/8	6/8	4/8	3/8
TP53	decrease	CPEB1	3/3	3/3	3/3	3/3	3/3	3/3	3/3	3/3	3/3
	decrease	HMGA1	1/3	1/3	3/3	2/3	1/3	1/3	1/3	2/3	2/3
	decrease	ZN248	3/3	2/3	2/3	2/3	2/3	2/3	1/3	3/3	3/3
	decrease	ZN550	1/3	1/3	2/3	2/3	1/3	2/3	3/3	3/3	3/3
	decrease	ZN562	2/3	2/3	3/3	3/3	2/3	1/3	2/3	3/3	2/3
	decrease	ZN580	2/3	2/3	3/3	1/3	2/3	2/3	2/3	3/3	2/3
	decrease	ZNF90	2/3	3/3	2/3	2/3	1/3	2/3	2/3	2/3	2/3
	increase	MYCN	3/3	2/3	2/3	2/3	3/3	3/3	3/3	2/3	3/3
	increase	ZN302	2/3	2/3	2/3	2/3	3/3	2/3	2/3	3/3	2/3
	increase	ZN623	1/3	-	-	1/3	2/3	3/3	3/3	3/3	1/3
